# Economic evaluation of physical activity mass media campaigns across the globe: a systematic review

**DOI:** 10.1186/s12966-022-01340-x

**Published:** 2022-08-26

**Authors:** Marina B. Pinheiro, Kirsten Howard, Cathie Sherrington, Adrian Bauman, Nathalia Costa, Ben J. Smith, William Bellew, Ding Ding, Anne Tiedemann, Belinda Wang, Andreia C Santos, Fiona Bull, Juana Willumsen, Bruna S. Albuquerque, Frances Rom Lunar, Vishwesh Bapat, Sarah K. Norris

**Affiliations:** 1 Institute for Musculoskeletal Health, The University of Sydney and Sydney Local Health District, King George V Building (Level 10N), Missenden Road, Camperdown, 2050 Australia; 2 Sydney School of Public Health, Faculty of Medicine and Health, The University of Sydney, Sydney, Australia; 3 School of Public Health, Faculty of Medicine and Health, Menzies Centre for Health Policy and Economics, Camperdown, Australia; 4 Charles Perkins Centre, Sydney School of Public Health, Faculty of Medicine and Health, The University of Sydney, Sydney, Australia; 5 Physical Activity Unit, Department of Health Promotion, Division of Universal Health Coverage and Healthier Populations, World Health Organization (WHO), Geneva, Switzerland; 6 Department of Physical Therapy, College of Allied Medical Professions, University of the Philippines, Manila, Philippines

**Keywords:** Mass media campaign, Physical activity, Cost-effectiveness, Economic evaluation, Systematic review

## Abstract

**Background:**

Physical activity mass media campaigns can deliver physical activity messages to many people, but it remains unclear whether they offer good value for money. We aimed to investigate the cost-effectiveness, cost-utility, and costs of physical activity mass media campaigns.

**Methods:**

A search for economic evaluations (trial- or model-based) and costing studies of physical activity mass media campaigns was performed in six electronic databases (June/2021). The authors reviewed studies independently. A GRADE style rating was used to assess the overall certainty of each modelled economic evaluation. Results were summarised via narrative synthesis.

**Results:**

Twenty-five studies (five model-based economic evaluations and 20 costing studies) were included, and all were conducted in high-income countries except for one costing study that was conducted in a middle-income country. The methods and assumptions used in the model-based analyses were highly heterogeneous and the results varied, ranging from the intervention being more effective and less costly (dominant) in two models to an incremental cost of US$130,740 (2020 base year) per QALY gained. The level of certainty of the models ranged from very low (*n* = 2) to low (*n* = 3). Overall, intervention costs were poorly reported.

**Conclusions:**

There are few economic evaluations of physical activity mass media campaigns available. The level of certainty of the models was judged to be very low to low, indicating that we have very little to little confidence that the results are reliable for decision making. Therefore, it remains unclear to what extent physical activity mass media campaigns offer good value for money. Future economic evaluations should consider selecting appropriate and comprehensive measures of campaign effectiveness, clearly report the assumptions of the models and fully explore the impact of assumptions in the results.

**Review registration:**

https://bit.ly/3tKSBZ3

**Supplementary Information:**

The online version contains supplementary material available at 10.1186/s12966-022-01340-x.

## Background

The health and social benefits of physical activity are well established [1]. Physical activity helps with the prevention and management of a range of chronic health conditions, such as coronary heart disease, diabetes and some cancers including bladder, endometrium, esophagus, kidney, lung, and stomach cancers [2]. Physical activity also has a positive impact on mental health, sleep, cognitive health, dementia and falls prevention [3–5]. Despite compelling evidence of the benefits of physical activity, high levels of physical inactivity continue to be observed worldwide with 25% of adults and 75% of adolescents globally not meeting the global recommendations for physical activity set by WHO [1, 6, 7]. Population-wide comprehensive strategies are urgently needed to address this issue.

Public education communication campaigns, also known as mass media campaigns, are recognised as one of the components of a comprehensive approach to promoting physical activity. Mass-media campaigns are mentioned in the policy recommendations outlined in the World Health Organization (WHO) Global Action Plan on Physical Activity (GAPPA) 2018-2030 [7]. The recommendation is to implement national and community-level communication campaigns as part of a comprehensive physical activity strategy. Mass media campaigns are also among the “Eight best investments for physical activity” developed by the International Society for Physical Activity and Health (ISPAH) [8].

There are several examples in the literature of mass media campaigns for physical activity promotion, such as the Verb campaign in the USA [9], Push Play campaign in New Zealand [10] and the ParticipACTION campaign in Canada [11]. They employed population-wide strategies, typically using a combination of the mass media communication channels of television, radio and print media [12–14]. These campaigns were intended to raise awareness of, improve knowledge about and change attitudes and social norms regarding physical activity, and ultimately influence behaviour change. They are commonly linked to other community-based initiatives, motivational and environmental programs, as well as other strategies to enhance message reach and support behavioural change in order to increase physical activity and examples include the use of pedometers, creation pf physical activity facilities and community-based programs.

Mass media campaigns can deliver specific physical activity messages to a large proportion of the population but do require substantial resources and costs. It is important therefore to establish whether these interventions offer good value for money. Cost-effectiveness analysis is a way to examine the costs and health outcomes of alternative interventions, revealing the trade-offs involved in choosing one intervention over another [15]. Cost-utility analysis is a form of cost-effectiveness analysis in which health effects are measured as multi-dimensional health outcomes that are reduced to a single index, such as quality-adjusted life years (QALYs) or disability adjusted life years (DALYs). The use of a single index provides a common metric that allows broader comparison to be made between treatments for different conditions and populations [16]. To the best of our knowledge there is no systematic review of economic evaluations of physical activity mass media campaigns currently available in the literature.

This review aims to summarise the evidence on economic evaluations and costing studies of physical activity mass media campaigns. It was commissioned by the WHO to inform the development of the WHO ACTIVE toolkits [17] which support countries to implement the policy recommendations outlined in the GAPPA 2018-2030 [7], and the updating of the WHO CHOICE modelling of cost effective interventions for physical activity [7, 18]. The review questions were:

1. What is the cost-effectiveness of physical activity mass media campaigns?
2. What are the costs of developing and implementing physical activity mass media campaigns?

## Methods

We followed the Preferred Reporting Items for Systematic Reviews and Meta-Analyses (PRISMA, Additional file 1: Appendix 1) and guideline recommendations for conducting systematic reviews of economic evaluations for informing evidence-based healthcare decisions [19–22]. A protocol was prospectively registered and published on the Open Science Framework website (https://bit.ly/3tKSBZ3) [23]. A glossary with definitions of key health economic terms to help understanding of the articles is available in Additional file 1: Appendix 2.

### Searches

We searched the following specialised databases and registries from inception to June 2021: Medline (Ovid), Embase (Ovid), the National Institute for Health Research Economic Evaluation Database (NHS EED, via Centre for Reviews and Dissemination (CRD) up to 2015), Health Technology Assessment (HTA) database (via CRD), Research Papers in Economics (RePEc, via EconPapers) and EconLit (Ebsco) (Additional file 1: Appendix 3). We also checked reference lists of other relevant systematic reviews as well as studies included in this review.

### Eligibility

#### Type of study

We included full (cost-effectiveness, cost-utility, or cost-benefit analysis) and partial (cost or cost-consequences analysis) economic evaluations of physical activity mass media campaigns. We used the WHO’s definition of physical activity (“any bodily movement produced by skeletal muscles” [7]). Physical activity mass media campaigns were defined as programs that used persuasive mass media communications to promote physical activity and persuade people to increase or adopt some form of physical activity. We considered both trial-based analysis, where all information used to perform the evaluation is obtained from intervention evidence from single source data, and model-based economic evaluations, where external sources are used to inform inputs for an analysis. We only included peer-reviewed manuscripts and relevant policy reports from trustworthy organisations (e.g. endorsed by governments, conducted independently from the funding body). We excluded systematic reviews and economic evaluations investigating interventions conducted in single settings (e.g., a physical activity campaign in a single school or a workplace). We did not apply any restrictions on publication date, language, or country.

#### Intervention and population

We included mass media campaigns that used mass media or public communications to persuade, inform, direct or motivate a population to think about, initiate or increase any type of physical activity. Examples of media included TV, radio and newspaper. We excluded narrow reach media, such as brochures and posters. We only included studies reporting media campaigns targeting a whole population or a population subgroup, which could be any age group, the general population, or people with existing conditions. Obesity or non-communicable disease prevention campaigns were included if physical activity was a clearly defined sub-component and physical activity outcomes were reported.

#### Outcomes

Eligible studies had to report at least one of the following outcomes: mass media campaign awareness, antecedents of physical activity (e.g., attitudes, intentions), and physical activity behaviour change. Incremental cost effectiveness ratio (ICER) was the main outcome of this review. ICERs could be expressed as the incremental cost per change in physical activity, or the incremental cost per QALY gained, or DALY avoided. Secondary outcomes included intervention costs. Additional details on the eligibility criteria are provided in Additional file 1: Appendix 4.

### Study selection and data extraction

Two independent reviewers screened all titles and abstracts, followed by full text screening. Any disagreements were discussed and a third reviewer was involved if needed to reach consensus. One reviewer extracted information into a standardised form and a second reviewer checked all data. For costing studies that reported multiple physical activity outcomes, we only extracted data for one selected outcome following this hierarchical order: i) physical activity behaviour; ii) physical activity antecedents, such as knowledge, attitudes, efficacy or intention; iii) mass media campaign awareness, campaign recognition, or campaign message understanding.

### Methodological quality assessment

Methodological quality assessment was conducted using the Extended Consensus on Health Economic Criteria list (CHEC-list) (Additional file 1: Appendix 5) [24, 25]. We created a modified version of the CHEC-list to assess the quality of the costing studies (Additional file 1: Appendix 6). The final version was reviewed and approved by three authors. Two independent reviewers rated each study, and a third reviewer was involved in case of disagreements. We considered the information provided in the included study as well as that from any other relevant publication cited in the study, such as an economic evaluation protocol or a second paper where the main trial results were reported.

We also identified additional items relevant to assessing the quality of included studies in the context of the present review questions that were not captured by the CHEC-list (Additional file 1: Appendix 7). All economic evaluations were rated using additional context-specific questions, referred to here as the ‘expanded CHEC-list’.

### Assessment of the certainty of model-based economic evaluations for WHO decision-making

We developed a Grading of Recommendations Assessment, Development, and Evaluation (GRADE) style rating to assess the overall quality of the model-based analyses (Additional file 1: Appendix 8). The GRADE style rating was created based on the concepts identified in the GRADE approach [26] as well as previous recommendations for assessing the certainty of evidence from modelling studies [27]. The following domains were considered: A) Quality of model reporting, B) Certainty of model inputs, C) Credibility of model, D) Certainty of model outputs, E) Directness of model. Each domain was rated as “poor”, “fair” or “good” (Additional file 1: Appendix 8, Table 7.A). The overall certainty of each economic model for WHO decision-making was rated as high, moderate, low, or very low by considering the ratings for the individual domains (Additional file 1: Appendix 8, Table 7.B).

### Data synthesis

As the included studies were heterogenous in their interventions, methods, data and context, pooling of results was considered inappropriate. Consequently, we present a narrative synthesis of the findings from included studies. Summaries of effect size, cost-effectiveness and costs are reported for each study (where available).

Studies reported costs in different currencies and from different years. Therefore, we expressed monetary values in two ways: i) by year and currency as reported by the authors of the included study, ii) converted to 2020 US dollars to enable comparison of findings. We initially inflated the costs to 2020 using the inflation rate for each country according to inflation rates from the OECD database [28]. Then we transformed the costs in respective currencies into US dollars using purchasing power parity (PPP) conversion factors for 2020 [29].

## Results

**Fig. 1:**
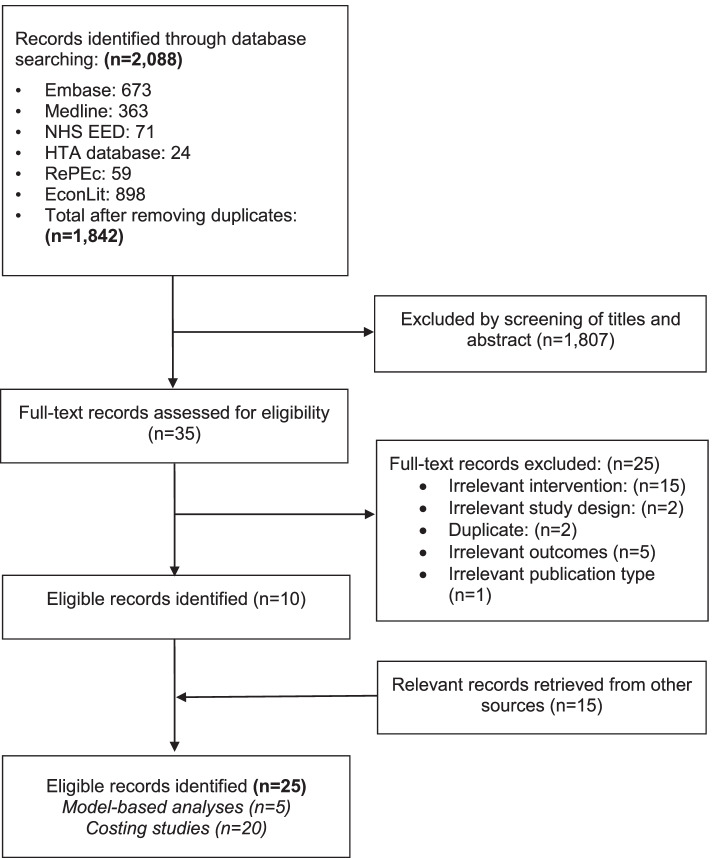
Flow chart of selection of studies investigating economic evaluations and costs of physical activity mass media campaigns

The electronic search yielded 2088 records. An additional 15 studies were identified from hand searching, including screening of other systematic reviews in the field and contact with experts in the field (Fig. 1).

A total of 25 studies, five model-based analyses [30–34] and 20 costing studies [10, 35–53], were included in this review (Additional file 1: Appendix Table 1). The records excluded at full-text screening and the reasons for exclusion are presented in Additional file 1: Appendix 9.

### Question 1: what is the cost-effectiveness and cost-utility of physical activity mass media campaigns?

#### Study characteristics

We found five model-based analyses investigating the cost-effectiveness of mass media campaigns (Table 1). The types of models used varied across studies and included Markov model [31, 34], OECD SHPeP-NCD (microsimulation 31 years) [32] and Multistate lifestate model [33]. One study did not provide model details [30]. The campaigns varied in terms of type, target population, duration and whether they targeted the regional or national level (Additional file 1: Appendix Table 1). The target populations of the campaigns were adults (*n* = 3, age range 25-64 years) and both adults and older adults (*n* = 2, age range 15-79 years) (Additional file 1: Appendix Table 2). These models were all applied to high-income country populations (United States, Australia, New Zealand, Belgium and Italy). The geographical location of the campaigns is presented in Additional file 1: Appendix Table 3. Four studies applied the model to the whole country [30, 32–34] whereas one study applied the model to one city in Belgium (Ghent) [31].

**Table 1:** Summary of results of the model-based economic evaluations investigating physical activity mass media campaigns

Author (year), Campaign	Perspective/ Time horizon	Population	Campaign	US$^a^/QALY or DALY	Cost-effective considering local threshold^b^	Plane	Level of certainty^c^
Roux (2008), Wheeling Walks [34]	Societal/40 years	United States adult population (25-64 yrs)	One-off 8-week community-wide intervention with mass media campaign	$20,099/QALY gained	Yes	NE	LOW
Cobiac (2009), Exercise, you only have to take it regularly not seriously [30]	Health sector perspective/Lifetime of the population in 2003	Australian population (25-60 yrs)	One-off 6-week mostly mass media campaign with some community support	Cost saving -Cost Offsets (total): -$931 million (95% UI -$1176 to -$281) -QALY gained (total): 23,000 (95% UI: 7600 to 40,000)	Yes	SE	LOW
De Smedt (2011), 10,000 Steps Ghent [31]	Public payer/20 years (cycle length of 1 year)	Population of a mid-sized city in Belgium (25-75 yrs)	Life-long pedometer-based community-wide intervention with mass media campaign, with a 5-year cycle where the pedometer was implemented in year one	*Women*: Cost-saving -Cost offsets (average): -$386.61 -QALY gained (average): 0.11	Yes	SE	LOW
				*Men*: Cost-saving -Cost offsets (average): -$521.51 -QALY gained (average): 0.16			
Goryakin (2019), Hypothetical campaign [32]	Health system / 31 years	Italy population (18+ yrs)	18 weeks duration with 6 segments in 31 years hypothetical mostly mass media campaign	$21,713/DALY	Yes	NE	VERY LOW
Mizdrak (2020), Hypothetical campaign [33]	Health system/ Lifetime of the population in 2011	New Zealand adult population (15-79 yrs)	One-off hypothetical mass media campaign to promote smartphone apps for physical activity	$130,740/QALY gained (95% UI 18,989 to 385,367)	No	NE	VERY LOW

*NE* north-east quadrant, indicates that the intervention is more costly and more effective, *SE* south-east quadrant, indicates that the intervention is less costly and more effective, *UI* uncertainty interval

^a^In 2020 US Dollars

^b^Implicit threshold values given by WHO team (see Additional file 1: Appendix 10 for more details and references). We only considered whether the point estimates fell within the threshold and did not consider uncertainty intervals as they were commonly missing

^c^Overall judgement of certainty of each economic model for WHO decision-making according to a GRADE style rating (see Additional file 1: Appendix 13 for more details)

The campaigns investigated varied and included: i) one mostly comprised of mass media (“Exercise, you only have to take it regularly not seriously”), ii) two community-wide programs supported by mass media campaigns (“10,000 steps Ghent” and “Wheeling Walks”), iii) a hypothetical mass media campaign following the principles developed in the campaign “Exercise, you only have to take it regularly not seriously”, and iv) a hypothetical mass media campaign promoting the use of physical activity apps. Only three studies reported the baseline physical activity level of the population and only one study reported the impact of the campaign on physical activity in relation to baseline physical activity levels. The impact of physical activity was expressed using different measures, such as Metabolic Equivalent (MET)-min/week (*n* = 2), moderate-vigorous physical activity (MVPA) MET-min/week (*n* = 1), walking min/week (*n* = 1) and percent increase in people considered at least moderately active (*n* = 1) (Additional file 1: Appendix Table 1).

Additional file 1: Appendix Table 4 presents a description of the approach to model-based analyses used by the five studies. The methods and assumptions used were highly heterogeneous, particularly in terms of selection of physical activity effectiveness measures and assumptions regarding the attenuation of physical activity impact over time. Three studies [30, 31, 34] used the physical activity impact estimates from effectiveness studies, one [32] used the median effectiveness of previous mass media campaigns reported in 12 papers included in a literature review [54], and one [33] used the estimate from a systematic review of interventions that used apps or pedometers to promote physical activity. Overall, the future health benefits of physical activity assumed by the authors were similar, but the reporting of the parameters used (and their sources) was incomplete for most studies. Three studies used parameters that were relevant and appropriate for the population investigated. One study used parameters that were to some extent relevant to the population. One study did not report the parameters and sources, so it is unclear if they were appropriate for the modelled population.

#### Cost-effectiveness results

The most common perspective adopted was a health system perspective (*n* = 3) [30, 32, 33], with one [34] study adopting a societal perspective and one [31] a public payer perspective. The time horizon of the models ranged from 20 years to the lifetime of the population investigated. All studies reported the results as additional cost per QALY gained (*n* = 4) [30, 31, 33, 34] or DALY avoided (*n* = 1) [32]. Most studies (*n* = 4) did not report the questionnaires used to generate QALYs and DALYs and one study reported using EQ-5D [31] and used Belgian utility data. The results of the model-based cost-effective analyses studies varied. They ranged from the intervention being more effective and less costly (dominant) in two models [30, 31] to an incremental cost of $130,740 (US 2020) per QALY gained (Table 1) [33]. The results of four models [30–32, 34] were considered to show acceptable cost-effectiveness according to implicit thresholds for willingness to pay for each country, as provided by the WHO (Additional file 1: Appendix 10), with Mizdrak (2020) being the only exception (Table 1). Additional file 1: Appendix Table 5 provides additional details on the main economic evaluation findings of the five model-based analyses. Additional file 1: Appendix Table 6 provides a description of cost items and valuation sources used in the model-based analyses.

#### Methodological quality and certainty of the evidence

Overall, the quality of the model-based analyses, based on the extended CHEC-list, ranged from 11 to 16 out of 20 (Additional file 1: Appendix Table 7). General limitations in the economic evaluations included: limited uncertainty analyses, lack of transparency in the reporting of modelling methods, and lack of systematic methods to identify relevant outcomes and to appraise the quality of the sources of clinical evidence.

Table 2 presents the included studies authors’ conclusions and detailed reviewers’ comments on the approach to the model-based analyses. Overall, studies made overly optimistic assumptions about the sustainability of campaign effects beyond the campaign period, selected inappropriate measures of effectiveness, failed to explore all relevant parameters in sensitivity analyses, did not report all parameters used in the model as well as their sources. In addition, they only reported results for the end of the time horizon investigated instead of presenting intermediate measures which would enhance the interpretability of the findings (Additional file 1: Appendix Table 8).

**Table 2:** Authors’ conclusions and reviewers’ comments on the approach to model-based analysis of physical activity MMCs

Author (year), Campaign name	Authors’ conclusions about cost-effectiveness of the mass media campaign	Comments
Roux (2008),Wheeling Walks [34]	The intervention appeared to reduce disease incidence, to be cost-effective, and—compared with other well-accepted preventive strategies—to offer good value for money. The results support using the evaluated intervention as part of public health efforts to promote physical activity.	*Effectiveness measure appropriate*: No. Effectiveness study targeted older adults (50–65 years) from a small community in West Virginia, effectiveness is being generalised to adults 25 to 64 years. *Attenuation of PA impact*: The impact of the intervention was assumed to decline after the intervention had ended and a 50% decline in PA in year 2 was modelled. Estimate seems to be optimistic, and no evidence was given to support this assumption. *Intermediate measures reported*: No but reported sensitivity analysis for time-horizon from 40 to 30, 20 or 10 years. *Uncertainty explored*: Yes but results for sensitivity analysis exploring uncertainty in utility values, costs and effect of PA on health NR. Sensitivity analysis varying the dissipation of the effect size of the interventions had a marginal impact on results, but the details of the parameters used, and results NR. *Other*: Community wide program with strong mass media campaign support. Additional components included: primary care initiatives, community programs, cross sectoral initiatives, website resources, worksites.
Cobiac (2009), Exercise, you only have to take it regularly not seriously [30]	Physical activity promotion intervention is recommended as a public health measure. Despite substantial variability in the quantity and quality of evidence on intervention effectiveness, and uncertainty about the long-term sustainability of behavioural changes, it is highly likely that as a package, all six interventions could lead to substantial improvement in population health at a cost saving to the health sector.	*Effectiveness measure appropriate*: Yes *Attenuation of PA impact*: Base case analysis assumed that the intervention effects on PA are sustained for year 1, but decay exponentially (rate of 50% per annum) thereafter. Optimistic assumption that intervention effects would last for one year considering this was a 6-week one-off intervention. Assumption was not explored in a sensitivity analysis. *Intermediate measures reported*: No *Uncertainty explored*: Yes, but only for attenuation of PA impact (0 to 100% decay). Results were robust, intervention remained dominant. Sensitivity analysis exploring uncertainty in utility values, costs, and effect of PA on health not performed. *Other*: Campaign was mostly a mass media campaign with some community support.
De Smedt (2011), 10,000 Steps Ghent [31]	The community-based ‘10,000 Steps Ghent’ campaign is a dominant intervention. Sensitivity analyses have proved the robustness of the results; hence implementing this intervention on a population-based level could lead to improved health outcomes and reduced costs.	*Effectiveness measure appropriate*: Yes *Attenuation of PA impact*: Base case model assumes life-long programme with a life-long intervention effect. 5-year cycle used for the intervention where the intervention had to be implemented each year. Pedometers were implemented only in year 1 and the effects were expected to last for 5 years. In years 2-5 only the MM components were implemented. This cycle was repeated for the duration of the model. Effectiveness data used was from a 1-year intervention. It is unclear if the same level of effectiveness would sustain in years 2 to 5 where only the MM components were used. Possible increment over time with message reinforcement following a MMC not explored (as there were 6 segments of the campaign). Sensitivity analyses performed for 1-year intervention effects still showed favourable results, however there was a substantial decrease in QALY gain and cost savings. *Intermediate measures reported*: scenario analysis presented for 1-year, 5-year and 20-year intervention effects. *Uncertainty explored*: Yes, around intervention effects, utility, risk reduction, intervention costs and colon cancer costs. Uncertainty around risk reductions had the biggest impact on the results, but the intervention remained cost-saving. Other health care costs not explored. Probabilistic analysis showed 100% of the intervention being cost-saving and more effective. *Other*: 1) QALY effect was small: average QALY gained: 0.16 (men) and 0.11 (women) at 20 years; 2) Unclear how intervention costs were calculated. Community walking intervention with some MM promotion which needs to be considered when interpreting the results.
Goryakin (2019), NR (Hypothetical campaign) [32]	Investing in policies to promote active lifestyles is a good investment for Italy	*Effectiveness measure appropriate*: Unclear. Used median effectiveness of 12 previous campaigns identified in a review conducted by the authors. However, these previous studies investigated different types of campaigns and various durations. *Attenuation of PA impact*: Stated that model takes this into account but details not provided. 6 segments of three years each in 31 years. Effectiveness estimate changed over time (60% increase after 1 month, drop to 30% after 1 year, drop to 0 after 2 more years). Unclear how the 35.55% effect at the end of the intervention was used to calculate the attenuation. Median intervention duration of the 12 studies (18 wks) used by authors to calculate the effectiveness estimate. Therefore, the drop to 30% after 1 year might be optimistic. Authors did not consider or explore the possibility of effect increment over time with message reinforcement (as there were 6 segments of the campaign). For the less inactive individuals, probability of moving into the new PA category is higher. However, the opposite would be expected (i.e. the less active have a higher probability of moving into a new PA category). *Intermediate measures reported*: ICER across the years are presented in a figure. Estimates are available from 2025, 2037 and 2050 (provided by the authors upon request from review authors). *Uncertainty explored*: methods for dealing with uncertainty stated in the report describing the model, however no results presented. Therefore, it is unclear if the results are robust and how much each parameter would impact on the results. *Other*: Study found a significant impact of the intervention on the burden of disease; however the intervention effect was small. Intervention cost items and details NR. Since this is a hypothetical MMC the extent to which other components contributed to the effectiveness estimate is unclear. Amongst the 12 studies used to estimate the effectiveness there was a range of types of MMCs, including stand-alone campaigns as well and community and cross-government programs. The lack of clarity of the content of the intervention impacts the interpretation of results.
Mizdrak (2020), NR (Hypothetical campaign to promote apps for physical activity) [33]	A mass media campaign to promote smartphone apps for physical activity is unlikely to generate much health gain or be cost-effective at the population level. Other investments to promote physical activity, particularly those that result in sustained behaviour change, are likely to have greater health impacts.	*Effectiveness measure appropriate*: No. Evidence for campaign effect on PA levels derived from a systematic review where the meta-analysis included studies investigating an app or wearable device with additional components (e.g. counselling, goal setting, education). Therefore, it is not possible to know the effect of app/pedometer use as a standalone intervention. Additionally, the studies included in the review were mostly small and conducted in controlled environments. The effectiveness measure used was 285 MVPA-MET min/wk.(equivalent to 1.6 hours of additional brisk walking per week). It is an optimistic assumption that such gains would result from using an app without any additional support. *Attenuation of PA impact*: Considered change to app adherence over 1-year but did not consider reduction of PA effect over 1-year. The median intervention duration of the studies included in the meta-analysis used to estimate effectiveness was 8 weeks, therefore it is unclear if the effects would sustain for a year. *Intermediate measures reported*: No *Uncertainty explored*: Yes. Sensitivity analysis explored intervention parameters, sustainability of the intervention, narrowing target population and discount rate. Overall the analysis reviewed big uncertainty in the results. No sensitivity analysis was performed to explore the uncertainty of utility values, health costs, effect of PA on health. *Other*: Source for utility values and how it was included in the model NR. Very small improvement in QALY.

*DALY* Disability-adjusted life year, *ICER* Incremental cost-effectiveness ratio, *MM* mass media, *MMC* Mass media campaigns, *PA* Physical activity, *QALY* Quality-adjusted life year, *MVPA-MET min/week* Moderate to vigorous Physical Activity-Metabolic equivalent per minute per week, *NR* not reported

Our GRADE style rating revealed that the level of certainty of the models ranged from very low (*n* = 2) to low (*n* = 3). This indicatesthat we have very little or little confidence that the outputs from the model are reliable for decision making (Additional file 1: Appendix Table 10).

### Question 2: what are the costs of physical activity mass media campaigns?

All but one of the model-based analyses (*n* = 5) [34] reported intervention costs. All of the 20 identified costing studies [10, 35–53] reported intervention costs only, without performing an economic evaluation (Additional file 1: Appendix Table 1). The campaigns targeted adults (*n* = 8) [10, 37, 39, 41, 42, 44, 49, 50], both adults and older adults (*n* = 5) [35, 43, 45, 48, 51], older adults only (*n* = 1) [36], adolescents (*n* = 1) [38], children (*n* = 1) [46], and people across the lifespan (*n* = 4) [40, 47, 52, 53] (Additional file 1: Appendix Table 2).

All costing studies were conducted in high-income countries except one campaign that was conducted in a middle-income country (Brazil). The geographical location of the campaigns investigated in the costing studies is summarised in Additional file 1: Appendix 4. Amongst the costing studies, the campaign approaches varied and were classified as: mostly mass media (*n* = 9), community-wide intervention with a mass media campaign (*n* = 6), mostly community-wide intervention with supportive mass media or media promotions (*n* = 3), and mass media promotions for single-day events, trails or parks (*n* = 2) (Table 3). The quality of the costing studies ranged from 3 to 9 out of 15 (median = 6, Additional file 1: Appendix Table 9).

**Table 3:** Summary of results of the costing studies investigating physical activity mass media campaigns

Campaign names	Countries	# studies reporting total costs	Cost/week (range in US 2020 where available)	Reported impact on PA (# studies with a + ve impact)
**Mostly mass media**				
ACTIVE for LIFE Find Thirty every day Make healthy normal Measure-up ParticipACTION’s 150 Play List Push Play Step-up Hawaii VERB campaign	Australia (*n* = 4), Canada (*n* = 1), New Zealand (*n* = 1), United Kingdom (*n* = 1), United States (*n* = 2)	6 (2 studies where it was unclear if there were other costs that contributed to the campaign total cost)	$14,284 to $1,951,906 (*n* = 9)	PA behavior: 5/7 PA antecedents: 1/1 Campaign awareness: 0/1
**Community-wide interventions with mass media**				
10,000 Steps Rockhampton Agita São Paulo BC Walks Get up and do something Make a Move Walk Missouri	Australia (*n* = 2), Brazil (n-1), United States (*n* = 3)	3 (1 study where it was unclear if there were other costs that contributed to the total campaign cost)	$27,374 to $56,667 (*n* = 3)	PA behavior: 4/6 PA antecedents: n/a Campaign awareness: n/a
**Mostly community-wide interventions with supportive mass media or media promotions**				
Activate Omaha Good for Kids To be young at heart - Stay active Stay independent	Australia (*n* = 2) United States (*n* = 1)	3	$5050 to $307,495 (*n* = 3)	PA behavior: 2/2 PA antecedents: n/a Campaign awareness: 1/1
**Mass media promotions for single day events, trails, parks**				
Happy trails Walk to Work Day	Australia (*n* = 1), United States (*n* = 1)	2	$14,598-$189,391 (*n* = 2)	PA behavior: 2/2 PA antecedents: n/a Campaign awareness: n/a

Only campaign category types for which costing studies were included are listed in this table

*PA* physical activity, *n/a* information not available in Additional file 1: Appendix Table 1

For costing studies that reported multiple physical activity outcome measures we only extracted data for one outcome following this hierarchical order: i) physical activity behaviour; ii) physical activity antecedents, such as knowledge, attitudes, efficacy or intention; iii) mass media campaign awareness, campaign recognition, or campaign message understanding

For additional information on intervention costs please see Table 4 and Additional file 1: Appendix Table 10

Overall, intervention costs were poorly reported across the 25 included studies, with only Eight (32%) [10, 30, 33, 37–39, 43, 45] reporting the total costs as well as the costs of the items contributing to the total costs (Table 4). Eight studies (32%) [32, 36, 41, 44, 46, 47, 49, 53] reported total costs only, without giving details of how the costs were derived. Three studies (12%) [40, 48, 51] reported some costs, but it is unclear if all costs involved in developing and implementing the intervention were considered. Additional file 1: Appendix Table 10 provides a detailed description of total cost, cost per item, cost in the reported currency and year, as well as costs transformed to 2020 US dollars, and cost per week for each of the campaigns.

**Table 4:** Intervention cost items measured and reported in the studies investigating physical activity mass media campaigns

Author (Year), Campaign name	Total Costs	Development	Development and dissemination	Materials	Dissemination							Staff	Other	In-kind contributions (amount NR)
					***Overall***	***TV***	***Radio***	***TV +*** ***Radio***	***Website/Social Media***	***Billboards***	***Newspaper***			
**MODEL-BASED**														
**Mostly MM**														
Cobiac (2009), Exercise, you only have to take it regularly not seriously¦ [30]	^*^	^*^		^*^		^*^							^*^	
**CWI with MMC**														
Roux, (2008), Wheeling Walks [34]														
De Smedt (2011), 10,000 Steps Ghent¦ [31]	^*^			^†^					^†^			^†^	^†^	
**OTHER (HYPOTHETICAL CAMPAIGN)**														
Goryakin (2019), NR [32]	^*^													
Mizdrak (2020), NR [33]	^*^				^*^				^*^				^*^	
**COSTING STUDIES**														
**Mostly MM**														
Hillsdon (2001), ACTIVE for life [48]						^*^								
Bauman (2003), Push Play [10]	^*^	^†^												^*^
Huhman (2010)^§^, VERB campaign [46]	^*^													
Buchthal (2011), Step it up, Hawaii [50]	^Ϫ^			^*^		^*^	^*^							
Leavy (2013), Find Thirty every day [41]	^*^													
King (2013), Measure-up [44]	^*^													
Kite (2018)^¥^, Make healthy Normal [43]	^*^	^*^			^*^									
Kite (2020)^¥^, Make Healthy Normal [42]	^*^					^*^	^†^		^†^				^†^	
Berry (2020), ParticipATICON’s 150 Play List [52]	^Ϫ^				^*^								^*^	
**CWI with MMC**														
Mahecha Matsudo (2003), Agita São Paulo [40]				^*^								^*^	^*^	^*^
Wray (2005), Walk Missouri [35]	^Ϫ^			^*^			^*^			^*^	^*^			
Brown (2006), 10,000 steps Rockhampton [51]			^*^		^*^									^*^
Reger-Nash (2006), BC Walks [37]	^*^					^*^	^*^				^*^	^*^		
Stackpool (2006), Make a Move [36]	^*^													
Peterson (2008), Get up and Do something [38]	^*^					^*^				^*^				
**Mostly CWI with supportive MM or media promotions**														
John-Leader (2008), Stay active Stay independent [45]	^*^	^*^		^*^		^*^			^*^		^*^	^*^	^*^	^*^
Huberty (2012), Activate Omaha [47]	^*^													
Bell (2013), Good for kids [53]	^*^													
**MM promotions for single day events, trails, parks**														
Merom (2005), Walk to Work Day [39]	^*^							^*^						
Clark (2015), Happy trails [49]	^*^													

*CWI* community-wide intervention, *MM* mass media, *MMC* mass media campaign, *NR* not reported

^*^Costs were considered and reported for this item

^†^Costs were considered but not reported for this item

^Ϫ^Total cost reported by the authors, but it is unclear if there were other costs that contributed to the campaign total cost

^a^Mass media campaigns that were supported by additional components (e.g. pedometers, websites, community programmes) and reported their costs. NR: Not reported

^b^Studies which report findings of the Make Healthy Normal Campaign

^c^Studies which report findings of the VERB Campaign

As limited information on costs was available for the only campaign conducted in a middle-income country (Agita São Paulo), we have reported additional information about this program, from grey literature, in Additional file 1: Appendix 12.

## Discussion

There are few economic evaluations of physical activity mass media campaigns available in the literature; all are model-based analyses, and none were conducted in a low, low-middle or middle-income country. The ICER estimates ranged from two interventions being found to be more effective and less costly (dominant) [30, 31], to the highest base case ICER of US$130,740 per QALY gained (US dollars price year 2020) [33]. The level of certainty of the models ranged from very low to low, indicating that we have very little or little confidence that the outputs from the model are reliable for decision making. Therefore, it is unclear to what extent physical activity mass media campaigns offer good value for money.

Overall, it is difficult to estimate the impact of the campaigns on physical activity as studies used different definitions and measures of physical activity, methods of collecting data, and statistical methods. Assessing the effectiveness and long-term benefits of complex population-level public health interventions, such as mass media campaigns, has intrinsic challenges as these interventions cannot be easily evaluated in randomised controlled trials. Additionally, the effectiveness estimates are potentially subject to confounding by other co-occurring community-level interventions. Most of the model-based analyses (*n* = 4) did not critically appraise the clinical evidence relied on for the modelling. These factors and others that may impact on the effectiveness estimates need to be considered when interpreting the results of the economic evaluations.

The studies included in this review reported on several types of campaigns. No pattern emerged by campaign type, so conclusions cannot be made regarding whether certain types of campaigns are typically more cost-effective than others. The wide range of campaign types, including community-wide interventions and mass media to support the use of a physical activity app, as well as the variety of additional components added to diverse mass media campaigns have made the interpretation of results difficult. It is expected that a mass media campaign conducted within a comprehensive physical activity strategy will be more likely to influence physical activity behaviour in large populations [55]. Unfortunately, it was not possible to explore whether this type of campaign was more cost effective given the small number of economic evaluations included in this review.

All the model-based analyses extrapolated short-term effectiveness results over the long-term to DALYs and QALYS by making assumptions about key parameters, such as attenuation of physical activity impact over time and impact on other health conditions. This introduced further uncertainty into the results reported. Although such assumptions are important for building a model to investigate the long-term impact of interventions, these assumptions were not clearly reported or fully explored in sensitivity analyses. For instance, typically the effectiveness of these campaigns was measured immediately at the end of the campaign, with all the models assuming that the full campaign effect would last for at least 1 year, and any loss in effect was only applied from the second year. It is unclear how sustainable behaviour changes generated by physical activity mass media campaigns are. There is a scarcity of studies reporting physical activity behaviour post-campaign duration, and these studies suggest no change from baseline [10, 56–58] or a decline since the post-campaign measure [10, 48, 59]. Therefore the assumption that the effects of the campaign will persist for any period of time beyond the campaign is not supported by the literature.

The results of the current review are aligned with previous reviews of economic evaluations of physical activity interventions, which commonly find scattered evidence [60, 61]. Previous reviews have also highlighted the lack of economic evaluations of physical activity interventions and challenges in interpreting and comparing their results due to the heterogenous methods used, poor reporting and low methodological quality [60, 62, 63]. A previous review conducted by our group investigating the cost-utility of physical activity interventions for older people found that the interventions ranged from cost-saving to $88,000/QALY (US dollar 2018), but all of the interventions investigated structured exercise programs [61], which precludes direct comparison with the results of the current review.

Overall, intervention costs of the mass media campaigns were poorly reported. There is no information on intervention costs for any low and low-middle income countries and only one study reported partial intervention costs for one middle-income country (Agita São Paulo”). We were able to retrieve additional information on the costs of the campaign by contacting the authors and performing a grey literature search. This information should be interpreted with caution as we were unable to find peer-reviewed publications reporting the data and limited information was provided, hindering our appraisal of the quality and usability of the data.

It is unclear whether there is an investment threshold for physical activity mass media campaigns, but it is believed that at least similar levels of investment to those used to fund tobacco control campaigns [64] would be required given the enduring and high prevalence levels of inactivity and the lack of policy levers that are currently available for tobacco. The Centers for Disease Control (CDC) have established investment standards for mass media campaigns for tobacco control [65] and indicated a per capita investment threshold of $1.24 in 2014 US dollar ($1.36 in 2020 US dollar). This threshold is higher than the cost per capita reported in the studies included in this review, which ranged from $0.02 to 0.75 US 2020 dollar. These findings highlight that it is likely that more funding would be needed for physical activity mass media campaigns to generate adequate results.

The overview of the intervention costs presented in this report should be interpreted in the context of the type of campaign, duration and reach. Policymakers and governments who are considering implementation of a physical activity mass media campaign can use this information to inform their budget and campaign planning. The intervention cost information can also be used as an input for future model-based analyses.

### Limitations

Although we used a comprehensive search strategy, it is possible that we may have missed economic evaluations of campaigns that were not published in peer-reviewed journals. We did not apply any language restrictions, but we only used keywords in English to search the databases. Therefore, we may have missed studies investigating mass media campaigns in lower- and middle-income countries if they were published in other languages and not indexed in the searched databases. Additionally, the GRADE-style rating was developed and modified for the current review by the authors but its use has yet to be evaluated.

### Recommendations for future research

Future modelled economic evaluations should consider selecting appropriate measures of campaign effectiveness, preferably from a systematic review, and making realistic assumptions about attenuation of physical activity effects that are supported by the available evidence. Additionally, future studies should also consider testing all the assumptions in sensitivity analyses, reporting the unit costs and the total cost of the campaigns, clearly reporting the parameters used as well as their sources. In addition to results for the end of the time horizon, authors should also report intermediate results to enhance the interpretability of the models, and these may include: cost-effectiveness considering only trial data, results after utility-weights are applied and results for when time horizon is extended.

Future primary studies investigating physical activity mass media campaigns should consider including a trial-based economic evaluation, particularly those conducted in low-and-middle-income countries. Future costing studies should include and present a detailed breakdown of the elements of an intervention, and these could be grouped into set-up, recurrent, and capital costs. More specifically, authors should report unit costs and all costs involved in the development (set-up) and implementation of physical activity mass media campaigns, such as materials (capital), media (e.g., TV, radio, Billboards), staff (recurrent) and in-kind contributions.

It is strongly recommended that future primary studies and reviews follow standard economic evaluation best-practice recommendations and reporting guidelines, such as the Consolidated Health Economic Evaluation Reporting Standards (CHEERS) statement. Future reviews could focus on social media campaigns as the cost-effectiveness of these may differ to traditional media campaigns.

## Conclusion

This review only found five economic evaluations of physical activity mass media campaigns and 20 costing studies reporting the costs of developing and implementing the intervention. All studies were conducted in high-income countries, with exception of one costing study that was conducted in a middle-income country. Overall, it is difficult to interpret the results of the economic evaluations given their high heterogeneity in terms of campaigns characteristics, methods and assumptions, ambiguous reporting and lack of sensitivity analyses exploring uncertainty. The level of certainty of the models were low and therefore it is unclear to what extent physical activity mass media campaign offer good value for money. The information regarding intervention costs should be interpreted considering the type, duration and reach of the campaign. This information can be used for planning future physical activity mass media campaigns or future models investigating the value for money of such campaigns.

## Supplementary Information

**Additional file 1: Appendix 1.** PRISMA checklists. **Appendix 2.** Glossary. **Appendix 3.** Search strategy. **Appendix 4.** Inclusion and exclusion criteria. **Appendix 5.** Quality assessment of economic evaluations included in the review using the Extended Consensus on Health Economic Criteria list (CHEC-list). **Appendix 6.** Quality assessment of costing studies using a modified version of the Consensus Health Economic Criteria List (CHEC-list). **Appendix 7.** Expanded CHEC-list: Additional questions on the quality of economic evaluations of physical activity mass media campaigns. **Appendix 8.** GRADE style rating for model-based economic evaluation of physical activity mass media campaigns. **Appendix 9.** Records excluded at full-text screening and reasons for exclusion. **Appendix 10.** Implicit thresholds for willingness to pay for each country provided by WHO team. **Appendix 11.** Application of the GRADE style rating to assess the certainty of each economic model for WHO decision-making. **Appendix 12.** Additional resources on Agita São Paulo campaign. **Appendix table 1.** Characteristics of studies included in this review according to study type: model-based analyses and costing studies. **Appendix table 2.** Mass media campaigns by target population. **Appendix table 3.** Mass media campaigns by geographical location. **Appendix table 4.** Description of the approach to the model-based analyses of economic evaluations of physical activity mass media campaigns. **Appendix table 5.** Main economic evaluation findings of model-based analyses of physical activity mass media campaigns. **Appendix table 6.** Description of cost items and valuation sources used in the economic evaluations of physical activity mass media campaign. **Appendix table 7.** Quality of economic evaluation of physical activity mass media campaigns according to CHEC-List. **Appendix table 8.** Expanded CHEC-list: Additional questions on the quality of economic evaluations of physical activity mass media campaigns. **Appendix table 9.** Quality of costing studies according to a modified version of CHEC-list. **Appendix table 10.** Intervention costs description

## Data Availability

All the data generated or analysed during this study are included in this published article and its supplementary information files.
