# Supplementary material for: Economic evaluation of physical activity mass media campaigns across the globe: a systematic review
Source: Int J Behav Nutr Phys Act. 2022 Aug 26;19:107. doi: 10.1186/s12966-022-01340-x (PMC9419405; doi:10.1186/s12966-022-01340-x)
Supplement: Supplementary file 1 — Additional file 1: Appendix 1. PRISMA checklists. Appendix 2. Glossary. Appendix 3. Search strategy. Appendix 4. Inclusion and exclusion criteria. Appendix 5. Quality assessment of economic evaluations included in the review using the Extended Consensus on Health Economic Criteria list (CHEC-list). Appendix 6. Quality assessment of costing studies using a modified version of the Consensus Health Economic Criteria List (CHEC-list). Appendix 7. Expanded CHEC-list: Additional questions on the quality of economic evaluations of physical activity mass media campaigns. Appendix 8. GRADE style rating for model-based economic evaluation of physical activity mass media campaigns. Appendix 9. Records excluded at full-text screening and reasons for exclusion. Appendix 10. Implicit thresholds for willingness to pay for each country provided by WHO team. Appendix 11. Application of the GRADE style rating to assess the certainty of each economic model for WHO decision-making. Appendix 12. Additional resources on Agita São Paulo campaign. Appendix table 1. Characteristics of studies included in this review according to study type: model-based analyses and costing studies. Appendix table 2. Mass media campaigns by target population. Appendix table 3. Mass media campaigns by geographical location. Appendix table 4. Description of the approach to the model-based analyses of economic evaluations of physical activity mass media campaigns. Appendix table 5. Main economic evaluation findings of model-based analyses of physical activity mass media campaigns. Appendix table 6. Description of cost items and valuation sources used in the economic evaluations of physical activity mass media campaign. Appendix table 7. Quality of economic evaluation of physical activity mass media campaigns according to CHEC-List. Appendix table 8. Expanded CHEC-list: Additional questions on the quality of economic evaluations of physical activity mass media campaigns. Appendix table 9. Quality of costing stud [file 12966_2022_1340_MOESM1_ESM.docx]

Table of Contents: Appendix material

[Appendix 1. Glossary 2](#_Toc108000842)

[Appendix 2. Search strategy 5](#_Toc108000843)

[Appendix 3. Inclusion and exclusion criteria 12](#_Toc108000844)

[Appendix 4. Quality assessment of economic evaluations included in the review using the Extended Consensus on Health Economic Criteria list (CHEC-list) 14](#_Toc108000845)

[Appendix 5. Quality assessment of costing studies using a modified version of the Consensus Health Economic Criteria List (CHEC-list) 22](#_Toc108000846)

[Appendix 6. Expanded CHEC-list: Additional questions on the quality of economic evaluations of physical activity mass media campaigns 24](#_Toc108000847)

[Appendix 7. GRADE style rating for model-based economic evaluation of physical activity mass media campaigns 25](#_Toc108000848)

[Appendix 8. Records excluded at full-text screening and reasons for exclusion 28](#_Toc108000849)

[Appendix 9. Implicit thresholds for willingness to pay for each country provided by WHO team 30](#_Toc108000850)

[Appendix 10. Application of the GRADE style rating to assess the certainty of each economic model for WHO decision-making 31](#_Toc108000851)

[Appendix 11. Additional resources on Agita São Paulo campaign 42](#_Toc108000852)

[Appendix table 1. Characteristics of studies included in this review according to study type: model-based analyses and costing studies 46](#_Toc108000853)

[Appendix table 2. Mass media campaigns by target population 62](#_Toc108000854)

[Appendix table 3. Mass media campaigns by geographical location 63](#_Toc108000855)

[Appendix table 4. Description of the approach to the model-based analyses of economic evaluations of physical activity mass media campaigns 64](#_Toc108000856)

[Appendix table 5. Main economic evaluation findings of model-based analyses of physical activity mass media campaigns 67](#_Toc108000857)

[Appendix table 6. Description of cost items and valuation sources used in the economic evaluations of physical activity mass media campaign 70](#_Toc108000858)

[Appendix table 7. Quality of economic evaluation of physical activity mass media campaigns according to CHEC-List 71](#_Toc108000859)

[Appendix table 8. Expanded CHEC-list - Additional questions on the quality of economic evaluations of physical activity mass media campaigns 72](#_Toc108000860)

[Appendix table 9. Quality of costing studies according to a modified version of CHEC-list 73](#_Toc108000861)

[Appendix table 10. Intervention costs description 74](#_Toc108000862)

| **Section and Topic** | **Item #** | **Checklist item** | **Reported (Yes/No)** |
| --- | --- | --- | --- |
| **TITLE** | | |  |
| Title | 1 | Identify the report as a systematic review. | Yes |
| **BACKGROUND** | | |  |
| Objectives | 2 | Provide an explicit statement of the main objective(s) or question(s) the review addresses. | Yes |
| **METHODS** | | |  |
| Eligibility criteria | 3 | Specify the inclusion and exclusion criteria for the review. | Yes |
| Information sources | 4 | Specify the information sources (e.g. databases, registers) used to identify studies and the date when each was last searched. | Yes |
| Risk of bias | 5 | Specify the methods used to assess risk of bias in the included studies. | Yes |
| Synthesis of results | 6 | Specify the methods used to present and synthesise results. | Yes |
| **RESULTS** | | |  |
| Included studies | 7 | Give the total number of included studies and participants and summarise relevant characteristics of studies. | Yes |
| Synthesis of results | 8 | Present results for main outcomes, preferably indicating the number of included studies and participants for each. If meta-analysis was done, report the summary estimate and confidence/credible interval. If comparing groups, indicate the direction of the effect (i.e. which group is favoured). | Yes |
| **DISCUSSION** | | |  |
| Limitations of evidence | 9 | Provide a brief summary of the limitations of the evidence included in the review (e.g. study risk of bias, inconsistency and imprecision). | Yes |
| Interpretation | 10 | Provide a general interpretation of the results and important implications. | Yes |
| **OTHER** | | |  |
| Funding | 11 | Specify the primary source of funding for the review. | Yes |
| Registration | 12 | Provide the register name and registration number. | Yes |

# Appendix 1. PRISMA checklists

**Abstract checklist**

**PRISMA Checklist**

| **Section and Topic** | **Item #** | **Checklist item** | **Location where item is reported** |
| --- | --- | --- | --- |
| **TITLE** | | |  |
| Title | 1 | Identify the report as a systematic review. | Page 1 |
| **ABSTRACT** | | |  |
| Abstract | 2 | See the PRISMA 2020 for Abstracts checklist. | Page 4 |
| **INTRODUCTION** | | |  |
| Rationale | 3 | Describe the rationale for the review in the context of existing knowledge. | Page 6 |
| Objectives | 4 | Provide an explicit statement of the objective(s) or question(s) the review addresses. | Page 6-7 |
| **METHODS** | | |  |
| Eligibility criteria | 5 | Specify the inclusion and exclusion criteria for the review and how studies were grouped for the syntheses. | Page 7-8 |
| Information sources | 6 | Specify all databases, registers, websites, organisations, reference lists and other sources searched or consulted to identify studies. Specify the date when each source was last searched or consulted. | Page 7 |
| Search strategy | 7 | Present the full search strategies for all databases, registers and websites, including any filters and limits used. | Appendix 2 |
| Selection process | 8 | Specify the methods used to decide whether a study met the inclusion criteria of the review, including how many reviewers screened each record and each report retrieved, whether they worked independently, and if applicable, details of automation tools used in the process. | Page 8-9 |
| Data collection process | 9 | Specify the methods used to collect data from reports, including how many reviewers collected data from each report, whether they worked independently, any processes for obtaining or confirming data from study investigators, and if applicable, details of automation tools used in the process. | Page 8-9 |
| Data items | 10a | List and define all outcomes for which data were sought. Specify whether all results that were compatible with each outcome domain in each study were sought (e.g. for all measures, time points, analyses), and if not, the methods used to decide which results to collect. | Page 8, Appendix 3 |
|  | 10b | List and define all other variables for which data were sought (e.g. participant and intervention characteristics, funding sources). Describe any assumptions made about any missing or unclear information. | Page 8 |
| Study risk of bias assessment | 11 | Specify the methods used to assess risk of bias in the included studies, including details of the tool(s) used, how many reviewers assessed each study and whether they worked independently, and if applicable, details of automation tools used in the process. | Page 9-10 Appendix 4-5 |
| Effect measures | 12 | Specify for each outcome the effect measure(s) (e.g. risk ratio, mean difference) used in the synthesis or presentation of results. | N/A |
| Synthesis methods | 13a | Describe the processes used to decide which studies were eligible for each synthesis (e.g. tabulating the study intervention characteristics and comparing against the planned groups for each synthesis (item #5)). | Page 8-9 |
|  | 13b | Describe any methods required to prepare the data for presentation or synthesis, such as handling of missing summary statistics, or data conversions. | Page 10 |
|  | 13c | Describe any methods used to tabulate or visually display results of individual studies and syntheses. | Page 10 |
|  | 13d | Describe any methods used to synthesize results and provide a rationale for the choice(s). If meta-analysis was performed, describe the model(s), method(s) to identify the presence and extent of statistical heterogeneity, and software package(s) used. | Page 10 |
|  | 13e | Describe any methods used to explore possible causes of heterogeneity among study results (e.g. subgroup analysis, meta-regression). | N/A |
|  | 13f | Describe any sensitivity analyses conducted to assess robustness of the synthesized results. | N/A |
| Reporting bias assessment | 14 | Describe any methods used to assess risk of bias due to missing results in a synthesis (arising from reporting biases). | N/A |
| Certainty assessment | 15 | Describe any methods used to assess certainty (or confidence) in the body of evidence for an outcome. | Page 9-10, Appendix 7 |
| **RESULTS** | | |  |
| Study selection | 16a | Describe the results of the search and selection process, from the number of records identified in the search to the number of studies included in the review, ideally using a flow diagram. | Page 11, Appendix figure 1 |
|  | 16b | Cite studies that might appear to meet the inclusion criteria, but which were excluded, and explain why they were excluded. | Page 11 &  Appendix 8 |
| Study characteristics | 17 | Cite each included study and present its characteristics. | Appendix table 1-3 |
| Risk of bias in studies | 18 | Present assessments of risk of bias for each included study. | Page 13-14,  Appendix 7,  Appendix 10 |
| Results of individual studies | 19 | For all outcomes, present, for each study: (a) summary statistics for each group (where appropriate) and (b) an effect estimate and its precision (e.g. confidence/credible interval), ideally using structured tables or plots. | Page 11-12 Table 1-4  Appendix table 5-6 |
| Results of syntheses | 20a | For each synthesis, briefly summarise the characteristics and risk of bias among contributing studies. | Page 13-15, Appendix table 7-8 |
|  | 20b | Present results of all statistical syntheses conducted. If meta-analysis was done, present for each the summary estimate and its precision (e.g. confidence/credible interval) and measures of statistical heterogeneity. If comparing groups, describe the direction of the effect. | N/A |
|  | 20c | Present results of all investigations of possible causes of heterogeneity among study results. | Page 12, Appendix table 4 |
|  | 20d | Present results of all sensitivity analyses conducted to assess the robustness of the synthesized results. | N/A |
| Reporting biases | 21 | Present assessments of risk of bias due to missing results (arising from reporting biases) for each synthesis assessed. | Page 13 |
| Certainty of evidence | 22 | Present assessments of certainty (or confidence) in the body of evidence for each outcome assessed. | Page 13-14 |
| **DISCUSSION** | | |  |
| Discussion | 23a | Provide a general interpretation of the results in the context of other evidence. | Page 15-17 |
|  | 23b | Discuss any limitations of the evidence included in the review. | Page 18 |
|  | 23c | Discuss any limitations of the review processes used. | Page 18 |
|  | 23d | Discuss implications of the results for practice, policy, and future research. | Page 19 |
| **OTHER INFORMATION** | | |  |
| Registration and protocol | 24a | Provide registration information for the review, including register name and registration number, or state that the review was not registered. | Page 7, reference 22 |
|  | 24b | Indicate where the review protocol can be accessed, or state that a protocol was not prepared. | Page 7 |
|  | 24c | Describe and explain any amendments to information provided at registration or in the protocol. | N/A |
| Support | 25 | Describe sources of financial or non-financial support for the review, and the role of the funders or sponsors in the review. | Page 2, Page 20 |
| Competing interests | 26 | Declare any competing interests of review authors. | Page 2 |
| Availability of data, code and other materials | 27 | Report which of the following are publicly available and where they can be found: template data collection forms; data extracted from included studies; data used for all analyses; analytic code; any other materials used in the review. | No |

*From:*  Page MJ, McKenzie JE, Bossuyt PM, Boutron I, Hoffmann TC, Mulrow CD, et al. The PRISMA 2020 statement: an updated guideline for reporting systematic reviews. BMJ 2021;372:n71. doi: 10.1136/bmj.n71

For more information, visit: <http://www.prisma-statement.org/>

# Appendix 2. Glossary

Adapted from YHEC glossary: Glossary – Health economic terms (2016). York; York Health Economics Consortium; 2016. <https://yhec.co.uk/resources/glossary/>

**Cost-effectiveness threshold**: represents the opportunity cost of health foregone when deciding to reimburse/fund a new technology. The underlying economic principle is that given a fixed budget a decision to reimburse a new healthcare intervention implies that funds will not be available to fund some other intervention which would deliver health benefits, and that these health benefits would be obtained at the ‘marginal’ rate represented by the threshold. The threshold often represents a specific cost per additional QALY value (eg £50,000 per QALY), so if the ICER of an intervention is less than this value it is likely to be considered cost-effective.

**Deterministic sensitivity analysis**: allows a reviewer to assess the impact that changes in a certain input (parameter) will have on the output results of an economic evaluation – this may be referred to as assessing the robustness of the result to that parameter. The parameter of interest should be varied between plausible extremes, preferable justified by review of available evidence. This is the simplest form of sensitivity analysis since only one parameter is changed at one time, and correlations between parameters is not taken into account.

**Discounting:** Economic evaluations refer to a choice to be made between alternative interventions at a specific point in time, however the costs and health outcomes associated with each intervention occur at different points in time, present or future. Costs and health outcomes that are predicted to occur in the future are usually valued less than present costs, and so it is recommended that they be discounted in analysis. This is usually achieved by expressing the results as series (streams) of health outcomes and costs over time, applying a discounting factor to each value in the series and then aggregating to give a ‘present value’ of each stream. The discount factor increases over time, based on an underlying discount rate. If we apply a discount rate of 3.5% per year for costs than £100 spent in year 2 would have a ‘present’ value of £96.50 in year 1.

**Incremental cost-effectiveness ratio (ICER):** represents the economic value of an intervention, compared with an alternative. An ICER is calculated by dividing the difference in total costs (incremental cost) by the difference in the chosen measure of health outcome or effect (incremental effect) to provide a ratio of ‘extra cost per extra unit of health effect’ – for the more expensive therapy vs the alternative. In the UK the QALY is most frequently used as the measure of health effect, enabling ICERs to be compared across disease areas. In decision-making ICERs are most useful when the new intervention is more costly but generates improved health effect. ICERs reported by economic evaluations are compared with a pre-determined threshold (see cost-effectiveness threshold) in order to decide whether choosing the new intervention is an efficient use of resources.

**Opportunity cost:** The opportunity cost of an intervention is what is foregone as a consequence of adopting a new intervention. In a fixed budget health care system where increased costs will displace other health care services already provided, the opportunity cost is measured as the health lost as a result of the displacement of activities to fund the selected intervention.

**Perspective:** The point of view employed when deciding which types of costs and health benefits are to be included in an economic evaluation. Typical perspectives are: patient, hospital/clinic, healthcare system or society.

**Probabilistic sensitivity analysis (PSA):** is a technique used in economic modelling that allows the modeller to quantify the level of confidence in the output of the analysis, in relation to uncertainty in the model inputs. There is usually uncertainty associated with input parameter values of an economic model, which may have been derived from clinical trials, observational studies or in some cases expert opinion. In the base case analysis, the point estimate of each input parameter value is used. In the probabilistic analysis, these parameters are represented as distributions around the point estimate, which can be summarised using a few parameters (such as mean and standard deviation for a normal distribution). Different distributions are generally appropriate for different types of variable, where possible backed up by supporting evidence from source studies. For example measures of effect such as hazard ratios or relative risk reductions may be represented by a normal distribution, and survival curves by a Weibull distribution. In a PSA, a set of input parameter values is drawn by random sampling from each distribution, and the model is ‘run’ to generate outputs (cost and health outcome), which are stored. This is repeated many times (typically 1,000 to 10,000), resulting in a distribution of outputs that can be graphed on the cost-effectiveness plane, and analysed. A key output of a PSA is the proportion of results that fall favourably (i.e. considered cost-effective) in relation to a given cost-effectiveness threshold. This may be represented using a cost-effectiveness acceptability curve.

**Trial-based economic evaluation**: economic evaluation conducted using intervention evidence directly from single source data, where the intervention or program of interest is investigated using any evaluation design.

**Quality-adjusted life year (QALY):** is a summary outcome measure used to quantify the effectiveness of a particular intervention. Since the benefits of different interventions are multi-dimensional, QALYs have been designed to combine the impact of gains in quality of life and in quantity of life (ie life expectancy) associated with an intervention. In this case it is the incremental (ie differences between 2 or more alternatives) QALYs, compared with the incremental costs, that provides the measure of economic value. If a wide range of aspects (domains) of quality of life is included in the quality component, the resulting QALYs should be comparable across disease areas, which is valuable for decision-making. More specifically, QALYs are based on utilities, which are valuations of health-related quality of life measured on a scale where full health is valued as 1 and death as 0. These valuations are the multiplied by the duration of time (in years) that a subject spends in a health state with that particular utility score, and aggregate QALYs are then summed over the time horizon of the analysis (often lifetime).

**Time horizon:** the duration over which health outcomes and costs are calculated. The choice of time horizon is an important decision for economic modelling, and depends on the nature of the disease and intervention under consideration and the purpose of the analysis. Longer time horizons are applicable to chronic conditions associated with on-going medical management, rather than a cure. A shorter time horizon may be appropriate for some acute conditions, for which long-term consequences are less important. The same time horizon should be used for both costs and health outcomes.

**Univariate/one way sensitivity analysis:**  allows a reviewer to assess the impact that changes in a certain input (parameter) will have on the output results of an economic evaluation (most frequently those based on a model) – this may be referred to as assessing the robustness of the result to that parameter. The parameter of interest should be varied between plausible extremes, preferable justified by review of available evidence. This is the simplest form of sensitivity analysis since only one parameter is changed at one time, and correlations between parameters is not taken into account. Tornado diagrams are often used to summarise univariate sensitivity analyses testing a set of input variables in turn.

# Appendix 3. Search strategy

**Embase (via Ovid)**

1. economics/
2. exp "costs and cost analysis"/
3. cost benefit analysis.tw.
4. (economic* adj evaluation*).tw.
5. (economic adj appraisal).tw
6. (economic adj analysis).tw
7. (cost* adj2 analysis).tw
8. (cost* adj2 effect*).tw.
9. (cost* adj2 benefit).tw.
10. (cost* adj2 utility).tw.
11. (cost adj2 effic*).tw.
12. (economic* adj2 analysis).tw.
13. quality-adjusted life years/
14. quality adjusted life year*.tw
15. quality-adjsuted life year*.tw.
16. (value adj2 money).ti,ab.
17. QALY*.tw.
18. disability-adjusted life year*.tw
19. disability adjusted life year*.tw
20. DALY.tw
21. ICER.tw
22. OR 1/21
23. exp mass media/ ~~or~~
24. motion pictures/
25. radio/
26. television/
27. exp audiovisual aids/
28. (mass adj1 campaign*).ti,ab
29. (mass adj1 media).ti,ab
30. television.ti,ab
31. radio.ti,ab
32. newspaper.ti,ab
33. magazine.ti,ab
34. media campaign*.ti,ab
35. public adj1 (education or communication*).ti,ab
36. health adj1 education.ti,ab
37. health communication*.ti,ab
38. social adj1 (media or marketing).ti,ab
39. marketing.ti,ab
40. campaign*.ti,ab
41. brand*.ti,ab.
42. diffusion adj1 information.ti,ab
43. advertising.ti,ab
44. advertisement*.ti,ab
45. 23 / 44 OR
46. physical activity.ti,ab
47. exercise/
48. exercise.ti,ab
49. sport*.ti,ab
50. inactivity.ti,ab
51. walk*.ti,ab
52. swim*.ti,ab.
53. (physical* adj2 (fit* or train* or activ* or endur* or exer*)).ti,ab.
54. (exercis* adj2 (train* or physical* or activ*)).ti,ab.
55. active adj1 (living or transport or commut*).ti,ab
56. fitness.ti,ab
57. bike.ti,ab
58. bicycle.ti,ab
59. cycling.ti,ab
60. bicycling.ti,ab
61. OR 46/60
62. 22 AND 45 AND 61

*Search date: 3 June 2021*

*Results: 673*

**Medline (Ovid)**

1. Economics/
2. exp "Costs and Cost Analysis"/
3. cost benefit analysis.tw.
4. (economic* adj evaluation*).tw.
5. Economic adj appraisal.tw
6. Economic adj analysis.tw
7. (cost* adj2 analysis).tw
8. (cost* adj2 effect*).tw.
9. (cost* adj2 benefit).tw.
10. (cost* adj2 utility).tw.
11. (cost adj2 effic*).tw.
12. (economic* adj2 analysis).tw.
13. quality-adjusted life years/
14. quality adjusted life year*.tw
15. quality-adjsuted life year*.tw.
16. (value adj2 money).ti,ab.
17. QALY*.tw.
18. Disability-adjusted life year*.tw
19. Disability adjusted life year*.tw
20. DALY.tw
21. ICER.tw
22. 1 / 22 or
23. exp mass media/ or
24. motion pictures/
25. radio/
26. television/
27. exp Audiovisual Aids/
28. (Mass adj1 campaign*).ti,ab
29. (mass adj1 media).ti,ab
30. television.ti,ab
31. radio.ti,ab
32. newspaper.ti,ab
33. magazine.ti,ab
34. media campaign*.ti,ab
35. public adj1 (education or communication*).ti,ab
36. health adj1 education.ti,ab
37. health communication*.ti,ab
38. social adj1 (media or marketing).ti,ab
39. marketing.ti,ab
40. campaign*.ti,ab
41. brand*.ti,ab.
42. diffusion adj1 information.ti,ab
43. advertising.ti,ab
44. Advertisement*.ti,ab
45. 24 / 45 OR
46. physical activity.ti,ab
47. exercise/
48. exercise.ti,ab
49. sport*.ti,ab
50. inactivity.ti,ab
51. walk*.ti,ab
52. swim*.ti,ab.
53. (physical* adj2 (fit* or train* or activ* or endur* or exer*)).ti,ab.
54. (exercis* adj2 (train* or physical* or activ*)).ti,ab.
55. Active adj1 (living or transport or commut*).ti.ab
56. Fitness.ti,ab
57. Bike.ti,ab
58. Bicycle.ti,ab
59. Cycling.ti,ab
60. Bicycling.ti,ab
61. 42 / 56 OR
62. 23 AND 46 AND 62

*Search date: 3 June 2021*

*Result: 363*

**NHS EED**

<https://www.crd.york.ac.uk/CRDWeb/>

1. MeSH Descriptor mass media/
2. MeSH Descriptor motion pictures/
3. MeSH Descriptor radio/
4. Television (any field)
5. MeSH Descriptor Audiovisual Aids/
6. Mass adj1 campaign* (any field)
7. mass adj1 media (any field)
8. television (any field)
9. radio (any field)
10. newspaper (any field)
11. magazine (any field)
12. media campaign*(any field)
13. public adj1 (education or communication*(any field)
14. health adj1 education (any field)
15. health communication*(any field)
16. social adj1 (media or marketing) (any field)
17. marketing (any field)
18. campaign*(any field)
19. brand* (any field)
20. diffusion adj1 information (any field)
21. advertising (any field)
22. Advertisement* (any field)
23. 23 / 44 OR
24. physical activity (any field)
25. MESH DESCRIPTOR exercise
26. Exercise (any field)
27. sport* (any field)
28. inactivity (any field)
29. walk* (any field)
30. swim* (any field)
31. (physical* adj2 (fit* or train* or activ* or endur* or exer*)) (any field)
32. (exercis* adj2 (train* or physical* or activ*)) (any field)
33. Active adj1 (living or transport or commut*)(any field)
34. Fitness (any field)
35. Bike (any field)
36. Bicycle (any field)
37. Cycling (any field)
38. Bicycling (any field)
39. OR 46/60
40. 23 AND 39

*Search date: 4 June 2021*

*Results: 71*

**Health Technology Assessment (HTA) database**

1. MeSH Descriptor mass media/
2. MeSH motion pictures/
3. MeSH Descriptor radio/
4. Television (any field)
5. MeSH Descriptor Audiovisual Aids/
6. Mass adj1 campaign* (any field)
7. mass adj1 media (any field)
8. television (any field)
9. radio (any field)
10. newspaper (any field)
11. magazine (any field)
12. media campaign*(any field)
13. public adj1 (education or communication*)(any field)
14. health adj1 education (any field)
15. health communication*(any field)
16. social adj1 (media or marketing) (any field)
17. marketing (any field)
18. campaign*(any field)
19. brand* (any field)
20. diffusion adj1 information (any field)
21. advertising (any field)
22. Advertisement* (any field)
23. 23 / 44 OR
24. physical activity (any field)
25. MESH DESCRIPTOR exercise
26. Exercise (any field)
27. sport* (any field)
28. inactivity (any field)
29. walk* (any field)
30. swim* (any field)
31. (physical* adj2 (fit* or train* or activ* or endur* or exer*)) (any field)
32. (exercis* adj2 (train* or physical* or activ*)) (any field)
33. Active adj1 (living or transport or commut*)(any field)
34. Fitness (any field)
35. Bike (any field)
36. Bicycle (any field)
37. Cycling (any field)
38. Bicycling (any field)
39. OR 46/60
40. 23 AND 39

*Search date: 4 June 2021*

*Results: 24*

**Research Papers in Economics (RePEc)**

<https://econpapers.repec.org/scripts/search.pf>

(physical activity OR exercise OR fitness OR walk OR sport) AND (campaign OR mass media OR marketing) AND (economic* OR cost* OR value OR QALY OR ICER OR DALY)

“journal articles”

*Search date: 07 June 2021*

*Results: 59*

**EconLit (via Ebsco)**

1. TI walk* OR AB walk*
2. TI Sport* OR AB Sport*
3. TI inactivity OR AB inactivity
4. TI swim* OR AB swim*
5. TI physical activity OR AB physical activity
6. TI exercise OR AB exercise
7. TI fitness OR AB fitness
8. TI physical exercise OR AB physical exercise
9. TX “Physical activity”
10. TI bike or TI bicycle or TI cycling or TI bicycling
11. AB bike or AB bicycle or AB cycling or AB bicycling
12. 1/11 OR
13. TI ( mass media or campaign* ) OR AB ( mass media or campaign* )
14. TI marketing OR AB marketing
15. TI brand* OR AB brand*
16. TI advert* OR AB advert*
17. TI magazine OR AB magazine
18. TI newspaper OR AB newspaper
19. TI radio OR AB radio
20. TI television OR AB television
21. TI health education OR AB health education
22. TI health communication* OR AB health communication*
23. TI public education OR AB public education
24. TX Communications Media
25. 13/24 OR
26. 12 AND 25

*Search date: 4 June 2021*

*Results: 898*

# Appendix 4. Inclusion and exclusion criteria

| **Inclusion criteria** | | **Exclusion criteria** |
| --- | --- | --- |
| **Type of study** | - Full (cost-effectiveness, cost-utility, or cost-benefit analysis) and partial (cost or cost-consequences analysis) economic evaluation - Trial-based or modelled economic evaluations that provide comparative information on the costs and health outcomes of at least one public education campaign intervention versus a control intervention or period | - Systematic reviews of economic evaluations |
| **Publication / language** | - Peer-reviewed manuscripts and policy relevant reports from trustworthy organisations - No restriction on country income status |  |
| **Population** | - All age groups - Campaigns targeting the general population with no age limit - Campaigns targeting clinical populations/ people with existing conditions (e.g. diabetes or musculoskeletal disorders) will be included provided that physical activity (PA) is the main focus of the campaign and that it meets the other criteria |  |
| **Intervention** | - Studies reporting population- or large-group focused campaigns, targeting a whole population or population subgroup (could include social media campaigns, but must be at scale a population target or segment) - Campaigns using mass media or public communications or social marketing or social media or other communication channels to persuade, inform, refer or motivate a population to think about or initiate or increase physical activity - Obesity campaigns or NCD campaigns where PA was a clearly defined sub-component and where PA measures or antecedent of PA are reported [such as knowledge of PA, attitudes to PA, PA efficacy or intention] - Include campaigns targeting park use or trails or similar if they have clear elements of mass communications campaign to support those environmental interventions to promote PA | - Exclude campaigns in single settings [such as a physical activity campaign in a school or a few schools or a workplace], but preserving a PA campaign in a whole community or region or City-level campaign |
| **Comparator** | - Control period, “usual care” (i.e. no change in usual activities / business as usual) or no intervention |  |
| **Health Outcomes** | - Main outcomes**:**    - mass media campaign awareness, campaign recognition, or campaign message understanding   - antecedents of PA such as knowledge, attitudes, efficacy or intention   - PA behaviours - Additional outcomes**:** quality of life, health-related utility |  |
| **Health Economic Outcomes** | - Main outcomes: incremental cost effectiveness ratio (ICER) expressed as the incremental cost per change in physical activity or the incremental cost per quality-adjusted life year (QALY) gained, or disability adjusted-life year (DALY) avoided - Additional outcomes**:** Costs (total costs, intervention costs, health service utilisation costs, community services costs, out of pocket costs) |  |

DALY: disability adjusted-life year, ICER: incremental cost-effectiveness ratio, NCD: non-communicable disease, PA: physical activity, QALY: quality-adjusted life year

# Appendix 5. Quality assessment of economic evaluations included in the review using the Extended Consensus on Health Economic Criteria list (CHEC-list)

**CHEC-Extended - A tool for the quality assessment of economic evaluations of healthcare interventions**

The text in black are the assessment instructions for the CHEC-list.

The text in blue is from the Appendix to the publication: Odnoletkova I, Goderis G, Pil L, Nobels F, Aertgeerts B, et al. (2014) Cost-Effectiveness of Therapeutic Education to Prevent the Development and Progression of Type 2 Diabetes: Systematic Review. J Diabetes Metab 5: 438.

The text in red are adaptations made by the review authors.

|  | **CHEC-Extended question** | **Guidelines to support the value judgement** | **Specific guidelines for the models** |
| --- | --- | --- | --- |
| 1. | Is the study population clearly described? | The relevant clinical characteristics, entry and eligibility criteria, as well as drop-out during follow-up should be stated explicitly.  The study population should be described in terms of geography, patient characteristics such as age, sex, ethnicity (Higgins et al.), co-morbid conditions, and disease stage/previous treatments, each of which should be appropriate to the decision problem. (Caro et al.)  The patient population to which the economic evaluation applies should be consistent with the patient population defined in the clinical part of the study. (Cleemput et al.) |  |
| 2. | Are competing alternatives clearly described? | A detailed description should be given of the competing interventions. This should encompass a clear and specific statement of the primary objective of each alternative, as well as relevant factors, such as intensity, duration, and frequency.  The competing alternatives should be clearly defined in terms of frequency, component services, dose or intensity, duration, and any variations required for target subgroups It should be mentioned whether people involved in delivery of the intervention need to be trained (adapted from Higgins et al. and Caro et al.). The choice of the comparator(s) should always be justified. The comparator should be the most cost-effective alternative intervention currently available. |  |
| 3. | Is a well-defined research question posed in answerable form? | A research question must identify clearly the alternatives being compared and the population for which the comparison is made.  The research question should specify the type of population (participants), type of interventions (and comparisons), the type of outcomes and the type of study that was performed (Higgins et al.) |  |
| 4. | Is the economic study design appropriate to the stated objective? | An appropriate economic study design is a full economic evaluation (comparison of costs and effects of 2 or more interventions) based on primary research (cohort, case-control, randomised controlled trial).  Trial based economic evaluations are appropriate when the available data are sufficient to allow a full assessment of the cost-effectiveness or cost-utility of an Intervention. That means: the effect of the treatment cannot go beyond the duration of the trial;the intermediate outcome parameters do not have potential impact on the clinical endpoints such as long-term mortality, quality adjusted life years gained, or life years gained on a long term. (Adapted from Cleemput et al.) | Modelling should be applied if the available data are insufficient to allow a full assessment of the cost-effectiveness or cost-utility of an intervention. That means:  -the effect of the treatment might go beyond the duration of the trial;  -the intermediate outcome parameters have potential impact on the clinical endpoints such as long-term mortality, quality adjusted life years gained, or life years gained on a long term.  Modelling is also appropriate:   - to simulate the real-life application of an intervention based on the data available from clinical trials. This can be done e.g. by adjusting for differences in baseline risk between the trial population and the real-world target population and adjusting for protocol-driven costs or events. - to account for possible externalities associated with the disease or treatment (e.g. transmission of infections, bacterial resistance...) that were not part of the original study design and therefore not captured during clinical trial. - to compare the intervention with the relevant comparator if the respective interventions have never been directly compared in a clinical trial.   (adapted from Cleemput et al.) |
| 5 | Are the structural assumptions and the validation methods of the model properly reported? |  | For models, the following information should be presented:  Structure:  -the structural hypotheses/ assumptions  -the uncertainty around these assumptions  -sources of information for these assumptions (systematic reviews preferred).(adapted from Cleemput et al.)  Validity:  Methods to verify the model’s  - structure (face) validity,  - performance (technical/internal) validity  - outcomes validity should be discussed. (Adapted from Weinstein et al. and Caro et al.) |
| 6. | Is the chosen time horizon appropriate in order to include relevant costs and consequences? | The period of analysis of the study is the time horizon. This time horizon should always be equal for costs and outcomes if these are combined in a ratio. The time span should be long enough to include all relevant costs and outcomes relating the intervention. Ideally, the follow-up period should be extended till the situation is stabilised with reference to costs and effects.  The chosen time horizon should be long enough to capture relevant differences in outcomes across strategies. (Caro et al.) Treatments of chronic diseases mostly have consequences over a patient’s lifetime. (Cleemput et al.) |  |
| 7. | Is the actual perspective chosen appropriate? | ‘Perspective’ indicates from which point of view an economic evaluation study is performed. If the study is performed from a societal perspective tick ‘yes’, as all relevant costs and consequences of an interventions and disease are taken into account, if possible. Other narrower perspectives will only include certain components. The authors should motivate why a narrower perspective is valid.  The perspective of the analysis should be stated and defined. Analyses which take a perspective narrower than the societal perspective should report and justify the included and excluded outcomes. (Caro et al.) |  |
| 8. | Are all important and relevant costs for each alternative identified? | A full identification of all important and relevant costs should be given in relation to the perspective and the research question.  The identification of costs should be consistent with the chosen perspective and the assessed area of disease and treatments. For the perspective of the health care payer, at least all direct health care costs associated with or influenced by the competing alternatives must be included. For the societal perspective, also direct and indirect costs outside the health care sector, such as productivity loss, should be included.(adapted from Cleemput et al.) |  |
| 9. | Are all costs measured appropriately in physical units? | The costs should be measured appropriately in physical units. The instrument by which the costs are measured should be valid and clearly stated (e.g. interview, questionnaire, cost-diary).  Validated sources should be used for the measurement of the resource –and material use, such as observations from clinical trials, prospective observational studies, databases and patient charts, or derived from literature. If derived from literature or studies from other countries, resource use estimates should be validated for the local context. (Cleemput et al.) |  |
| 10. | Are costs valued appropriately? | The sources of valuation should be clearly stated for each cost price of every volume parameter and their reference year. The main cost should be calculated based on depleted sources, no tariffs should be used.  All costs should be expressed in values by using prices of a particular year indicated in the study. (adapted from Cleemput et al.) Adjustment for inflation should be based on the Consumer Price Index (CPI) or its health-care component. The method of choice for making adjustments across countries is to use purchasing power parity. However, a simple currency conversion would be appropriate if there is an international market for an input at a fixed price.(Weinstein et al.) |  |
| 11. | Are all important and relevant outcomes for each alternative identified? | A full identification of all important and relevant outcomes should be given in relation to the perspective and the research question.  Outcomes in economic evaluations should be expressed in terms of final endpoints instead of intermediary outcomes, i.e. in life years gained, in quality adjusted life years (QALYs) gained (Cleemput et al.), or in disability-adjusted life-years. (Caro et al.) Intermediate outcomes (useful for outcomes validation) can of course be reported and may include number of events, incidence of disease, mortality, adverse events ... (Caro et al.) Differences in outcomes between subgroups should be stated if appropriate. (Cleemput et al.) | Systematic methods for identifying the outcomes should be used. |
| 12. | Are all outcomes measured appropriately? | The outcome measurement should result from the outcome identification and this should be straightforward (e.g. if mortality is a main outcome measure this should be taken into account in the analysis). The instrument by which the outcomes are measured should be valid and clearly stated.  Quality of the clinical evidence from which the differences in health outcomes were derived, should be critically appraised. QALYs should be derived from the utility weights obtained from the self-reported health status of the study participants. Validated generic health-related quality-of-life instruments should be used. |  |
| 13. | Are outcomes valued appropriately? | The method of outcome valuation should be clearly stated. Examples of valuation methods are Discrete Choice Experiments (e.g. Conjoint analysis, Contingent valuation), Direct utility assessment (VAS, TTO, SG, etc.), Indirect utility assessment (HUI, EQ-5D, QWB, etc.), Person trade off, etc.  The generic health-related quality of life instruments used should correspond with pre-specified scoring systems based on “forced-choice” methods (standard gamble, time trade-off) reflecting the preferences of the general public. (Weinstein et al.)Only if measured with the same instrument and in a similar patient population are the values comparable and can they be used in one and the same economic evaluation. If the primary data are not available but only health-related quality-of-life results from trials from another country are used, index values from that country should be used for consistency. Adjustment for baseline (age-and gender-specific) health-related quality of life is required in estimating the incremental utility of an intervention. (Cleemput et al.)Life expectancy should be estimated by using national life tables based on all-cause mortality (Weinstein et al. and Cleemput et al.) |  |
| 14. | Is an incremental analysis of costs and outcomes of alternatives performed? | An incremental analysis should examine the additional costs from one intervention over another, compared to the additional outcomes that it delivers. The incremental costs-effectiveness ratio is obtained by dividing the costs differences (C2-C1) by the outcome differences (O2-O1) for the alternatives.  The difference in the relevant health outcomes should be compared to the difference in all relevant costs associated with the alternative treatments (i.e. not only the additional costs of the intervention). Consistency in the perspective and the time horizon of the clinical and the economic outcomes should thereby be pursued. Incremental cost-effectiveness ratios should only be presented if the treatment is NOT dominant (lower costs and better effectiveness) or dominated (higher costs and lower effectiveness). (Cleemput et al.) |  |
| 15. | Are all future costs and outcomes discounted appropriately? | Discounting is done appropriately if all costs and outcomes are converted to one single year, based on a motivated discount rate.  The method for discounting costs and health effects to present value should be stated and justified. (Weinstein et al.) |  |
| 16. | Are all important variables, whose values are uncertain, appropriately subjected to sensitivity analysis? | All variables in the analysis are potential candidates for the sensitivity analysis. Only variables that are certain or which have a minimal impact on the study results (based on the preliminary analysis) can be excluded from the sensitivity analysis. Furthermore, a justification should be given over the range of the variables used in the sensitivity analysis.  For all economic evaluations, uncertainty should be analysed using appropriate statistical techniques.  For within-trial economic evaluations:   - The sample uncertainty should be presented through deterministic sensitivity analysis methods (point estimate and range). - Methodological uncertainty coming from the analytical methods chosen such as the discount rate/ missed data imputation etc. should be handled by one-way sensitivity analyses.   The incremental costs and incremental outcomes should be presented with the 95% confidence or credibility interval.  (Adapted from Cleemput et al.)  For all economic evaluations:  To assess the sensitivity of the results to the discount rate applied, different scenarios should be presented. For all analyses of data, methods to handle missing data should be described. It is recommended to show the most important contributors to the uncertainty of the estimated incremental cost-effectiveness/cost-utility ratio (e.g. by means of a Tornado diagram).  The cost-effectiveness plane, with the results of the uncertainty analysis (such as Monte Carlo simulations or bootstrapping), should always be presented. In addition, if simulations are spread over different quadrants of the cost-effectiveness plane, the percentage of simulations in each quadrant should be reported. (adapted from Cleemput et al.)  A cost-effectiveness plane should be displayed. The acceptability curve should be presented to show the probability that the treatment is cost-effective, given varying theoretical threshold values for the cost-effectiveness ratio.(Cleemput et al.) | For models:   - The parameter uncertainty should be tested through probabilistic sensitivity analyses, e.g. by means of Monte Carlo simulations. Beta distributions are a natural match for binomial data; gamma or log normal for right skew parameters; log normal for relative risks or hazard ratios; logistic for odds ratios) (Caro et al.) - The structural uncertainty should be tested through presenting different scenarios to show the impact of different extrapolation approaches on the results.   Possible scenarios:  1)the treatment effect disappears immediately in the extrapolated phase (stop-and-drop approach)  2)the incremental treatment effect stays the same as during the observed phase  3)the initial treatment effect fades out in the long term. |
| 17. | Do the conclusions follow from the data reported? | Do the authors interpret their results cautiously and are their conclusions justified by the data.  Do the authors critically discuss the quality of health economic evidence considering the study limitations and uncertainties? |  |
| 18. | Does the study discuss the generalizability of the results to other settings and patient/client groups? | This can be done by being explicit about the viewpoint of analysis and by indicating how particular costs and outcomes vary by location, setting, patient population, care provider, etc.  Generalisability refers to applicability of the results to other populations (e.g. non-trial populations with different baseline risk). Transferability refers to the applicability of the results from other countries. These two aspects should be assessed separately. (Cleemput et al.) |  |
| 19. | Does the article indicate that there is no potential conflict of interest of study researcher(s) and funder(s)? | If an external agency finances the study, a statement should explicitly be given about who finances the study to guarantee transparency in the relationship between the sponsor and the researcher. Whenever a potential conflict of interest is possible a declaration should be given of ‘competing interest’.  No value judgment implied. |  |
| 20. | Are ethical and distributional issues discussed appropriately? | Does the article notes ethical aspects and elaborates on the characteristics of the population experiencing the disease or the intervention (young, old, poor, wealthy) and how this may have distributional implications.  The morally relevant issues and moral conflicts related to implementing or not implementing the technology have to be synthesized and reported. This includes potential impact on the traditional values such as human equality, autonomy, dignity, the principles of solidarity and justice etc. Summarizing the benefits and harms of introducing/ refraining from the technology for different groups of stakeholders, such as patients, families, care providers, society etc. might be appropriate. The ethical analysis should allow a judgment on their transferability. (Adapted from The HTA Core Model) |  |

# Appendix 6. Quality assessment of costing studies using a modified version of the Consensus Health Economic Criteria List (CHEC-list)

|  | **Modified CHEC-list** | **Guidelines to support the value judgement** |
| --- | --- | --- |
| 1. | Is the study population clearly described? | The study population should be described in terms of geography, patient characteristics such as age, sex, ethnicity (Higgins et al.), co-morbid conditions, and disease stage/previous treatments, each of which should be appropriate to the decision problem. (Caro et al.) |
| 2. | Are competing alternatives clearly described? | The choice of the comparator(s) should always be justified. The comparator should be the most cost-effective alternative intervention currently available. The competing alternatives should be clearly defined in terms of frequency, component services, dose or intensity, duration, and any variations required for target subgroups. |
| 3. | Is a well-defined research question posed in answerable form? | The research question should specify the type of population (participants), type of interventions (and comparisons), the type of outcomes and the type of study that was performed (Higgins et al.) |
| 4. | Is the actual perspective chosen appropriate? | The perspective of the analysis should be stated and defined. Analyses which take a perspective narrower than the societal perspective should report and justify the included and excluded outcomes. (Caro et al.) |
| 5. | Are all important and relevant costs for each alternative identified? | \| The identification of costs should be consistent with the chosen perspective and the assessed area of disease and treatments. For the perspective of the health care payer, at least all direct health care costs associated with or influenced by the competing alternatives must be included. For the societal perspective, also direct and indirect costs outside the health care sector, such as productivity loss, should be included. (adapted from Cleemput et al.) \| \| --- \| |
| 6. | Are all costs measured appropriately in physical units? | Validated sources should be used for the measurement of the resource – and material use, such as observations from clinical trials, prospective observational studies, databases and patient charts, or derived from literature. If derived from literature or studies from other countries, resource use estimates should be validated for the local context. (Cleemput et al.) |
| 7. | Are costs valued appropriately? | All costs should be expressed in values by using prices of a particular year indicated in the study. (adapted from Cleemput et al.) Adjustment for inflation should be based on the Consumer Price Index (CPI) or its health-care component. The method of choice for making adjustments across countries is to use purchasing power parity. However, a simple currency conversion would be appropriate if there is an international market for an input at a fixed price. (Weinstein et al.) |
| 8. | Does the article provide a breakdown of the costs and report costs for each item separately? | Authors should report:  -the list of items that contributed to the total costs  -the costs of each of the items contributing to the total costs |
| 9. | Is an incremental analysis of costs of alternatives performed? | The difference in all relevant costs associated with the alternative treatments should be calculated. Consistency in the perspective and the time horizon should thereby be pursued.  et al.) |
| 10. | Are all future costs discounted appropriately? | The method for discounting costs to present value should be stated and justified. (Weinstein et al.) |
| 11. | Are all important variables, whose values are uncertain, appropriately subjected to sensitivity analysis? | Uncertainty should be analysed using appropriate statistical techniques.  For within-trial economic evaluations and costing studies:  _The sample uncertainty should be presented through deterministic sensitivity analysis methods (point estimate and range).  _Methodological uncertainty coming from the analytical methods chosen such as the discount rate/ missed data imputation etc. should be handled by one-way sensitivity analyses.  The incremental costs and incremental outcomes should be presented with the 95% confidence or credibility interval. (Adapted from Cleemput et al.) |
| 12. | Do the conclusions follow from the data reported? | Do the authors critically discuss the quality of health economic evidence considering the study limitations and uncertainties? |
| 13. | Does the study discuss the generalizability of the results to other settings and patient/client groups? | Generalisability refers to applicability of the results to other populations (e.g. non-trial populations with different baseline risk). Transferability refers to the applicability of the results from other countries. These two aspects should be assessed separately. (Cleemput et al.) |
| 14. | Does the article indicate that there is no potential conflict of interest of study researcher(s) and funder(s)? | No value judgment implied. |
| 15. | Are ethical and distributional issues discussed appropriately? | The morally relevant issues and moral conflicts related to implementing or not implementing the technology have to be synthesized and reported. This includes potential impact on the traditional values such as human equality, autonomy, dignity, the principles of solidarity and justice etc. Summarizing the benefits and harms of introducing/ refraining from the technology for different groups of stakeholders, such as patients, families, care providers, society etc. might be appropriate. The ethical analysis should allow a judgment on their transferability. (adapted from The HTA Core Model) |

# Appendix 7. Expanded CHEC-list: Additional questions on the quality of economic evaluations of physical activity mass media campaigns

| **Item** | **Description** |
| --- | --- |
| 1 | Was the effectiveness measure used appropriate? Did the study where the effectiveness estimate was removed from investigate similar population and mass media campaign with the same characteristics to the one being modelled? |
| 2. | Did the model appropriately consider attenuation of physical activity impact post-campaign? Was any evidence used to support effect of the intervention beyond the trial duration? |
| 3. | Did the study report intermediate measures or use a “stepped approach” to report the results of each step of the model to allow understanding of the impact of each step on the overall results? |

# Appendix 8. GRADE style rating for model-based economic evaluation of physical activity mass media campaigns

Table 7.A. Quality assessment of included economic models

| **Domain** | **Questions and sub-questions** | **Response** | **Notes** |
| --- | --- | --- | --- |
| **A. Quality of model reporting** | Is there a clear and comprehensive description of the model? |  |  |
|  | 1. Is the study population clearly described? | Yes = 1; No = 0 | CHEC Q1 |
|  | 1. Are competing alternatives clearly described? | Yes = 1; No = 0 | CHEC Q2 |
|  | 1. Is a well-defined research question posed in answerable form? | Yes = 1; No = 0 | CHEC Q3 |
|  | 1. Are the structural assumptions and the validation methods of the model properly reported? | Yes = 1; No = 0 | CHEC Q5 |
|  | 1. Does the article indicate that there is no potential conflict of interest of study researcher(s) and funder(s)? | Yes = 1; No = 0 | CHEC Q19 |
|  | **Rating for domain** | POOR/FAIR/ GOOD | 0 to 2 = POOR; 3 or 4 = FAIR; 5 = GOOD |
| **B. Certainty of model inputs** | Has the model used appropriate and reliable inputs? |  |  |
|  | 1. Are all important and relevant outcomes for each alternative identified using systematic methods? | Yes = 1; No = 0 | CHEC Q11 |
|  | 1. Are all health outcomes selected and measured appropriately and have the sources of clinical evidence been critically appraised? | Yes = 1; No = 0 | CHEC Q12, Additional question 1 |
|  | 1. Has the duration of effect been applied appropriately? | Yes = 1; No = 0 | Additional question 2 |
|  | 1. Are outcomes valued appropriately? | Yes = 1; No = 0 | CHEC Q13 |
|  | **Rating for domain** | POOR/FAIR/ GOOD | 0 or 1 = POOR; 2 or 3 = FAIR; 4 = GOOD |
| **C. Credibility of model** | Has an appropriate approach been taken for the modelling and has the validity of this approach been explored? |  |  |
|  | 1. Is the economic study design appropriate to the stated objective? | Yes = 1; No = 0 | CHEC Q4 |
|  | 1. Is the chosen time horizon appropriate in order to include relevant costs and consequences? | Yes = 1; No = 0 | CHEC Q6 |
|  | 1. Is the actual perspective chosen appropriate? | Yes = 1; No = 0 | CHEC Q7 |
|  | 1. Are all important and relevant costs for each alternative identified? | Yes = 1; No = 0 | CHEC Q8 |
|  | 1. Are all costs measured appropriately in physical units? | Yes = 1; No = 0 | CHEC Q9 |
|  | 1. Are costs valued appropriately? | Yes = 1; No = 0 | CHEC Q10 |
|  | 1. Is an incremental analysis of costs and outcomes of alternatives performed? | Yes = 1; No = 0 | CHEC Q14 |
|  | 1. Are all future costs and outcomes discounted appropriately? | Yes = 1; No = 0 | CHEC Q15 |
|  | 1. Are all important variables, whose values are uncertain, appropriately subjected to sensitivity analysis? | Yes = 1; No = 0 | CHEC Q16 |
|  | **Rating for domain** | POOR/FAIR/ GOOD | 0 to 3 = POOR; 4 to 8 = FAIR; 9 = GOOD |
| **D. Certainty of model outputs** | Has the model generated meaningful outputs? |  |  |
|  | 1. Do the authors critically discuss their results including the impact of uncertainty, model design, and limitations of the evidence? | Yes = 1; No = 0 | CHEC Q17 |
|  | 1. Does the study use a “stepped approach” to report the results of each step of the model to allow understanding of the impact of each step on the overall results? | Yes = 1; No = 0 | Additional question 3 |
|  | 1. Does the study discuss the generalizability of the results to other settings and patient/client groups? | Yes = 1; No = 0 | CHEC Q18 |
|  | 1. Are ethical and distributional issues discussed appropriately? | Yes = 1; No = 0 | CHEC Q20 |
|  | **Rating for domain** | POOR/FAIR/ GOOD | 0 or 1 = POOR; 2 or 3 = FAIR; 4 = GOOD |
| **E. Directness of model** | 1. Are the sociodemographic characteristics of the modelled population similar to the population of interest? | Yes = 1; No = 0 | Based on **P**ICOS specific to the question of interest |
|  | 1. Is the modelled intervention similar to the intervention(s) of interest? | Yes = 1; No = 0 | Based on P**I**COS specific to the question of interest |
|  | 1. Does the comparator in the model represent a world without the intervention? | Yes = 1; No = 0 | Based on PI**C**OS specific to the question of interest |
|  | 1. Does the model rely on observed changes in physical activity or antecedents of changes in physical activity? | Yes = 1; No = 0 | Based on PIC**O**S specific to the question of interest |
|  | **Rating for domain** | POOR/FAIR/ GOOD | 0 or 1 = POOR; 2 or 3 = FAIR; 4 = GOOD |

**Table 7.B. Overall judgement of certainty of each economic model for WHO decision-making**

| **Level of certainty** | **Definition** | **How it is derived** |
| --- | --- | --- |
| HIGH | We are confident that the outputs from the model are reliable for decision-making | All domains in Table 1 are rated Good |
| MODERATE | The outputs from the model are likely to be reliable for decision-making, but there is a possibility the outputs are not a reliable prediction of the cost-effectiveness of the intervention | All domains in Table 1 are rated Fair or higher |
| LOW | We have limited confidence that the outputs from the model are reliable for decision-making | One domain in Table 1 is rated Poor but all other domains are rated Fair or Good |
| VERY LOW | We have very little confidence that the outputs from the model are reliable for decision-making | More than one domain in Table 1 is rated Poor |

# Appendix 9. Records excluded at full-text screening and reasons for exclusion

| **Author, Year** | **Reason for exclusion** |
| --- | --- |
| Bemelmans, W., van Baal, P., Wendel-Vos, W., Schuit, J., Feskens, E., Ament, A., & Hoogenveen, R. (2008). The costs, effects and cost-effectiveness of counteracting overweight on a population level. A scientific base for policy targets for the Dutch national plan for action. *Preventive medicine*, *46*(2), 127–132. | Irrelevant outcomes (study targeted obesity prevention and did not report physical activity outcomes separately) |
| Brown, H. S., 3rd, Pérez, A., Li, Y. P., Hoelscher, D. M., Kelder, S. H., & Rivera, R. (2007). The cost-effectiveness of a school-based overweight program. *The international journal of behavioral nutrition and physical activity*, *4*, 47. | Irrelevant intervention (not a PAMMC) |
| Brown, J. D., Wang, C. Y., Groessl, E. J., Pahor, M., & Manini, T. M. (2021). Three-Year, Postintervention, Follow-up Comparison of Health Care Resource Utilization and Costs in the Lifestyle Interventions and Independence for Elders (LIFE) Study. *The journals of gerontology. Series A, Biological sciences and medical sciences*, *76*(2), 272–276. | Irrelevant intervention (not a PAMMC) |
| Cavill, N., Muller, L., Mulhall, C., Rutter, H. (2011). Cycling demonstration towns: A cost-effective investment to promote physical activity. *Obesity Reviews*, 12 (41). | Irrelevant intervention (not a PAMMC) |
| Cecchini, M., Sassi, F., Lauer, J. A., Lee, Y. Y., Guajardo-Barron, V., & Chisholm, D. (2010). Tackling of unhealthy diets, physical inactivity, and obesity: health effects and cost-effectiveness. *Lancet (London, England)*, *376*(9754), 1775–1784. | Irrelevant outcomes (study targeted obesity and did not report PA outcomes separately) |
| Chakravarthy, M. V., & Booth, F. W. (2003). Inactivity and inaction: we can't afford either. *Archives of pediatrics & adolescent medicine*, *157*(8), 731–732. | Irrelevant study design (editorial) |
| Chapman, R., Keall, M., Howden-Chapman, P., Grams, M., Witten, K., Randal, E., & Woodward, A. (2018). A Cost Benefit Analysis of an Active Travel Intervention with Health and Carbon Emission Reduction Benefits. *International journal of environmental research and public health*, *15*(5), 962. | Irrelevant intervention (not a PAMMC but a community active travel intervention supplemented by media publicity) |
| Cocks H. G. (2002). "Sporty" girls and "artistic" boys: friendship, illicit sex, and the British "companionship" advertisement, 1913-1928. *Journal of the history of sexuality*, *11*(3), 457–482. | Irrelevant intervention (not a PAMMC) |
| Coudeyre, E., Tubach, F., Rannou, F., Baron, G., Coriat, F., Brin, S., Revel, M., & Poiraudeau, S. (2007). Effect of a simple information booklet on pain persistence after an acute episode of low back pain: a non-randomized trial in a primary care setting. *PloS one*, *2*(8), e706. | Irrelevant intervention (not a PAMMC) |
| Dalziel, K., & Segal, L. (2007). Time to give nutrition interventions a higher profile: cost-effectiveness of 10 nutrition interventions. *Health promotion international*, *22*(4), 271–283. | Irrelevant intervention (nutritional intervention) |
| De Smedt, D., De Cocker, K., Annemans, L., De Bourdeaudhuij, I., & Cardon, G. (2012). A cost-effectiveness study of the community-based intervention '10 000 Steps Ghent'. *Public health nutrition*, *15*(3), 442–451. | Duplicate (already included in the review) |
| Goodchild R. (1986). The profile of a campaign: "Men Too". *Entre nous (Copenhagen, Denmark)*, (8), 11–13. | Irrelevant intervention (not a PAMMC) |
| Keller, C., Vega-López, S., Ainsworth, B., Nagle-Williams, A., Records, K., Permana, P., & Coonrod, D. (2014). Social marketing: approach to cultural and contextual relevance in a community-based physical activity intervention. *Health promotion international*, *29*(1), 130–140. | Irrelevant intervention (not a PAMMC) |
| Melton, B. F., Bland, H. W., Marshall, E. S., & Bigham, L. E. (2016). The Effectiveness of a Physical Activity Educational Campaign in a Rural Obstetrics and Gynecology Office. *Maternal and child health journal*, *20*(10), 2112–2120. | Irrelevant intervention (not a PAMMC) |
| Meng, L., Xu, H., Liu, A., van Raaij, J., Bemelmans, W., Hu, X., Zhang, Q., Du, S., Fang, H., Ma, J., Xu, G., Li, Y., Guo, H., Du, L., & Ma, G. (2013). The costs and cost-effectiveness of a school-based comprehensive intervention study on childhood obesity in China. *PloS one*, *8*(10), e77971. | Irrelevant intervention (not a PAMMC) |
| Peterson, M., Chandlee, M., & Abraham, A. (2008). Cost-effectiveness analysis of a statewide media campaign to promote adolescent physical activity. *Health promotion practice*, *9*(4), 426–433. | Duplicate (already included in the review) |
| Pringle, A., Cooke, C., Gilson, N., Marsh, K., & McKenna, J. (2010). Cost-effectiveness of interventions to improve moderate physical activity: A study in nine UK sites. *Health Education Journal*, *69*(2), 211–224. | Irrelevant intervention (not a PAMMC) |
| Rootman, I., & Edwards, P. (2004). The best laid schemes of mice and men... ParticipACTION's legacy and the future of physical activity promotion in Canada. *Canadian journal of public health = Revue canadienne de sante publique*, *95 Suppl 2*, S37–S42. | Irrelevant study design (editorial) |
| Sauder R. (1995). Marketing fitness centers. Riverside Health System, Newport News, VA. *Profiles in healthcare marketing*, *11*(3), 1–7. | Irrelevant publication type (Magazine article) |
| Segal, L., Dalton, A.C., Richardson, J. Cost-Effectiveness of the Primary Prevention of Non-Insulin Dependent Diabetes Mellitus, Health Promotion International, Volume 13, Issue 3, 1998, Pages 197–209. | Irrelevant outcomes (study did not report PA outcomes separately) |
| Shilton, T., Rosenberg, M; Maitland, C. (2012). A cost effective way to encourage children to unplug and play. Circulation, Vol. 125, No. 19, Pp E898-E898. | Irrelevant outcome (costs of PAMMC not reported) |
| Van Itallie, A., Corry, K., Vandelanotte, C., Duncan, M. Effectiveness of a social media marketing campaign to increase awareness and membership of a physical activity website. Journal of Science and Medicine in Sport 21S (2018) S77-S98. | Irrelevant outcome (social media clicks/impressions cannot be considered PA antecedents) |
| Wang, L. Y., Yang, Q., Lowry, R., & Wechsler, H. (2003). Economic analysis of a school-based obesity prevention program. *Obesity research*, *11*(11), 1313–1324. | Irrelevant intervention (not a PAMMC) |
| Wright C. C. (1982). Cost containment through health promotion programs. *Journal of occupational medicine: official publication of the Industrial Medical Association*, *24*(12), 965–968. | Irrelevant intervention (not a PAMMC) |
| Zhang, Y. L., Gao, W. G., Pang, Z. C., Sun, J. P., Wang, S. J., Ning, F., Song, X., Kapur, A., & Qiao, Q. (2012). Diabetes self-risk assessment questionnaires coupled with a multimedia health promotion campaign are cheap and effective tools to increase public awareness of diabetes in a large Chinese population. *Diabetic medicine: a journal of the British Diabetic Association*, *29*(11), e425–e429. | Irrelevant intervention (diabetes awareness campaign and the multimedia health promotion campaign did not include PA as a major component) |

PA: Physical activity, PAMMC – Physical Activity Mass Media Campaign

# Appendix 10. Implicit thresholds for willingness to pay for each country provided by WHO team

| **Country** | **Implicit threshold** | **Reference** |
| --- | --- | --- |
| **United States** | High value: ICER < US$50,000/QALY; Intermediate value: ICER US$50,000–150,000/QALY; Low value: ICER >US$150,000/QALY. | Dubois, R.W. (2016). Cost–effectiveness thresholds in the USA: are they coming? Are they already here? *Journal of Comparative Effectiveness Research*. 5: 9–12. |
| **Australia** | High value: ICER < A$45,000/QALY; Intermediate value: ICER A$45,000-A$75,000/QALY; Low value: ICER >A$75,000/QALY. | Mauskopf, J., Chirlia, C., Masaquel, C., Boye, K.S., Bowman, L., Birt, J., Grainger, D. (2013). Relationship between financial impact and coverage of drugs in Australia. *International Journal of Technology Assessment in Health Care.* 29(1): 92–100. |
| **New Zealand** | High value: ICER ≤$38,110/QALY; Low value:  ICER >$38,110/QALY. | Ministry of Health. Appendix: Background Information: New Zealand’s Tobacco Control Programme. Wellington: Ministry of Health; 2016.  <https://www.health.govt.nz/system/files/documents/pages/appendix-8-april-background-info-tobacco-control-programme.pdf>. |
| **Belgium** | High value: ICER ≤€35,000/QALY; Low value:  ICER >35,000/QALY | Pil L, Hoorens I, Vossaert K, Kruse V, Tromme I, Speybroeck N, Annemans L, Brochez L. (2017). Cost-effectiveness and Budget Effect Analysis of a Population-Based Skin Cancer Screening. *JAMA Dermatology.* 153(2):147-153. |
| **Italy** | High value: ICER < €25000/QALY; Intermediate value: ICER €25000-€40000/QALY; Low value: ICER >€40000/QALY. | Associazione Italiana di Economica Sanitaria (AIES): Proposta di linee guida per la valutazione economica degli interventi sanitari. PharmacoEconomics–Italian Res Artic. 2009, 11: 83-93. 10.1007/BF03320660 |

Values and references provided by the WHO team.

ICER: incremental cost-effectiveness ratio, QALY: quality-adjusted life year.

#

# Appendix 11. Application of the GRADE style rating to assess the certainty of each economic model for WHO decision-making

Roux (2008): Quality assessment and level of certainty for WHO decision-making

| **Domain** | **Questions and sub-questions** | **Response** | **Notes** |
| --- | --- | --- | --- |
| **A. Quality of model reporting** | Is there a clear and comprehensive description of the model? |  |  |
|  | 1. Is the study population clearly described? | Yes = 1 | CHEC Q1 |
|  | 1. Are competing alternatives clearly described? | Yes = 1 | CHEC Q2 |
|  | 1. Is a well-defined research question posed in answerable form? | Yes = 1 | CHEC Q3 |
|  | 1. Are the structural assumptions and the validation methods of the model properly reported? | Yes = 1 | CHEC Q5 |
|  | 1. Does the article indicate that there is no potential conflict of interest of study researcher(s) and funder(s)? | Yes = 1 | CHEC Q19 |
|  | **Rating for domain** | **GOOD** | 0 to 2 = POOR; 3 or 4 = FAIR; 5 = GOOD |
| **B. Certainty of model inputs** | Has the model used appropriate and reliable inputs? |  |  |
|  | 1. Are all important and relevant outcomes for each alternative identified using systematic methods? | No = 0 | CHEC Q11 |
|  | 1. Are all health outcomes selected and measured appropriately and have the sources of clinical evidence been critically appraised? | No = 0 | CHEC Q12, Additional question 1 |
|  | 1. Has the duration of effect been applied appropriately? | No = 0 | Additional question 2 |
|  | 1. Are outcomes valued appropriately? | Yes = 1 | CHEC Q13 |
|  | **Rating for domain** | **POOR** | 0 or 1 = POOR; 2 or 3 = FAIR; 4 = GOOD |
| **C. Credibility of model** | Has an appropriate approach been taken for the modelling and has the validity of this approach been explored? |  |  |
|  | 1. Is the economic study design appropriate to the stated objective? | Yes = 1 | CHEC Q4 |
|  | 1. Is the chosen time horizon appropriate in order to include relevant costs and consequences? | Yes = 1 | CHEC Q6 |
|  | 1. Is the actual perspective chosen appropriate? | Yes = 1 | CHEC Q7 |
|  | 1. Are all important and relevant costs for each alternative identified? | Yes = 1 | CHEC Q8 |
|  | 1. Are all costs measured appropriately in physical units? | Yes = 1 | CHEC Q9 |
|  | 1. Are costs valued appropriately? | Yes = 1 | CHEC Q10 |
|  | 1. Is an incremental analysis of costs and outcomes of alternatives performed? | Yes = 1 | CHEC Q14 |
|  | 1. Are all future costs and outcomes discounted appropriately? | Yes = 1 | CHEC Q15 |
|  | 1. Are all important variables, whose values are uncertain, appropriately subjected to sensitivity analysis? | No = 0 | CHEC Q16 |
|  | **Rating for domain** | **FAIR** | 0 to 3 = POOR; 4 to 8 = FAIR; 9 = GOOD |
| **D. Certainty of model outputs** | Has the model generated meaningful outputs? |  |  |
|  | 1. Do the authors critically discuss their results including the impact of uncertainty, model design, and limitations of the evidence? | No = 0 | CHEC Q17 |
|  | 1. Does the study use a “stepped approach” to report the results of each step of the model to allow understanding of the impact of each step on the overall results? | No = 0 | Additional question 3 |
|  | 1. Does the study discuss the generalizability of the results to other settings and patient/client groups? | Yes = 1 | CHEC Q18 |
|  | 1. Are ethical and distributional issues discussed appropriately? | Yes = 1 | CHEC Q20 |
|  | **Rating for domain** | **FAIR** | 0 or 1 = POOR; 2 or 3 = FAIR; 4 = GOOD |
| **E. Directness of model** | 1. Are the sociodemographic characteristics of the modelled population similar to the population of interest? | Yes = 1 | Based on **P**ICO specific to the question of interest |
|  | 1. Is the modelled intervention similar to the intervention(s) of interest? | No = 0 | Based on P**I**CO specific to the question of interest |
|  | 1. Does the comparator in the model represent a world without the intervention? | Yes = 1 | Based on PI**C**O specific to the question of interest |
|  | 1. Does the model rely on observed changes in physical activity or antecedents of changes in physical activity? | Yes = 1 | Based on PIC**O** specific to the question of interest |
|  | **Rating for domain** | **FAIR** | 0 to 1 = POOR; 2 or 3 = FAIR; 4 = GOOD |

**Level of certainty:** LOW. We have limited confidence that the outputs from the model are reliable for decision-making **Cobiac (2009):** Quality assessment and level of certainty for WHO decision-making

| **Domain** | **Questions and sub-questions** | **Response** | **Notes** |
| --- | --- | --- | --- |
| **A. Quality of model reporting** | Is there a clear and comprehensive description of the model? |  |  |
|  | 1. Is the study population clearly described? | Yes = 1 | CHEC Q1 |
|  | 1. Are competing alternatives clearly described? | Yes = 1 | CHEC Q2 |
|  | 1. Is a well-defined research question posed in answerable form? | Yes = 1 | CHEC Q3 |
|  | 1. Are the structural assumptions and the validation methods of the model properly reported? | No = 0 | CHEC Q5 |
|  | 1. Does the article indicate that there is no potential conflict of interest of study researcher(s) and funder(s)? | Yes = 1 | CHEC Q19 |
|  | **Rating for domain** | **FAIR** | 0 to 2 = POOR; 3 or 4 = FAIR; 5 = GOOD |
| **B. Certainty of model inputs** | Has the model used appropriate and reliable inputs? |  |  |
|  | 1. Are all important and relevant outcomes for each alternative identified using systematic methods? | Yes = 1 | CHEC Q11 |
|  | 1. Are all health outcomes selected and measured appropriately and have the sources of clinical evidence been critically appraised? | Yes = 1 | CHEC Q12, Additional question 1 |
|  | 1. Has the duration of effect been applied appropriately? | No = 0 | Additional question 2 |
|  | 1. Are outcomes valued appropriately? | Yes = 1 | CHEC Q13 |
|  | **Rating for domain** | **FAIR** | 0 or 1 = POOR; 2 or 3 = FAIR; 4 = GOOD |
| **C. Credibility of model** | Has an appropriate approach been taken for the modelling and has the validity of this approach been explored? |  |  |
|  | 1. Is the economic study design appropriate to the stated objective? | Yes = 1 | CHEC Q4 |
|  | 1. Is the chosen time horizon appropriate in order to include relevant costs and consequences? | Yes = 1 | CHEC Q6 |
|  | 1. Is the actual perspective chosen appropriate? | No = 0 | CHEC Q7 |
|  | 1. Are all important and relevant costs for each alternative identified? | Yes = 1 | CHEC Q8 |
|  | 1. Are all costs measured appropriately in physical units? | Yes = 1 | CHEC Q9 |
|  | 1. Are costs valued appropriately? | Yes = 1 | CHEC Q10 |
|  | 1. Is an incremental analysis of costs and outcomes of alternatives performed? | Yes = 1 | CHEC Q14 |
|  | 1. Are all future costs and outcomes discounted appropriately? | Yes = 1 | CHEC Q15 |
|  | 1. Are all important variables, whose values are uncertain, appropriately subjected to sensitivity analysis? | No = 0 | CHEC Q16 |
|  | **Rating for domain** | **FAIR** | 0 to 3 = POOR; 4 to 8 = FAIR; 9 = GOOD |
| **D. Certainty of model outputs** | Has the model generated meaningful outputs? |  |  |
|  | 1. Do the authors critically discuss their results including the impact of uncertainty, model design, and limitations of the evidence? | No = 0 | CHEC Q17 |
|  | 1. Does the study use a “stepped approach” to report the results of each step of the model to allow understanding of the impact of each step on the overall results? | No = 0 | Additional question 3 |
|  | 1. Does the study discuss the generalizability of the results to other settings and patient/client groups? | Yes = 1 | CHEC Q18 |
|  | 1. Are ethical and distributional issues discussed appropriately? | No = 0 | CHEC Q20 |
|  | **Rating for domain** | **POOR** | 0 or 1 = POOR; 2 or 3 = FAIR; 4 = GOOD |
| **E. Directness of model** | 1. Are the sociodemographic characteristics of the modelled population similar to the population of interest? | Yes = 1 | Based on **P**ICO specific to the question of interest |
|  | 1. Is the modelled intervention similar to the intervention(s) of interest? | Yes = 1 | Based on P**I**CO specific to the question of interest |
|  | 1. Does the comparator in the model represent a world without the intervention? | Yes = 1 | Based on PI**C**O specific to the question of interest |
|  | 1. Does the model rely on observed changes in physical activity or antecedents of changes in physical activity? | Yes = 1 | Based on PIC**O** specific to the question of interest |
|  | **Rating for domain** | **GOOD** | 0 or 1 = POOR; 2 or 3 = FAIR; 4 = GOOD |

**Level of certainty:** LOW. We have limited confidence that the outputs from the model are reliable for decision-making

**De Smedt (2011):** Quality assessment and level of certainty for WHO decision-making

| **Domain** | **Questions and sub-questions** | **Response** | **Notes** |
| --- | --- | --- | --- |
| **A. Quality of model reporting** | Is there a clear and comprehensive description of the model? |  |  |
|  | 1. Is the study population clearly described? | Yes = 1 | CHEC Q1 |
|  | 1. Are competing alternatives clearly described? | Yes = 1 | CHEC Q2 |
|  | 1. Is a well-defined research question posed in answerable form? | Yes = 1 | CHEC Q3 |
|  | 1. Are the structural assumptions and the validation methods of the model properly reported? | No = 0 | CHEC Q5 |
|  | 1. Does the article indicate that there is no potential conflict of interest of study researcher(s) and funder(s)? | Yes = 1 | CHEC Q19 |
|  | **Rating for domain** | **FAIR** | 0 to 2 = POOR; 3 or 4 = FAIR; 5 = GOOD |
| **B. Certainty of model inputs** | Has the model used appropriate and reliable inputs? |  |  |
|  | 1. Are all important and relevant outcomes for each alternative identified using systematic methods? | No = 0 | CHEC Q11 |
|  | 1. Are all health outcomes selected and measured appropriately and have the sources of clinical evidence been critically appraised? | No = 0 | CHEC Q12, Additional question 1 |
|  | 1. Has the duration of effect been applied appropriately? | Yes = 1 | Additional question 2 |
|  | 1. Are outcomes valued appropriately? | Yes = 1 | CHEC Q13 |
|  | **Rating for domain** | **FAIR** | 0 or 1 = POOR; 2 or 3 = FAIR; 4 = GOOD |
| **C. Credibility of model** | Has an appropriate approach been taken for the modelling and has the validity of this approach been explored? |  |  |
|  | 1. Is the economic study design appropriate to the stated objective? | Yes = 1 | CHEC Q4 |
|  | 1. Is the chosen time horizon appropriate in order to include relevant costs and consequences? | Yes = 1 | CHEC Q6 |
|  | 1. Is the actual perspective chosen appropriate? | No = 0 | CHEC Q7 |
|  | 1. Are all important and relevant costs for each alternative identified? | Yes = 1 | CHEC Q8 |
|  | 1. Are all costs measured appropriately in physical units? | Yes = 1 | CHEC Q9 |
|  | 1. Are costs valued appropriately? | Yes = 1 | CHEC Q10 |
|  | 1. Is an incremental analysis of costs and outcomes of alternatives performed? | Yes = 1 | CHEC Q14 |
|  | 1. Are all future costs and outcomes discounted appropriately? | Yes = 1 | CHEC Q15 |
|  | 1. Are all important variables, whose values are uncertain, appropriately subjected to sensitivity analysis? | Yes = 1 | CHEC Q16 |
|  | **Rating for domain** | **FAIR** | 0 to 3 = POOR; 4 to 8 = FAIR; 9 = GOOD |
| **D. Certainty of model outputs** | Has the model generated meaningful outputs? |  |  |
|  | 1. Do the authors critically discuss their results including the impact of uncertainty, model design, and limitations of the evidence? | Yes = 1 | CHEC Q17 |
|  | 1. Does the study use a “stepped approach” to report the results of each step of the model to allow understanding of the impact of each step on the overall results? | No = 0 | Additional question 3 |
|  | 1. Does the study discuss the generalizability of the results to other settings and patient/client groups? | No = 0 | CHEC Q18 |
|  | 1. Are ethical and distributional issues discussed appropriately? | No = 0 | CHEC Q20 |
|  | **Rating for domain** | **POOR** | 0 or 1 = POOR; 2 or 3 = FAIR; 4 = GOOD |
| **E. Directness of model** | 1. Are the sociodemographic characteristics of the modelled population similar to the population of interest? | Yes = 1 | Based on **P**ICO specific to the question of interest |
|  | 1. Is the modelled intervention similar to the intervention(s) of interest? | No = 0 | Based on P**I**CO specific to the question of interest |
|  | 1. Does the comparator in the model represent a world without the intervention? | Yes = 1 | Based on PI**C**O specific to the question of interest |
|  | 1. Does the model rely on observed changes in physical activity or antecedents of changes in physical activity? | Yes = 1 | Based on PIC**O** specific to the question of interest |
|  | **Rating for domain** | **FAIR** | 0 or 1 = POOR; 2 or 3 = FAIR; 4 = GOOD |

**Level of certainty:** LOW. We have limited confidence that the outputs from the model are reliable for decision-making

**Goryakin (2019):** Quality assessment and level of certainty for WHO decision-making

| **Domain** | **Questions and sub-questions** | **Response** | **Notes** |
| --- | --- | --- | --- |
| **A. Quality of model reporting** | Is there a clear and comprehensive description of the model? |  |  |
|  | 1. Is the study population clearly described? | Yes = 1 | CHEC Q1 |
|  | 1. Are competing alternatives clearly described? | Yes = 1 | CHEC Q2 |
|  | 1. Is a well-defined research question posed in answerable form? | Yes = 1 | CHEC Q3 |
|  | 1. Are the structural assumptions and the validation methods of the model properly reported? | Yes = 1 | CHEC Q5 |
|  | 1. Does the article indicate that there is no potential conflict of interest of study researcher(s) and funder(s)? | No = 0 | CHEC Q19 |
|  | **Rating for domain** | **FAIR** | 0 to 2 = POOR; 3 or 4 = FAIR; 5 = GOOD |
| **B. Certainty of model inputs** | Has the model used appropriate and reliable inputs? |  |  |
|  | 1. Are all important and relevant outcomes for each alternative identified using systematic methods? | Yes = 1 | CHEC Q11 |
|  | 1. Are all health outcomes selected and measured appropriately and have the sources of clinical evidence been critically appraised? | No = 0 | CHEC Q12, Additional question 1 |
|  | 1. Has the duration of effect been applied appropriately? | No = 0 | Additional question 2 |
|  | 1. Are outcomes valued appropriately? | Yes = 1 | CHEC Q13 |
|  | **Rating for domain** | **FAIR** | 0 or 1 = POOR; 2 or 3 = FAIR; 4 = GOOD |
| **C. Credibility of model** | Has an appropriate approach been taken for the modelling and has the validity of this approach been explored? |  |  |
|  | 1. Is the economic study design appropriate to the stated objective? | Yes = 1 | CHEC Q4 |
|  | 1. Is the chosen time horizon appropriate in order to include relevant costs and consequences? | Yes = 1 | CHEC Q6 |
|  | 1. Is the actual perspective chosen appropriate? | No = 0 | CHEC Q7 |
|  | 1. Are all important and relevant costs for each alternative identified? | Yes = 1 | CHEC Q8 |
|  | 1. Are all costs measured appropriately in physical units? | Yes = 1 | CHEC Q9 |
|  | 1. Are costs valued appropriately? | No = 0 | CHEC Q10 |
|  | 1. Is an incremental analysis of costs and outcomes of alternatives performed? | Yes = 1 | CHEC Q14 |
|  | 1. Are all future costs and outcomes discounted appropriately? | Yes = 1 | CHEC Q15 |
|  | 1. Are all important variables, whose values are uncertain, appropriately subjected to sensitivity analysis? | No = 0 | CHEC Q16 |
|  | **Rating for domain** | **FAIR** | 0 to 3 = POOR; 4 to 8 = FAIR; 9 = GOOD |
| **D. Certainty of model outputs** | Has the model generated meaningful outputs? |  |  |
|  | 1. Do the authors critically discuss their results including the impact of uncertainty, model design, and limitations of the evidence? | No = 0 | CHEC Q17 |
|  | 1. Does the study use a “stepped approach” to report the results of each step of the model to allow understanding of the impact of each step on the overall results? | No = 0 | Additional question 3 |
|  | 1. Does the study discuss the generalizability of the results to other settings and patient/client groups? | No = 0 | CHEC Q18 |
|  | 1. Are ethical and distributional issues discussed appropriately? | No = 0 | CHEC Q20 |
|  | **Rating for domain** | **POOR** | 0 or 1 = POOR; 2 or 3 = FAIR; 4 = GOOD |
| **E. Directness of model** | 1. Are the sociodemographic characteristics of the modelled population similar to the population of interest? | No = 1 | Based on **P**ICO specific to the question of interest |
|  | 1. Is the modelled intervention similar to the intervention(s) of interest? | No = 0 | Based on P**I**CO specific to the question of interest |
|  | 1. Does the comparator in the model represent a world without the intervention? | Yes = 1 | Based on PI**C**O specific to the question of interest |
|  | 1. Does the model rely on observed changes in physical activity or antecedents of changes in physical activity? | No = 1 | Based on PIC**O** specific to the question of interest |
|  | **Rating for domain** | **POOR** | 0 to 1 = POOR; 2 or 3 = FAIR; 4 = GOOD |

**Level of certainty:** VERY LOW. We have very little confidence that the outputs from the model are reliable for decision-making

**Mizdrak (2020):** Quality assessment and level of certainty for WHO decision-making

| **Domain** | **Questions and sub-questions** | **Response** | **Notes** |
| --- | --- | --- | --- |
| **A. Quality of model reporting** | Is there a clear and comprehensive description of the model? |  |  |
|  | 1. Is the study population clearly described? | Yes = 1 | CHEC Q1 |
|  | 1. Are competing alternatives clearly described? | Yes = 1 | CHEC Q2 |
|  | 1. Is a well-defined research question posed in answerable form? | Yes = 1 | CHEC Q3 |
|  | 1. Are the structural assumptions and the validation methods of the model properly reported? | No = 0 | CHEC Q5 |
|  | 1. Does the article indicate that there is no potential conflict of interest of study researcher(s) and funder(s)? | Yes = 1 | CHEC Q19 |
|  | **Rating for domain** | **FAIR** | 0 to 2 = POOR; 3 or 4 = FAIR; 5 = GOOD |
| **B. Certainty of model inputs** | Has the model used appropriate and reliable inputs? |  |  |
|  | 1. Are all important and relevant outcomes for each alternative identified using systematic methods? | No = 0 | CHEC Q11 |
|  | 1. Are all health outcomes selected and measured appropriately and have the sources of clinical evidence been critically appraised? | No = 0 | CHEC Q12, Additional question 1 |
|  | 1. Has the duration of effect been applied appropriately? | No = 0 | Additional question 2 |
|  | 1. Are outcomes valued appropriately? | No = 0 | CHEC Q13 |
|  | **Rating for domain** | **POOR** | 0 to 1 = POOR; 2 or 3 = FAIR; 4 = GOOD |
| **C. Credibility of model** | Has an appropriate approach been taken for the modelling and has the validity of this approach been explored? |  |  |
|  | 1. Is the economic study design appropriate to the stated objective? | Yes = 1 | CHEC Q4 |
|  | 1. Is the chosen time horizon appropriate in order to include relevant costs and consequences? | Yes = 1 | CHEC Q6 |
|  | 1. Is the actual perspective chosen appropriate? | No = 0 | CHEC Q7 |
|  | 1. Are all important and relevant costs for each alternative identified? | Yes = 1 | CHEC Q8 |
|  | 1. Are all costs measured appropriately in physical units? | Yes = 1 | CHEC Q9 |
|  | 1. Are costs valued appropriately? | No = 0 | CHEC Q10 |
|  | 1. Is an incremental analysis of costs and outcomes of alternatives performed? | Yes = 1 | CHEC Q14 |
|  | 1. Are all future costs and outcomes discounted appropriately? | Yes = 1 | CHEC Q15 |
|  | 1. Are all important variables, whose values are uncertain, appropriately subjected to sensitivity analysis? | No = 0 | CHEC Q16 |
|  | **Rating for domain** | **FAIR** | 0 to 3 = POOR; 4 to 8 = FAIR; 9 = GOOD |
| **D. Certainty of model outputs** | Has the model generated meaningful outputs? |  |  |
|  | 1. Do the authors critically discuss their results including the impact of uncertainty, model design, and limitations of the evidence? | No = 0 | CHEC Q17 |
|  | 1. Does the study use a “stepped approach” to report the results of each step of the model to allow understanding of the impact of each step on the overall results? | No = 0 | Additional question 3 |
|  | 1. Does the study discuss the generalizability of the results to other settings and patient/client groups? | No = 0 | CHEC Q18 |
|  | 1. Are ethical and distributional issues discussed appropriately? | Yes = 1 | CHEC Q20 |
|  | **Rating for domain** | **POOR** | 0 or 1 = POOR; 2 or 3 = FAIR; 4 = GOOD |
| **E. Directness of model** | 1. Are the sociodemographic characteristics of the modelled population similar to the population of interest? | Yes = 1 | Based on **P**ICO specific to the question of interest |
|  | 1. Is the modelled intervention similar to the intervention(s) of interest? | No = 0 | Based on P**I**CO specific to the question of interest |
|  | 1. Does the comparator in the model represent a world without the intervention? | Yes = 1 | Based on PI**C**O specific to the question of interest |
|  | 1. Does the model rely on observed changes in physical activity or antecedents of changes in physical activity? | No = 0 | Based on PIC**O** specific to the question of interest |
|  | **Rating for domain** | **FAIR** | 0 to 1 = POOR; 2 or 3 = FAIR; 4 = GOOD |

**Level of certainty:** VERY LOW. We have very little confidence that the outputs from the model are reliable for decision-making

# Appendix 12. Additional resources on Agita São Paulo campaign

**SOURCE 1: PROGRAM WEBSITE**

**Intervention costs**

Budget**:** US$150,000 to US$400,000 per year. This is an estimated budget by the project team in the initial years of the Agita program, when it was maximally using comprehensive strategies including mass media. It represents an investment of approximately less than US$0.01 per state inhabitant per year. In contrast, the estimated costs of illness related to a sedentary lifestyle in the state are about US$1.00 per person per year.

Costs breakdown**:**

- Education and marketing material: 40%
- Human resources: 35%
- Research: 14%
- Logistics materials and services (eg. postage, photocopying and printing): 11%
- Volunteering: There is also a large volunteering component, with academics and professionals donating their time to the research and organisation and management of media and community programmatic activities.
- Partner institutions: the program has acquired more than 300 partner institutions. The partners can carry out activities that require direct financial investment as well as ones that do not require investment. Partners can pay to produce special educational materials such as flyers, posters, videos and T-shirts that contain ‘Agita’ and the institution’s insignia, to use with their own employees or for events that they organise in the community. Other partner activities carry no additional cost, such as printing the message of ‘accumulate 30 minutes of physical activity every day’ on the institution’s letterhead, employee pay stubs, or magazine or newsletter. Partners can also put up posters or distribute flyers among their employees and organise lectures or workshops about physical activity and health.
- Non-paid media and other communication channels (e.g. putting the Agita São Paulo message on electricity company bills, in the football stadium and at metro stations): no costs available for these and these are achieved via collaboration with partner organisation.
- Mega events: costs not available for these. These, especially the annual mass walking events in Sao Paulo, were an important component of the communications of the message, and they also attracted substantial earned media, but the potential cost of these mega events was not costed. Unsure if they are included in the annual budget and how much the partners contribute.

**Model developed to assess the cost-effectiveness (supported by World Bank, CDC and CELAFISCS)**

**Model inputs:**

- Programme costs;
- State-wide physical activity surveys carried out from 1999 to 2003;
- Medical costs from São Paulo and Brazilian national data sets;
- Relative risks and incidence of disease from the international scientific literature.

**Results**

Intervention costs (cost/person/year)**:** R$640 (currency year 2013).

Impact on physical activity: 132 metabolic equivalent of task (MET) minutes per week, per person, for each year of the programme’s delivery.

Cost-effectiveness: incremental cost-effectiveness ration (ICER) < R$50,000/QALY (Quality Adjusted Life Year), which is a rate many accept as demonstrating a cost-effective public health intervention.

**Review authors comments**: we were unable to find peer-reviewed publications with additional information on intervention costs and cost-effectiveness.

**Reference:** <https://thensmc.com/resources/showcase/agita-s%C3%A3o-paulo>, Access date: 11 October 2021.

**SOURCE 2: WORLD BANK REPORT 2005 - MODEL-BASED ANALYSIS OF AGITA SÃO PAULO**

Study type: unclear. Cost-consequence analysis and cost-effectiveness analysis (classified by authors)

**Model parameters**

Time horizon: 5 years (2005 to 2009) disease incidence. Long-term impact in DALYs

Perspective: societal (assumed).

Physical activity impact estimate source and assumptions: review conducted for the report (details not available and not published). Agita Sao Paulo effectiveness estimate retrieved from a CDC report (ref 46): 3.2% reduction in reducing physical inactivity.

Future health benefits of physical activity: Diabetes Mellitus, ischaemic stroke, ischaemic heart disease.

Costs considered:

-Intervention costs

-Cost of treating patients with non-communicable diseases.

-Productivity losses due to non-communicable diseases.

Parameters:

- Future burden of disease: based on the country-specific findings of Global Burden of Disease Study (GBDS) and adjusted according to estimates used on the “Projeto de Carga de Doença do Brasil” (PCDB).
- Productivity loss: GBDS and human capital approach assuming productivity losses per year of life lost and per year of inability to work as per capita GDP.
- Financial costs were based on information about the average length of life with a certain disease retrieved from the GBDS and PCDB and average annual treatment costs for individual diseases.
- Currency (year): US dollar (2000)
- Discount: 3%
- Target population: unclear (25% of the whole Brazilian population assumed).

**Intervention costs**

- Cost/person: US$0.004 per person; US$0.06 per person including voluntary contributions of organizations and individuals (source: report prepared for the World Bank, not published).
- Total cost for scaling up the intervention: $33,485,000 ($4,186,000 to $62,784,000).

**Net cost-savings**: $314,340,000 (241,560,000 to 387,120,000)

**Key findings**: “Scaling up comprehensive community campaigns to promote physical activity

would generate the second largest impact on both disease burden and costs. It would

result in a reduction of the non-communicable disease burden by approximately 135,000

DALYs. I t would reduce treatment costs for non-communicable diseases by US$ 348

million and avoid financial and economic losses of US$622 million.”

**Benefit cost-ratio** (savings of economic costs over the costs of scaling up the intervention): 18.6 (range 8.7 to >100)

**Cost-effectiveness ratio** (total intervention costs / DALY averted): $247 (range $27 to $528)

**Review authors comments:** we were unable to find peer-reviewed publication reporting the data provided. The overall approach to the model is described in the main report and in Annex 2, however no details are provided on assumptions and sources of parameters. The lack of details on the model makes it hard to interpret the results, assess the quality of the model and the certainty of the evidence.

**Reference***:* “World Bank. 2005. Brazil: Addressing the Challenge of Non-Communicable Diseases in Brazil. Washington, DC. © World Bank. https://openknowledge.worldbank.org/handle/10986/8334 License: CC BY 3.0 IGO.”

#

# Appendix table 1. Characteristics of studies included in this review according to study type: model-based analyses and costing studies

| **Author (Year), Income / Type of study** | **Campaign name / Location / Classification** | **Media channels / Messages / Duration** | **Study sample / Target population** | **Comparator** | **Impact on physical activity / Economic analysis outcome^a^** |
| --- | --- | --- | --- | --- | --- |
| **Model-based** |  |  |  |  |  |
| Roux (2008) ^b^,  “High income”  Effectiveness study: Reger (2002)^[[1]](#footnote-2)^  Quasi-experimental  Model name: CDC MOVE PA policy model (Markov model 40 years) | **Name:** Wheeling Walks  **Location:** US  **Effectiveness study:** Single community in West Virginia, US  **Classification:** CWI with MMC | **Channels:** TV ads (n=2, aired 683 times, 5104 GRPs), radio ads (aired 1,988 times on 12 stations, 3461 GRPs), newspaper ads (n=14), public relations  **Messages: M**essages were designed to produce perceived control belief change regarding walking for exercise  **Additional components**: Primary care initiatives; community programs; cross sectoral initiatives; website resources; worksites.  **Duration:** 8 wks, one off (same as effectiveness study: April 2001 to June 2001)  **Horizon:** 40 years | **n:** US population  **Age:** Adults aged 25-64 years  **% female:** NR  **Target:** Adults (25-64 years)  **Baseline PA level:** PA levels in the US population (MET-min/wk range): 20% inactive (0-150), Irregularly active 33.6% (151-855), 26.5% Meets guideline (856-2280) and 19.9% highly active (>2280). The intervention-specific METminutes was added to current levels, and the % of the cohort that moved to another PA level as a result of the intervention was noted. This percentage was then used as a transition probability | No intervention  Effectiveness study: control city (Parkersburg) | **Impact on PA:** PA behaviour - 316 MET-min/wk at 1 year (estimated by authors based on effectiveness study)  **Economic analysis outcome:** Average 0.031 LY gained and 0.049 QALY gained at 40 years |
| Cobiac (2009) ^b^,  **“**High income”  Effectiveness study: Bauman (2001)^[[2]](#footnote-3)^  Quasi-experimental  Model details not provided | **Name:** Exercise, you only have to take it regularly not seriously  **Location:** Australia  **Classification:** mostly MM | **Channels:** Television, radio, newspaper, magazines  **Messages:** "Moderate 30 min/day", "3 times 20 minutes each of vigorous activity daily necessary for health", "exercise, you only have to take it regularly, not seriously"  **Additional components:** Targeted material to primary care doctors; sport helpline; community programs and merchandise  **Duration:** 6 wks, one-off (same as effectiveness study February to March 1998)  **Horizon**: Lifetime of the Australian population in a baseline year of 2003 | **n:** 9.74 mi (Australian population aged 25-60 years)  **Age:** 25 to 60 years old  **% female:** NR  **Target:** Adults (age not specified)  **Baseline PA level**: National PA level data (collected via surveys) according to age and 15 age groups was used. | No intervention  Effectiveness study: control cities | **Impact on PA:** PA behaviour - 148 MET-min/wk at 1 year (estimated by authors based on effectiveness study)  **Economic analysis outcome:** Total 23,000 (95% UI: 7,600 to 40,000) DALYs averted over the lifetime of the population |
| De Smedt (2011),  “High income”  Effectiveness study: De Cocker (2007)^[[3]](#footnote-4)^  Pre-post design  Markov model (20 years) | **Name:** 10,000 Steps Ghent  **Location:** Ghent, Belgium  **Classification:** CWI with MMC | **Channels**: Press conferences (n=7), newspapers, local magazine (n=1), periodical delivered to every household (n=1), local newscasts (n=6), billboards (n=20)  **Messages:** 10,000 steps/day, at least 30min of moderate-intensity PA most days of the wk; more-vigorous activity or sport for a minimum of 20 min 3 days/wk  **Additional components**: Website; signage, sale and loan of pedometers; workplace projects; promotion materials sent to older people, schools, GPs, dieticians and physical therapists.  **Duration:** 20 years - Intervention implemented every year and pedometer every 5 years for 1 year (effectiveness study March 2005 to March 2006)  **Horizon:** 20 years | **n:** 245,000 (Ghent adult population)  **Age:** 25-75 (mean 48, SD 11) years old  **% female:** 54  **Target:** Adults (25-75 years)  **Baseline PA level:** Unclear | No intervention  Effectiveness study: control city (population of 77,000) | **Impact on PA:** PA behaviour - Walking min/wk mean difference: 66 (95% CI: 28 to 104) (effectiveness study)  **Economic analysis outcome:** Average QALY gained: 0.16 (men) and 0.11 (women) at 20 years |
| Goryakin (2019) ^b^,  “High income”  Effectiveness estimate source: Goryakin (2017)^[[4]](#footnote-5)^  Report  Model name: OECD SHPeP-NCD^[[5]](#footnote-6),^^[[6]](#footnote-7)^ (microsimulation 31 years) | **Name:** NR Hypothetical campaign (campaign components and effectiveness extracted from previous campaigns reported on 12 papers)  **Location:** Italy  **Classification:** mostly MM | **Channels:** Radio, television and newspapers/magazines. Two 15-second television paid commercials (Bauman et al. 2001)^[[7]](#footnote-8)^ combined with some other resources, such as advertisements in printed media, posters, leaflets, postcards, web sites and public relations events^1,^ ^[[8]](#footnote-9)^, as in the Active For Life campaign in Hillsdon et al., 2001^[[9]](#footnote-10)^  **Messages:** Promotion of active lifestyle (details NR)  **Additional components**: Unclear  **Duration:** 6 segments of three years each in 31 years (2019-2050)^2^ The intervention was made available at the beginning of the 3-year period (assumed by review authors)  **Median duration** of the interventions used to create hypothetical intervention: 18 wks  **Horizon:** 31 years | **n:** Italy adult population (details NR)  **Age:** NR  **% female:** NR  **Target:** adults (> 18 years)  **Baseline PA level:** Exact proportion of people who were considered “active” and the definition of active are unclear | No intervention | **Impact on PA:** PA behaviour - 35.55% increase in people who are considered at least moderately active in relation to the proportion of people who were at least moderately active at the beginning of the intervention (based on literature review)^1^  **Economic analysis outcome:** Cumulative DALYs gained 86,000 at 31 years |
| Mizdrak (2020),  “High income”  Effectiveness estimate source: Gal (2018)^[[10]](#footnote-11)^  Multistate lifestate model | **Name:** Hypothetical one-off national mass media campaign to promote the use of PA apps (campaign features created by authors)  **Location:** New Zealand  **Classification**: Mass media to support the use of PA apps, Primary PA | **Channels:** Not reported  **Messages:** Promotion of PA apps  **Additional components**: Unclear  **Duration:** 1 year, one-off (assumed by reviewers)  **Horizon:** Lifetime of the New Zealand population in a baseline year of 2011 | **n:** NZ population (15-79 years)  **Age:** 15-79 years  **% female:** NR  **Target:** Adults  **Baseline PA level:** The PA distribution of the New Zealand (NZ) adult population was estimated by converting responses to the NZ PA Questionnaire Short Form in the NZ Health Survey to MET minutes per week of moderate and vigorous physical activity | No intervention | **Impact on PA:** PA behaviour - 285 (SD 43) MVPA MET-min/wk for those who adhered to the app (estimated by authors based on a previous review)  **Economic analysis outcome:** Total QALY gained 28 (95% UI 8-72) over the lifetime of the 2011 population, or 0.008 QALYs gained per 1000 people |
| **Costing studies** |  |  |  |  |  |
| Hillsdon (2001)  “High income”  Before and after study (3 cross-sectional annual surveys) | **Name:** ACTIVE for LIFE  **Location:** UK  **Classification:** mostly MM | **Channels:** Television, magazines  **Messages:** Adults should aim to take part in at least 5 sessions of 30 minutes of moderate intensity PA per week.  **Additional components:** A network to encourage 8500 professionals to promote PA, which provided support with information, fact sheets, newsletters, research results and seminars; Public relations and promotions including media advocacy, national press launches, national roadshows, competitions, workplace promotions and co-promotions with major retailers and manufacturers.  **Duration:** 1996-1998 (one-off). Advertisement was aired over a six-week period in 1996. The duration of advertisements between 1997 and 1998 and the duration of the additional components are unclear. | **n:** 3,189  **Age:** 16-24 – 7.5%, 25-34 – 17.8%, 35-44 – 19.6%, 45-54 – 19.4%, 55-64 – 17.1% and ≥65 – 18.6%.  **% female:** 57.5%  **Target:** Adults aged 16-74 years, with a focus on women aged 16-24, men aged 45-55 and men and women aged >50  **Baseline PA level:** 31.4% met guidelines for moderate (moderate intensity PA for at least 30 min, ≥5 days/wk) and/or vigorous PA (vigorous intensity PA for at least 20 min, ≥3 days/wk) | Annual cross-sectional data from population surveys between 1995 and 1997 | **Impact on PA** ^c^**:** PA behaviour – The change in proportion of people who met guideline recommendations for moderate or vigorous PA between waves 1 and 3 was -9.8% (95% CI: -7.9% to -11.7%). There were significant differences in overall PA levels at waves 1 and 3 (*p*=0.0009), with 8.8% fewer people active at a vigorous level at wave 3 and 6.8% more people being classified as sedentary. |
| Bauman (2003),  “High income”  Before and after study (4 cross-sectional annual surveys) | **Name:** Push Play  **Location:** New Zealand  **Classification:** mostly MM | **Channels:** Television, radio, magazine, billboards, a national Push Play Day  **Messages:** Promotion of PA as part of daily life. The campaign targeted all adults, particularly the middle aged, and males (30–54 years)  **Additional components**: Mail-out to inform primary care physicians about the new moderate-intensity PA message; PA counselling kits to family physicians; community-based walking and PA events  **Duration:** 1999-2002 (one-off). This paper reports cross-sectional population data regarding the public awareness of the campaign from 1999-2002) | **n:** Range of participants in the 4 annual surveys: 504 to 665  **Age:** % Aged < 34 years: 31%, 33%, 34% and 34% in 1999, 2000, 2001 and 2002, respectively.  **% female:** 54.1  **Target**: Adults, but particularly the middle aged and males 30-54 years  **Baseline PA level:** 38.6% reported being active for at least 30 min for ≥5 days/wk | Annual cross-sectional data from population surveys between 1999-2002 | **Impact on PA** ^c^**:** PA behaviour - % of people who reported being active for at least 30 min for ≥5 days/wk (%, adjusted OR (95% CI): 44.5% 1.32 (1.05-1.68) from 1999 to 2000, 40.8% 1.10 (0.87-1.40) from 2000 to 2001, 8% 1.03 (0.81-1.31) from 2001 to 2002. |
| MahechaMatsudo (2003),  “Upper middle income country”  Cross-sectional survey^1^ | **Name:** Agita São Paulo  **Location:** São Paulo, Brazil  **Classification:** CWI with MMC | **Channels:** Mega-events, which attracted incidental media coverage (television, radio, magazines, newspapers and internet)  **Messages:** Promotion of an active lifestyle by accumulating at least 30 minutes of moderate PA per day, on most days of the week  **Additional components**: Community programs (e.g. lectures or workshops about PA and health, school programs, workplace PA programs)  **Duration:** Multi-year campaign (5^th^ December in 1996 – ongoing) | **n:** 37 million people (State of São Paulo’s population)  **Age:** NR  **% female:** NR  **Target:** Students, workers and elderly  **Baseline PA level:** NR | Unexposed respondents vs exposed respondents | **Impact on PA** ^c^**:** PA behaviour – 54.2% of people who were familiar with the Program reported to be very active (≥5 days/wk and ≥30 min/session) or active (≥3 days/wk and ≥20min/session), versus 31.9% among those not familiar with the Program^[[11]](#footnote-12)^  The impact of the program when delivered to specific and smaller groups were reported, but it is unclear if budget reported also cover the costs with these programs. |
| Merom (2005),  “High income”  Before and after study (population-based cohort data) | **Name:** Walk to Work Day    **Location:** Australia    **Classification:** MM supporting events, "days" | **Channels:** Newspaper advertisements, community service announcements through the three major free-to-air television channels and radio stations nationally  **Messages:** Advertisement encouraged people to leave their cars at home and walk to work or take public transport and walk  **Additional components**: NA  **Duration:** Annual short-term campaign commenced on 15^th^ of September of 2003 and was promoted up until the event day, on 3^rd^ October of 2003. | **n:** 1100  **Age:** NR  **% female:** NR  **Target:** Working-age adults (18-65 years)  **Baseline PA level:** 15.3% and 16.2% of employed and unemployed adults were inactive (≤30 min/wk), respectively; 52% and 53% of employed and unemployed adults were sufficiently active (≥150 min/wk and 5 times/wk), respectively | Pre and post campaign; NSW Metropolitan vs Other Metropolitan | **Impact on PA:** PA behaviour Significant increase in trips that combined walking and public transport to work in both NSW (+9%; X^2^ (1) = 8.64, *p*<.005) and other metropolitan areas (+3.2%; X^2^ (1) = 4.76, *p*<.05). The proportion of employed adults who were inactive decreased (-4.0%; X^2^ (1) = 6.1, *p*< .05) and the proportion of employed adults sufficiently active increased (+5.4%; X^2^ (1) = 6.7, *p*< .005). There was no change in these proportions among participants who were not employed. |
| Wray (2005),  “High income”  Post-campaign only | **Name:** Walk Missouri  **Location:** St Joseph, Missouri, US  **Classification:** CWI with MMC | **Channels:** Billboard, newspaper, radio and poster advertisements  **Messages:** Promotion of the benefits of the health, social and pleasure benefits of walking, along with messages providing ideas on how to incorporate walking into a busy schedule  **Additional components**: Local community sponsored activities (e.g. scheduled walks and other wellness activities)  **Duration:** May-September 2003 (one-off) | **n:** 297  **Age:** 47  **% female:** 62  **Target:** Adults ≥18 years  **Baseline PA level:** NR | Unexposed vs exposed respondents; Low vs medium vs high exposure | **Impact on PA** ^c^**:** PA behaviour - % of people who walked at least 10 min at a time during usual wk unexposed vs exposed respondents (p value): 89% vs 88% (0.94), % of people who walked at least 10 min at a time during usual wk low vs medium vs high exposure (p value): 84% vs 90% vs 90% (0.93). |
| Brown (2006),  “High income”  Quasi-experimental | **Name:** 10,000 Steps Rockhampton  **Location:** Rockhampton, Queensland, Australia  **Classification:** CWI with MMC | **Channels:** Television, radio and printed media  **Messages:** Promotion of walking 10000 steps a day and the general health benefits of PA  **Additional components**: PA promotion materials and brief training in PA counselling for GPs; sale and loan pedometers and logbooks; encouragement of other health practitioners to promote PA to their clients; local environment changes (e.g. creating and repairing footpaths; promotion of responsible dog walking)  **Duration:** January-March 2002 (A dedicated print, radio, and TV media campaign was conducted in the first 3 months of the project, supported over the longer term by both paid and non-paid marketing efforts. Additional marketing, such as mailings by the local city council, newsletters, email, and other special events was used to maintain the 10,000 Steps message during the 2-year intervention period) (one-off) | **Surveys 2001 and 2003**  **n:** 2339-2478  **Age:** 18-29 (19.7%-21.4%), 30-44 (33.4%-35%), 45-59 (20.6%-26%), 60-plus (19.2%-24.4%)  **% female:** 50.2-53.4  **Target:** Adults ≥18 years  **Baseline PA level:** % of people who were categorised as “active” (defined as ≥150min of PA in at least 5 separate sessions in the previous week) was higher in Mackay (48.3%) than in Rockhampton (41.9% (OR=0.77, CI: 0.65, 0.93)) in 2001. | Mackay regional community | **Impact on PA** ^c^**:** PA behaviour – In 2003, the percentage of people who were “active” in Mackay decreased by 6.4% (95% CI:-10.6,-2.2) to 41.9%, while there was no change in Rockhampton (from 41.9% to 42.8%; difference=0.9%; 95% CI: -3.2, 5.0). |
| Reger-Nash (2006),  “High income”  Quasi-experimental | **Name:** BC Walks  **Location:** Broome County, New York, US  **Classification:** CWI with MMC | **Channels:** Television, radio and newspaper  **Messages:** Promotion of 30 minutes or more of daily moderate-intensity walking  **Additional components**: Community presentations; community programs (e.g. worksite walking programs and school walking programs)  **Duration:** 8 weeks one-off | **n:** 600  **Age:** 52.3  **% female:** 70  **Target:** Adults aged 40-65 years  **Baseline PA level:** NR | Chautauqua county | **Impact on PA** ^c^**:** PA behaviour - % of people who showed any gain in minutes in walking time Broome vs Chautauqua, adjusted OR (95% CI): 47% vs 36% 1.66 (1.14-2.44); % of people who gained at least 30 minutes in weekly walking time Broome vs Chautauqua, adjusted OR (95% CI): 41% vs 31% 1.56 (1.07-2.28); % of people who changed from nonactive to active walker Broome vs Chautauqua, adjusted OR (95% CI): 16% vs 11% 1.71 (0.99-2.95) |
| Stackpool (2006),  “High income”  Before and after study | **Name:** Make a move  **Location:** Sydney metropolitan area and central coast of NSW  **Classification:** CWI with MMC | **Channels:** Radio and local print media  **Messages:** Promotion of PA as a strategy to reduce risk of falls, information regarding the risk of falls. The idea that is never too late to start exercising was emphasised  **Additional components**: Promotional drink coasters and carry bags; kits to older people from CALD communities; community PA programs; training and upskilling of fitness leaders; strengthening partnerships with providers and a physiotherapy referral project  **Duration:** 2000-2003 (one-off) | **n:** 2815  **Age:** 48% were 65 years or older, 38% were aged between 50-64 years and 15% were younger than 50 years.  **% female:** 85  **Target:** Adults aged ≥65 years  **Baseline PA level:** Number of PA classes in Area Health Service (AHS)-supported exercise programs for older people – 428; Number of participants in AHS-supported exercise programs – 5,305 | Annual cross-sectional data from surveys conducted with hotline callers between 2001 and 2003, number of programs and program participants | **Impact on PA** ^c^**:** PA behaviour – 14% of hotline callers enrolled in ongoing classes. AHS-supported exercise programs for older people increased the number of classes by 19% (+81); and the number of participants in AHS-supported exercise programs increased by 16% (+867) |
| John-Leader (2008),  “High income”  Cross-sectional survey | **Name:** To be young at heart - Stay active Stay independent  **Location:** Rural NSW, Australia  **Classification:** mostly CWI with MM support | **Channels:** Television, newspapers, radio, poster, pamphlet, bus-back transit advertisements, internet  **Messages:** Promotion of active aging, independence and incidental activity  **Additional components**: Free-call number that provide information about PA; booklet of PA opportunities and venues  **Duration:** 18 months (one-off) | **n:** 639  **Age:** 91% aged 60-plus years and 57% were aged 70-plus years.  **% female:** 63  **Target:** Adults aged ≥50 years  **Baseline PA level:** NR | Intensive media coverage areas were compared to partial campaign delivery areas | **Impact on PA** ^c^**:** PA behaviour - % of people who became more active (self-reported): 7.7% (intensive media intensity) vs 4.6 (partial media intensity) |
| Peterson (2008),  “High income”  Before and after study | **Name:** "Get up and do something"  **Location:** Delaware, US  **Classification:** CWI with MMC | **Channels:** TV ads (n=2) and billboards (n=5) in high visibility areas across Delaware  **Messages:** The goal was to get the audience to consider being more active, to associate PA with things they already value, and to convey a negative association with common behaviours such as sitting around watching television, but in a nonthreatening, nonauthoritative, motivational manner  **Additional components**: NA  **Duration**: 6 weeks one-off (February - March 2004) | **n**: 3,782 (completed survey)  **Age**: 12-19 years old  **% female**: NR  **Target**: Adolescents aged 12-17 years (n=110,906)  **Baseline PA level:** NR | None | **Impact on PA:**^*^ PA behaviour - % of people who became more active (self-reported): 45.21 at 6 wks |
| Huhman (2010),  “High income”  Before and after study (5 annual surveys) | **Name:** VERB campaign  **Location:** United States  **Classification:** mostly MM | **Channels:** Television (mainly on cable channels popular with children aged 9 to 13 years), advertising in magazines targeted to this group (Sports illustrated for kids, game pro, ELLEgirl)  **Messages:** promotion of the benefits of PA (“it’s social, fun, and cool”), self-efficacy (“try new activities, you don’t have to be a pro to be active”), and social influences (many of your peers are having fun being active”)  **Additional components**: School programs; vans with bright VERB logos canvassed US communities; website; environmental changes (e.g. setting up play areas communities’ recreational centres, camps, etc)  **Duration:** June 2002-September 2006 (one-off) | **n:** 5 waves of data collection –3114, 2729, 2256, 1946 and 1623 parent child dyads in waves 1, 2, 3, 4 and 5 respectively  **Age:** NR  **% female:** NR  **Target:** Children 9-13 years  **Baseline PA level:** NR | Different frequencies of exposure between 2003 and 2006 | **Impact on PA:** PA behaviour – Higher percentages of children were physically active (self-reported) the day before the survey the more they saw the campaign, varying from 62.4% with no campaign exposure to 68.4% for those who saw it every day (*y=*0.09; *p*<.05). Children’s reports of free-time PA showed significant associations with campaign exposure in 2004 and 2005, but not in 2006. Organized PA was not associated with campaign exposure |
| Huberty (2012),  “High income”  Before and after study | **Name:** Activate Omaha  **Location:** Omaha, Nebraska, US  **Classification:** mostly CWI with MM support | **Channels:** Television, radio and billboards  **Messages:** NR  **Additional components**: Worksite toolkits  **Duration:** 2005-2007 | **n:** 252 telephone interviews in 2006 and 329 telephone interviews in 2008  **Age:** NR  **% female:** NR  **Target:** Families and workers  **Baseline PA level:** NR | Pre and post campaign | **Impact on PA:** PA behaviour – 39% of respondents reported being “a little more active” and “much more active” (self-reported) in 2008 than in the previous year. PA increased by 20% from 2005 to 2007 (self-reported) |
| Leavy (2013),  “High income”  Before and after study (3 annual surveys) | **Name:** Find Thirty every day  **Location:** Western Australia  **Classification:** mostly MM | **Channels:** Television, radio, print advertising, billboards, website, online resources  **Messages:** Advertisements encouraged people to "find a way" to engage in PA while emphasising its health benefits (e.g. “Finding better health", "finding stronger bones", "finding a way to keep cholesterol down")  **Additional components**: Community-wide programs  **Duration:** 2007-2010 | **n:** 2847  **Age:** 20-34 years (619), 35-45 years (1109), 46-54 years (1119)  **% female:** 49.2  **Target:** Adults aged 20-54 years  **Baseline PA level:** Median Minutes (IQR) – total walking/wk 90 (40-240); Moderate PA 0 (0-0); Vigorous PA 0 (0-120); Total PA/wk 190 (80-420) | Three different phases of the campaign | **Impact on PA** ^c^**:** PA behaviour – When comparing media minutes from Phases 1 and 2, significant increases were seen in “Total walking per wk” (30 minutes, *p*=0.47), “Total vigorous PA per wk” (20 minutes, *p*=0.22) and “Total PA per wk” (50 minutes, *p*=0.004). Only one significant change was observed between Phases 2 and 3: “Total walking per wk” (*p*=.034). Total moderate PA per week remained unchanged at 0 minutes for all three time points |
| Clark (2015),  “High income”  Before and after study | **Name:** Happy trails  **Location:** Las Vegas, Nevada, US  **Classification:** MM supporting events, "days" | **Channels:** Print, online and radio ads, billboards and signage on gas pumps  **Messages:** Promotion of trail use  **Additional components**: NA  **Duration:** 8 weeks one-off (February-March 2012) | Infrared trail counters were used to determine whether the usage of trails had increased. Demographic data is not available.  **Target:** Women ages 18-54 years and parents of children ages 8-15 years  **Baseline PA level:** 3.91 users per hour | Pre and post campaign | **Impact on PA:** PA behaviour – Trail usage increased by 52.17% (from 3.91 to 5.95 users per hour). Usage on 7 of the trails (out of 10) increased significantly |
| Kite (2018),  “High income”  Before and after study (3 surveys) | **Name:** Make healthy normal  **Location:** NSW, Australia  **Classification:** mostly MM | **Channels:** Television, out-of-home (billboards), online advertising, public relations, a website and social media  **Messages:** The messages were focused on changing social norms that normalise unhealthy behaviours  **Additional components**: Community events  **Duration:** June 2015-June 2016 | **Waves 1-3**  **n:** 1113-2259  **Age:** 18-39 years (23.2%-34.2%), 40 and over (65.8%-76.8%  **% female:** 52.5-54.6  **Target:** Adults over 18 years, particularly those who were overweight or obese and/or at risk of developing chronic disease  **Baseline PA level:** 59.6 of respondents had tried to increase PA in the last six months and 61.8% were meeting PA recommendations (30 min of MVPA/day) at wave 1 | Pre, during and post campaign | **Impact on PA** ^c^**:** PA behaviour - % of participants who tried to increase PA in the last six months decreased at wave 2 (53.3%, adjusted OR 0.86, 95% CI 0.75-0.98, p=0.024) and wave 3 (52.2%, OR 0.85, 95% CI 0.74-0.97, *p*=0.014). The proportion of participants meeting PA recommendations did not significantly change at waves 2 (60.9%) and 3 (59.6) |
| Kite (2020),  “High income”  Before and after study (3 surveys) | **Name:** Make healthy normal  **Location:** NSW, Australia  **Classification:** mostly MM | **Channels:** Television, radio, out-of-home (billboards, bus sides, train stations) displays, social media, website  **Messages:** Promotion of active travel, normalization of unhealthy behaviours and encouragement of individual behaviour change for PA  **Additional components**: NA  **Duration:** May 2017-April 2018 | **Surveys 1-3**  **n:** 1214-1531  **Age:** 18-34 years (31-38%), 35-54 years (62-69%)  **% female:** NR    **Target:** In Phase 2 the campaign the campaign narrowed its focus to men aged 35–54 years and families with children aged 5–12 years  **Baseline PA level:** NR | Cross-sectional survey data from three time points: baseline survey, after peak of Phase 2 and after conclusion of the television advertisement | **Impact on PA** ^c^**:** PA behaviour – Participants who recognised the campaign were more likely to be meeting PA guidelines, i.e. 2.5-5hrs of moderate intensity PA and/or 1.25-2.5 hours of vigorous intensity PA (adjusted OR 1.30 (1.10, 1.53). |
| Berry (2020),  “High income”  Post-campaign cross-sectional survey | **Name:** ParticipACTION’s 150 Play List  **Location:** Canada  **Classification:** mostly MM | **Channels:** Television, billboards, radio and social media  **Messages:** Key messages were: 1) being active is ‘part of who we are as Canadians’ and 2) PA is enjoyable  **Additional components**: Community events  **Duration:** 6th Jan 2017 until the end of 2017 | **n:** 1185  **Age:** 38.7  **% female:** 50.4  **Target:** Individuals ≥ 13 years  **Baseline PA level:** NR | None | **Impact on PA** ^c^**:** PA behaviour – 24.5% of the respondents who were aware of the 150 Play List responded that they took steps to be healthier as a result of it; 8.6% took steps to be less sedentary and 7.5% reported participating in more PA (self-reported) |
| King (2013),  “High income”  Before and after study (2 surveys) | **Name:** Measure-up  **Location:** Australia  **Classification:** mostly MM | **Channels:** Television, radio, magazines, online advertising and out-of-home  **Messages:** Promotion of healthy diet and PA as strategies prevent chronic diseases, along with messages which emphasised waist circumference as an indicator of unhealthy lifestyle that increases chronic disease risk  **Additional components**: Community activities  **Duration:** October 2008-April 2009 | **n:** 1006  **Age:** 18-44 years (60%), 45-65 (40%)  **% female:** 50.6  **Target:** Australian adults, with a particular focus on those who were aged 25 and 50 years and had children. The secondary target audience was adults aged 45-60 years  **Baseline PA antecedents level:** 50.3% of participants knew about the recommended 30 min of PA/day at baseline | Pre and post campaign | **Impact on PA** ^c^**:** PA antecedents – Knowledge about the recommended minutes of PA/day increased from 50.3% to 56.5%, *p*=.006. Perceptions about the importance of doing 30 min of PA to prevent chronic disease and confidence about increasing PA levels to improve health did not change post-campaign |
| Buchthal (2011),  “High income”  Post-campaign cross-sectional survey | **Name:** Step-up Hawaii (one arm of the campaign Start.Living.Healthy)    **Location:** Hawaii, US    **Classification:** mostly MM | **Channels:** Television, radio, mall posters, newspapers, a website, media materials placed in public venues  **Messages:** Promotion of the benefits of walking (e.g. ‘‘People should walk 30 minutes a day’’, ‘‘Walking gives you energy’’, ‘‘There is benefit in walking only ten minutes’’)  **Additional components**: Community activities and events  **Duration:** April-June 2007 | **n:** 3607  **Age:** 8-34 (12.2%), 35-54 (36.9%), 55-74 (39.1%), 75+ (10.5%), No response (1.3%)  **% female:** 65.3  **Target:** Adults aged 35-55 years  **Baseline campaign awareness level:** NR | People from different ethnicities, levels of education and income | **Impact on PA:** Campaign awareness was significantly lower among individuals with lower income, less than a high school education, men, Caucasians and those ≥75 years. Campaign awareness was higher among Native Hawaiians and those aged 35-54 years. Both females and those who have higher levels of education were more likely to believe and trust the campaign messages |
| Bell (2013),  “High income”  Quasi-experimental | **Name:** Good for Kids  **Location:** Hunter New England (HNE) region, NSW, Australia  **Classification:** mostly CWI with MM support | **Channels:** Television, radio, print media (emails, posters, newsletters)  **Messages:** Advertisements emphasised that children need at least one hour of PA every day and that children should not play sitting down for more than 2 hours a day  **Additional components**: Community programs  **Duration:** 2008 (PA promotion campaign was in 2008, but the program started in 2005 and ended in 2010) | **Surveys 1- 4**  **n:** 315-405  **Age:** <20 years (0-1.2%), 20-39 years (50.1%-53.2%), 40 years or older (46.4-51.1%)  **% female:** 80.3-86.1  **Target:** Children up to 12 years, parents and carers aged 25-54 years  **Baseline campaign awareness level**: 27% in HNE and 20% in NSW | Pre and post campaign, NSW | **Impact on PA** ^c^**:** Campaign awareness – The PA campaign achieved 60% of awareness in HNE and 21% in NSW. HNE participants were significantly (*p*<0.001) more likely to identify the main PA messages immediately post-campaign compared to the rest of NSW participants |

^a^ Impact on physical activity indicates the effect of the intervention on physical activity. Economic outcome indicates the health outcome used in the economic evaluation.

^b^ In cases where multiple interventions were investigated, we only presented the relevant information for physical activity mass media campaign programmes

^c^ Where multiple physical activity effectiveness outcomes were reported in the paper, only one outcome was reported in the table based on the following hierarchy: i) physical activity behaviour, ii) physical activity antecedents (e.g. intention, knowledge, attitudes, efficacy), iii) campaign awareness, recognition, campaign message understanding.

CWI: community-wide intervention, DALY: disability-adjusted life year, GRP: Gross rating points, IQR: interquartile range, MET: metabolic equivalents, MM: mass media, MMC: mass media campaign, MVPA: Moderate to vigorous physical activity; NA: not applicable, NR: not reported, NSW: New South Wales, OECD SHPeP-NCD: Organization for Economic Co-operation and Development Strategic Public Planning for Noncommunicable diseases, OR: odds ratio, PA: physical activity, QALY: quality adjusted life year, SD: standard deviation, UK: United Kingdom, US: United States, wk: week, wks: weeks.

# Appendix table 2. Mass media campaigns by target population

| **Target population** | **Author (Year), Campaign name** |
| --- | --- |
| **Children** |  |
| 9-13 years | Huhman (2010), VERB campaign |
| **Adolescents** |  |
| 12-17 years | Peterson (2008), "Get up and do something" |
| **Adults** |  |
| All adults, but particularly the middle aged and males 30-54 years | Bauman (2003), Push Play |
| 18-65 years | Merom (2005), Walk to Work Day |
| 40-65 years | Reger-Nash (2006), BC Walks |
| 25-64 years | **Roux (2008), Wheeling Walks** |
| Adults (age not specified) | **Cobiac (2009), Exercise, you only have to take it regularly not seriously** |
| 35-55 years | Buchthal (2011), Step-up Hawaii (one arm of the campaign Start.Living.Healthy) |
| 20-54 years | Leavy (2013), Find Third every day |
| All adults, but particularly adults aged 25-50 years with children | King (2013), Measure-up |
| Women aged 18-54 years and parents of children ages 8-15 years | Clark (2015), Happy trails |
| Adults (age not specified) | **Goryakin (2019), NR (Hypothetical campaign)** |
| Men aged 35-54 years and families with children aged 5-12 years | Kite (2020), Make healthy normal |
| **Adults and older people** |  |
| 16-74 years, but particularly women aged 16-24 years, middle aged men 45-55 years; men and women aged >50 | Hillsdon (2001), ACTIVE for LIFE |
| ≥18 years | Wray (2005), Walk Missouri |
| ≥18 years | Brown (2006), 10,000 Steps Rockhampton |
| ≥50 years | John-Leader (2008), To be young at heart - Stay active Stay independent |
| 25-75 years | **De Smedt (2011), 10,000 Steps Ghent** |
| ≥18 years | Kite (2018) |
| **Lifespan** |  |
| Students, workers and elderly | Mahecha Matsudo (2003), Agita São Paulo |
| Families and workers | Huberty (2012), Activate Omaha |
| Children up to 12 years of age, parents and carers | Bell (2013), Good for kids |
| ≥13 years | Berry (2020), ParticipACTION’s 150 Play List |
| 15-79 years | **Mizdrak (2020), NR (Hypothetical campaign**) |
| **Older people** |  |
| ≥65 years | Stackpool (2006), Make a move |

Bold indicates the model-based analysis.

# Appendix table 3. Mass media campaigns by geographical location

| **Geographical area** | **Author (Year), Campaign name** |
| --- | --- |
| European Region | |
| United Kingdom | Hillsdon (2001), ACTIVE for LIFE |
| Ghent, Belgium | **De Smedt (2011), 10,000 Steps Ghent** |
| Italy | **Goryakin (2019), NR (Hypothetical campaign)** |
| Region of the Americas | |
| São Paulo, Brazil | Mahecha Matsudo (2003), Agita São Paulo |
| St Joseph, Missouri, United States | Wray (2005), Walk Missouri |
| Broome County, New York, United States | Reger-Nash (2006), BC Walks |
| Delaware, United States | Peterson (2008), Get up and do something |
| Single community in West Virginia, United States | **Roux (2008), Wheeling Walks** |
| United States | Huhman (2010), VERB campaign |
| Hawaii, United States | Buchthal (2011), Step-up Hawaii (one arm of the campaign Start.Living.Healthy) |
| Omaha, Nebraska, United States | Huberty (2012), Activate Omaha |
| Las Vegas, Nevada, United States | Clark (2015), Happy trails |
| Canada | Berry (2020), ParticipACTION’s 150 Play List |
| Western Pacific Region | |
| New Zealand | Bauman (2003), Push Play |
| New Zealand | **Mizdrak (2020), NR (Hypothetical campaign promoting physical activity apps)** |
| Australia | Merom (2005), Walk to Work Day |
| Rockhampton, Queensland, Australia | Brown (2006), 10,000 Steps Rockhampton |
| Sydney metropolitan area and central coast of NSW, Australia | Stackpool (2006), Make a move |
| Rural New South Wales, Australia | John-Leader (2008), To be young at heart - Stay active Stay independent |
| Australia | **Cobiac (2009), Exercise, you only have to take it regularly not seriously** |
| Western Australia | Leavy (2013), Find Third every day |
| Hunter New England region, New South Wales, Australia | Bell (2013), Good for kids |
| Australia | King (2013), Measure-up |
| New South Wales, Australia | Kite (2018), Kite (2020), Make healthy normal |

Bold indicates the model-based analysis.

# Appendix table 4. Description of the approach to the model-based analyses of economic evaluations of physical activity mass media campaigns

| **Author (Year) / Campaign** | **PA impact estimate source and assumptions** | **Future health benefits of PA** | **Parameters used** | **Calibration of input for local data** |
| --- | --- | --- | --- | --- |
| Roux (2008),  *“Wheeling walks”* | Estimated by authors based on effectiveness study (Reger, 2002). The study was selected by the Task Force on Community Preventive Services (the Task Force). Assumptions:  -Impact of an intervention declined after the intervention had ended (50% decline in year 2)  -In Year 2 the general decline in physical activity that occurs with age was modelled | Future health benefits of PA: CHD, ischemic stroke, type 2 diabetes, breast cancer, and colorectal cancer  Did not consider obesity as a disease outcome in this model  Health states: four activity levels among the well, the five physical activity–related diseases (coronary heart disease, ischemic stroke, type 2 diabetes, breast cancer, colorectal cancer), and death. | CDC MOVE physical activity policy model   - Disease-specific mortality by physical activity level, age group, and gender (Table 1) - Age, gender and physical activity level distribution of the initial population (Table 1) - Physical activity levels of US population cohort (Table 2) - Transition probabilities (Table 1) - Disease incidence (Table 1) - RR of disease by physical activity level (Table 1) - Intervention costs (calculated by authors based on effectiveness study) - Healthcare costs (Table 3) - Participants time cost (Reference 53) | Overall parameters used were relevant to US population |
| Cobiac (2009),  *“Exercise, you only have to take it regularly not seriously”* | Estimated by authors based on the effectiveness study (Bauman, 2001). Study was selected in a literature review conducted by the authors (methods described in Text S1)  Transformed the net intervention effect from hours/week to MET-min/week (Details in text S1). Sustainability of intervention health effects over time: intervention effects on PA are sustained for the first year, but decay exponentially at a rate of 50% per annum thereafter | Future health benefits of PA: ischaemic heart disease, ischaemic stroke, type 2 diabetes, breast cancer and colon cancer  Health states: healthy, diseased, dead from the disease, dead for other causes (Text S2) | - Distribution: Text S3. - Relative risk of disease due to physical inactivity: Table 1, Text S2 - Physical activity prevalence: Text S2 - Intervention costs: Text S1 - Cost of NSW campaign* scaled to population: Table 4, Text S1 - Disease costs (cost per incident case or prevalent case): Table 3, Text S2 - Life table / DALY calculation: Text S2 | Overall parameters used were relevant to Australian population. |
| De Smedt (2011),  *“10,000 Steps Ghent”* | Effectiveness study (De Cocker 2007). Base case analysis assumed a life-long programme with a life-long intervention effect. | Future health benefits of PA: Diabetes, CHD, stroke, colon cancer  Health states: healthy, diabetes mellitus type 2, CHD (first year), CHD follow up, stroke (first year), stroke follow up, colon cancer (first year), colon cancer follow-up and dying | - State transition probabilities (Table 1) - Relative risk reductions for diabetes, CHD, stroke, colon cancer (Table 3) - Cost data (in euros): intervention costs (first year and second-fifth year), healthcare costs for each health state (Table 3) - Data distribution (Table 3) - Utilities for the eight health states (Table 3) | Overall parameters used were relevant for Ghent, Belgium |
| Goryakin (2019),  *“Generic primary physical activity campaign”* | Median effectiveness of previous MMC reported in 12 papers included in a literature review^[[12]](#footnote-13)^  Adjustment of effects for campaign duration: 60% increase after 1 month, drop to 30% by the end of the 1s year, drop to 0 after 2 more years (unclear how this was calculated). For the less inactive individuals, the probability of moving into the new physical activity category is higher | Future health benefits of PA  Direct effect: diabetes, myocardial infarction, ischemic stroke, colorectal and breast cancers, depression  Indirect effect: diseases linked to overweight and diabetes, including, haemorrhagic stroke, several cancers, dementia, back pain, osteoarthritis, gout, atrial fibrillation | OECD SPHeP-NCD model was used and the following parameters considered:   - Effectiveness of interventions at the individual level (from existing published meta-analyses in three modelled policies) - Effectiveness of the interventions over time - Intervention coverage, including description of eligible populations, as well as their exposure - Implementation cost.   Specific parameters were not reported and reference to previous publication provided^[[13]](#footnote-14),^ ^[[14]](#footnote-15)^ | Unclear. The impact of the intervention on 36 countries is presented in another report, but the parameter used are not reported^[[15]](#footnote-16)^ |
| Mizdrak (2020),  *“Hypothetical campaign to promote use of PA apps.”* | Calculated by authors based on a systematic review of interventions that used app or pedometers. Transformed the number of steps/day to MVPA-METmin/week.  In the absence of an effectiveness study investigating the effects of such a campaign, several assumptions were made about change in awareness, download of app, use of app, adherence to app and impact on PA (Details provided in the technical report)^[[16]](#footnote-17)^ | Future health benefits of PA: Coronary heart disease (CHD), stroke, type 2 diabetes, colorectal cancer, breast cancer (females only) | - Impact of the mass media campaign on: campaign awareness, download of app, use of app, adherence to physical activity app, increase in PA levels: Table 1 - Total cost of the campaign: Table 1 - Health costs: report^[[17]](#footnote-18)^ - Relative risk for disease incidence: report^[[18]](#footnote-19)^ - Utility: NR | To some extent. Some of the data were from other countries (e.g. UK, US) |

CHD: coronary heart disease, DALY: disability-adjusted life year, MET-min: Metabolic equivalent per minute, MMC: Mass media campaigns, MVPA: Moderate-to-vigorous Physical Activity, PA: physical activity, S: Supplementary file, NSW: New South Wales, OECD SPHeP-NCD: Organization for Economic Co-operation and Development Strategic Public Planning for Noncommunicable diseases, UK: United Kingdom, US: United States, RR: relative risk.

# Appendix table 5. Main economic evaluation findings of model-based analyses of physical activity mass media campaigns

| **Author (Year), Campaign** | **Perspective / Currency (year)** | **Time horizon / discounting** | **Incremental cost-effectiveness ratio – Reported currency** | **Incremental cost-effectiveness ratio – US 2020** | **Sensitivity analysis** |
| --- | --- | --- | --- | --- | --- |
| Roux (2008),  Wheeling walks | Societal  USD (2003) | 40 years  Costs and QALY: 3% | ICER: $22,654/LY gained  ICER: $14,286/QALY gained | ICER: $31,872/LY gained  ICER: $20,099/QALY gained | One-way, two-way, and probabilistic sensitivity (Monte Carlo) analyses  Intervention parameters: Repeating the intervention once after 20 years had a small effect on cost-effectiveness (results not presented). Similarly, varying the dissipation of the effect size of the interventions had a marginal impact  Time horizon: Shortening the analytic time-horizon from 40 to 30, 20, or 10 years influenced cost-effectiveness substantially  Probabilistic: At a threshold of $75,000/QALY there was a 100% probability of the intervention being cost-effective |
| Cobiac (2009),  Exercise, you only have to take it regularly not seriously | Health sector perspective  AUD (2003) | Lifetime of the Australian population (baseline year of 2003)  Costs and QALY: 3% | Cost Offsets (total): -$430 million (95% UI -$800 to -$130 million)  DALYs averted (total): 23,000 (7,600 to 40,000).  Dominant (100% probability of being cost-saving) | Cost Offsets (total): -$931 million (-$1,176 to -$281 million) | One-way and probabilistic (Monte Carlo) sensitivity analysis  Sustainability of intervention effects over time: Sensitivity was evaluated by varying decay rates of PA effects between 0% (lifelong behaviour change) and 100% (behaviour change reversed after the first year). The intervention remained dominant in all the decay scenarios  Probabilistic: 100% of the intervention being more effective and less costly |
| De Smedt (2011),  10 000 Steps Ghent | Public payer perspective  Euro (2009) | 20 years (cycle length of 1 year)  Costs: 3% and QALY: 1.5% | Incremental costs (average): -$576 (men), -$427 (women)  Incremental QALY (average): 0.16 (men). 0.11 (women)  Intervention was cost-saving (more effective and less costly) | Incremental costs (average): -$521.51 (men), -$386.61 (women) | One-way and probabilistic (Monte Carlo) sensitivity analyses  Utility values and intervention effects: small impact on the difference in QALY between intervention and control groups  Sustainability of intervention effects over time:  1) 5-year intervention effects: intervention remained dominant  2) 1-year intervention effects: substantial decrease in QALY gain and cost savings, however the overall result remained favourable (estimates only reported in a figure)  Relative risk reductions: Most important impact on results. More pronounced change in QALY and cost, but the intervention remained dominant  Intervention costs and cost of colon cancer: Only a minor impact on the total change in cost  Probabilistic: 100% chance of the intervention being more effective and less costly |
| Goryakin (2019) ^a^,  Hypothetical primary PA campaign | Health system perspective  Euro (2015) | 31 years  Costs and QALY: 3% | Year 2025: 147,346/DALY  Year 2037: 47,985/DALY  Year 2050: 28,163/DALY | Year 2025: $113,603/DALY  Year 2037: $36,996/DALY  Year 2050: $21,713/DALY  (provided by the authors)  The impact of the intervention on 36 countries^[[19]](#footnote-20)^ is presented in another report | NR. The references describing the model describe several methods for modelling uncertainty^[[20]](#footnote-21),^^[[21]](#footnote-22)^, including simulation, baseline and intervention uncertainty. However the results of these analyses are not reported |
| Mizdrak (2020),  Hypothetical campaign to promote use of PA apps. | Health sector perspective  NZD (2011) | Lifetime of the New Zealand population in a baseline year of 2011 | ICER: $81,000/QALY gained (95% UI 17,000-345,000) | ICER: $130,740/QALY gained (95% UI 18,989 to 385,367) | One-way and probabilistic (Monte Carlo)  -Equity adjustment to background: Health gains for Māori increased  Intervention parameters: uncertainty in health gains was driven by uncertainty in the app use parameter. Uncertainty in health system cost impacts was driven by uncertainty in the intervention cost parameter. Uncertainty in app use was the greatest contributor to uncertainty in the ICER, but this was closely followed by uncertainty around other intervention parameters  Scenario analysis 1: narrowing the target population to 40-79 years: small impact on ICER  Scenario analysis 2: assumptions that intervention impact would be maintained for 5 years following the intervention: substantial impact on ICER (2,000/QALY gained)  -Discount rate (0 to 6%): expected impact on health gain |

^a^In cases where multiple interventions were investigated, we are only presented the relevant information for mass media campaign programmes. App: Applications, AUD: Australian dollar, DALY: disability-adjusted life year, ICER: Incremental cost-effectiveness ration, LY: life-year, mi: million, NR: not reported, NZD: New Zealand dollar, PA: physical activity, QALY: QALY: quality adjusted life year, USD: United States dollar

# Appendix table 6. Description of cost items and valuation sources used in the economic evaluations of physical activity mass media campaign

| **Author (year), Campaign** | **Identification cost categories** | **Valuation sources** | **Total costs**^a^  **Currency (year)** |
| --- | --- | --- | --- |
| Roux (2008),  *“Wheeling walks”* | Intervention costs: NR | Effectiveness study - communication with authors/additional publications | Average cost  -Intervention: $195,713  -No intervention: $195,013  USD (2003) |
|  | Patients out-of-pocket expenses: NR | Estimated by authors |  |
|  | Participant's time: NR | Age and gender specific wage from literature |  |
|  | Direct medical costs: NR | Estimated based on national medical claim data |  |
|  | Health care cost: -$440 (-$820 to -$140) – savings | National data – Australian Institute of Health and Welfare (2006) Health expenditure Australia. |  |
| Cobiac (2009),  *“Exercise, you only have to take it regularly not seriously”* | Intervention costs: $13.3 (SD 1.33) million | Effectiveness study and similar campaign conducted in the same state | Incremental costs (intervention - control) -$430 mi (-$800 to -$130) |
|  | Health care costs | Australian Institute of Health and Welfare Disease Costs and Impacts Study 2001 | AUD (2003) |
| De Smedt (2011),  *“10,000 Steps Ghent”* | Intervention costs (cost/person): 1^st^ year Cost/person (first year): $3.51 (1^st^ year); $0.23 (2nd-5th year) | Estimated by authors based on effectiveness paper (assumption, details not provided) | Cost/person  -Intervention group: $2,963 (men) $2,454 (women)  -Control group: $3,539 (men), $2,881 (women)  Euro (2009) |
|  | Direct medical costs: NR | Literature |  |
| Goryakin (2019),  *“Hypothetical mass media campaign”* | Intervention costs: $1.58 (annual cost per capita) | Broadly based on the WHO-Choice methodology (Details not provided) | NR  Euro (2015) |
|  | Healthcare costs (incremental): - 396 million over 31 years) | OECD SPHeP-NCD model (prevalence based direct cost estimates as an input) |  |
| Mizdrak (2020),  “*Hypothetical campaign to promote use of PA apps.”* | Intervention costs: $2,883,000 | Estimated based on previous model analysis that used data from a campaign encouraging smokers to quit (Quitline). Details provided in a report^[[22]](#footnote-23)^ | $2,883,000  NZD 2011 |
|  | Health system costs (disease specific costs): net cost $2.2 million (US $1,625,000; 95% UI 1.02 million-3.5 million). | Details provided in a report^[[23]](#footnote-24)^ |  |

^a^Total costs refer to all costs included in the cost-effectiveness analysis.

AUD: Australian dollar, AIHW: Australian Institute of Health and Welfare, NR: Not reported, NZ: New Zealand, NZD: New Zealand dollar, PA: Physical activity, OECD SHPeP-NCD: Organization for Economic Co-operation and Development Strategic Public Planning for Noncommunicable diseases, UI: Uncertainty interval, US: United States, USD: United States Dollar

# Appendix table 7. Quality of economic evaluation of physical activity mass media campaigns according to CHEC-List

| **Author, year** | **Item** | | | | | | | | | | | | | | | | | | | |  |
| --- | --- | --- | --- | --- | --- | --- | --- | --- | --- | --- | --- | --- | --- | --- | --- | --- | --- | --- | --- | --- | --- |
|  | 1 | 2 | 3 | 4 | 5 | 6 | 7 | 8 | 9 | 10 | 11 | 12 | 13 | 14 | 15 | 16 | 17 | 18 | 19 | 20 | **Score out of 20 (%)** |
| Roux, 2008 | Y | Y | Y | Y | Y | Y | Y | Y | Y | Y | N | N | Y | Y | Y | N | N | Y | Y | Y | 16 (80) |
| Cobiac, 2009 | Y | Y | Y | Y | N | Y | N | Y | Y | Y | Y | Y | Y | Y | Y | N | N | Y | Y | N | 15 (75) |
| De Smedt, 2011 | Y | Y | Y | Y | N | Y | N | Y | Y | Y | N | N | Y | Y | Y | Y | Y | N | Y | N | 14 (70) |
| Goryakin, 2019 | Y | Y | Y | Y | Y | Y | N | Y | Y | N | Y | N | Y | Y | Y | N | N | N | N | N | 12 (60) |
| Mizdrak, 2020 | Y | Y | Y | Y | N | Y | N | Y | Y | N | N | N | N | Y | Y | N | N | N | Y | Y | 11 (55) |

N: No, Y: Yes.

Item 1: Is the study population clearly described?; 2: Are competing alternatives clearly described?; 3: Is a well-defined research question posed in answerable form? ; 4: Is the economic study design appropriate to the stated objective? ; 5: Are the structural assumptions and the validation methods of the model properly reported (models)?; 6: Is the chosen time horizon appropriate in order to include relevant costs and consequences?; 7: Is the actual perspective chosen appropriate?; 8: Are all important and relevant costs for each alternative identified?; 9:Are all costs measured appropriately in physical units?; 10: Are costs valued appropriately?; 11: Are all important and relevant outcomes for each alternative identified?; 12: Are all outcomes measured appropriately?; 13: Are outcomes valued appropriately?; 14: Is an appropriate incremental analysis of costs and outcomes of alternatives performed?; 15: Are all future costs and outcomes discounted appropriately?; 16: Are all important variables whose values are uncertain appropriately subjected to sensitivity analysis?; 17: Do the conclusions follow from the data reported?; 18: Does the study discuss the generalizability of the results to other settings and patient/client groups?; 19: Does the article/ report indicate that there is no potential conflict of interest of study researcher(s) and funder(s)?; 20: Are ethical and distributional issues discussed appropriately?

# Appendix table 8. Expanded CHEC-list - Additional questions on the quality of economic evaluations of physical activity mass media campaigns

| **Author, year** | **Item** | | |
| --- | --- | --- | --- |
|  | 1. Was the effectiveness measure used appropriate? Did the study where the effectiveness estimate was removed from investigate similar population and mass media campaign with the same characteristics to the one being modeled? | 2. Did the model appropriately consider attenuation of physical activity impact post-campaign? Was any evidence used to support effect of the intervention beyond the trial duration? | Did the study report intermediate measures or use a “stepped approach” to report the results of each step of the model to allow understanding of the impact of each step on the overall results? |
| Roux, 2008 | N | N | N |
| Cobiac, 2009 | Y | N | N |
| De Smedt, 2011 | Y | y | N |
| Goryakin, 2019 | N | N | N |
| Mizdrak, 2020 | N | N | N |

N: No, NA: not applicable, Y: Yes.

Additional details for each of these items as well as additional information are provided in Appendix table 6.

#

# Appendix table 9. Quality of costing studies according to a modified version of CHEC-list

| **Author, year** | **Item** | | | | | | | | | | | | | | |  |
| --- | --- | --- | --- | --- | --- | --- | --- | --- | --- | --- | --- | --- | --- | --- | --- | --- |
|  | 1 | 2 | 3 | 4 | 5 | 6 | 7 | 8 | 9 | 10 | 11 | 12 | 13 | 14 | 15 | **Score out of 15 (%)** |
| Hillsdon, 2001 | Y | Y | Y | N | N | N | N | N | N | N | N | Y | N | Y | N | 5 (33) |
| Bauman, 2003 | Y | Y | Y | N | N | N | N | N | N | N | N | Y | N | N | Y | 5 (33) |
| Mahecha Matsudo, 2003 | Y | N | N | N | N | N | N | N | N | Y | N | Y | Y | N | Y | 5 (33) |
| Merom, 2005 | Y | Y | Y | N | N | N | N | N | N | Y | N | Y | N | N | Y | 6 (40) |
| Wray, 2005 | Y | N | Y | N | N | N | N | Y | N | Y | N | Y | Y | N | N | 6 (40) |
| Brown, 2006 | Y | Y | Y | N | N | N | N | Y | N | N | N | Y | N | N | N | 5 (33) |
| Reger-Nash, 2006 | Y | Y | Y | N | N | N | N | Y | N | Y | N | Y | N | N | N | 6 (40) |
| Stackpool, 2006 | Y | Y | Y | N | N | N | N | N | N | N | N | Y | N | N | Y | 5 (33) |
| John-Leader, 2008 | Y | Y | Y | N | N | N | N | Y | N | N | N | Y | N | N | N | 5 (33) |
| Peterson, 2008 | Y | NA | Y | N | N | N | N | Y | N | Y | N | Y | N | N | N | 5 (33) |
| Huhman, 2010 | Y | Y | Y | N | N | N | N | N | N | N | N | Y | N | N | N | 4 (27) |
| Buchthal, 2011 (2011) | Y | N | Y | N | N | N | N | Y | N | Y | N | Y | N | N | Y | 6 (40) |
| Huberty, 2012 | N | Y | Y | N | N | N | N | N | N | N | N | Y | N | N | N | 3 (20) |
| Bell, 2013 | Y | Y | Y | N | N | N | N | N | N | N | N | Y | N | Y | Y | 6 (40) |
| King, 2013 | Y | Y | Y | N | N | N | N | N | N | Y | N | Y | N | Y | Y | 7 (47) |
| Leavy, 2013 | Y | Y | Y | N | N | N | N | N | N | N | N | Y | N | Y | Y | 6 (40) |
| Clark, 2015 | Y | Y | Y | N | N | N | N | N | N | Y | N | Y | N | Y | N | 6 (40) |
| Kite, 2018 | Y | Y | Y | N | N | N | N | N | N | Y | N | Y | Y | Y | N | 7 (47) |
| Kite, 2020 | Y | Y | Y | N | N | N | N | Y | N | Y | N | Y | Y | Y | Y | 9 (60) |
| Berry, 2020 | Y | N | yes | N | N | N | Y | N | N | Y | N | Y | N | Y | N | 6 (40) |

N: No, NA: not applicable, Y: Yes.

Item 1: Is the study population clearly described?; 2: Are competing alternatives clearly described?; 3: Is a well-defined research question posed in answerable form?; 4: Is the actual perspective chosen appropriate?; 5: Are all important and relevant costs for each alternative identified?; 6: Are all costs measured appropriately in physical units?; 7: Are costs valued appropriately?; 8: Does the article provide a breakdown of the costs and report costs for each item separately?; 9: Is an incremental analysis of costs of alternatives performed?; 10: Are all future costs discounted appropriately?; 11: Are all important variables, whose values are uncertain, appropriately subjected to sensitivity analysis?; 12: Do conclusions follow from the data reported?; 13: Does the study discuss the generalizability of the results to other settings and patient/client groups?; 14: Does the article indicate that there is no potential conflict of interest of study researcher(s) and funder(s)?; 15: Are ethical and distributional issues discussed appropriately?

#

# Appendix table 10. Intervention costs description

| **Author (Year), Income** | **Campaign name / Classification** | **Duration** | **Cost items measured / Total costs (reported currency, year)** | **Estimated costs in US 2020** | **Estimated cost/ week (US 2020)** |
| --- | --- | --- | --- | --- | --- |
| **Model-based** |  |  |  |  |  |
| **Mostly MM** |  |  |  |  |  |
| Cobiac (2009),  “High income” | Exercise, you only have to take it regularly not seriously. | 6 weeks (model: lifetime) | **Items measured**  -Development: $0.23 mi  -Material: $1.1 mi  -TV: $3.1 mi  -Other (e.g. production of community service announcement): $0.22 mi  -Community service announcements: $7.2 mi  **Total Intervention costs:** $13.3 mi (SD = 1.33 mi)  **Currency (year):** AUD (2003) | **Items measured**  -Material development: $0.497 mi  -Material printing: $2.38 mi  -Television media buy: $6.71 mi  -Other (e.g. production of community service announcement): $0.47 mi  -Community service announcements: $15.58 mi  **Total Intervention costs:** $28.789 mi (SD= 2.878 mi) | $4,798,167  $0.49/person |
| Goryakin (2019),  “High income” | NR. Primary physical activity mass media campaign | 6 segments of 3 years over 31 years | **Items measured:** NR  **Total Intervention costs:** $1.58 per capita annually  **Currency (year):** Euro (2015) | **Items measured:** NR  **Total Intervention costs (currency, year):** $1.07 per capita annually | $0.02/person |
| **CWI with MMC** | | | | | |
| Roux (2008),  “High income” | Wheeling Walks | 8 weeks (model: 40 years) | **Items measured:** NR  **Total Intervention costs:** NR | NA | NA |
| De Smedt (2011),  “High income” | 10 000 Steps Ghent | 20 years | **Items measured**:  -Materials: $NR  -Website: $NR  -Staff: $NR  -Other (Pedometers for 16.4% of population): $NR  **Total Intervention costs**  -First year: $3.51/person  -2nd-5th year: $0.23/person  This 5-year cycle was repeated over a time horizon of 20 years  **Currency (year):** Euro (2009) | **Total Intervention costs**  -First year: $3.17/person  -2^nd^-5^th^ year: $0.20/person | First year: $0.06/person  2^nd^-5^th^ year: $0.001/person |
|  |  |  |  |  |  |
| Mizdrak (2020),  “High income” | NR. Campaign to promote use of PA apps. | 1 year (assumed by reviewers) | **Items measured**  -One-off costs for the promotion of the smartphone apps: $72,000  -Overall (Mass media promotion): $2,791,000  -Identifying top apps: $20,000  **Total intervention cost:** $2,883,000  **Currency (year):** NZD 2011 | **Items measured**  -One-off costs for the promotion of the smartphone apps: $116,213.15  -Mass media promotion: $3,117,559.7  -Identifying top apps: $32,281.43  **Total intervention cost:** $4,653,368 | $89,488 |
| **Costing studies** |  |  |  |  |  |
| **Mostly MM** |  |  |  |  |  |
| Hillsdon (2001)  “High income” | ACTIVE for LIFE  Primary PA | 3 years (156 weeks) | **Items measured**  TV (television advertisement cost): £2 million  **Total Intervention costs:** NR  **Currency (year):** Pound Sterling, 1996 | **Items measured**  Television advertisement cost: $2,228,356  **Total Intervention costs:** NR | $14,284 |
| Bauman (2003),  “High income” | Push Play  Primary PA | 4 years (208.7 weeks) | **Items measured:** NR (Marketing firm assisting with the campaign and other supportive events)  **Total Intervention costs:** $3 million over the four years, with additional in-kind support (amount NR)  **Currency (year):** NZD (1999-2002) | **Items measured:** NR (Marketing firm assisting with the campaign and other supportive events).  **Total Intervention costs:** $6,208,454 over the four years, with additional in-kind support (exact amount NR) | $31,966 |
| Huhman (2010),    “High income” | VERB campaign  Primary PA | 4 years and 3 months (226 weeks) | **Items measured:** NR  **Total Intervention costs:** $339 million  **Currency (year):** USD (2002-2006) | **Items measured:** NR  **Total Intervention costs:** $435,221,288 | $1,925,758 |
| Buchthal (2011),  “High income” | Step it up, Hawaii  Primary Healthy Living (PA and healthy eating) | 10 weeks | **Items measured:**  -TV: $ 100,000  -Radio: $50,000  -Materials: $51,000  **Total Intervention costs** ^Ϫ^**:** $201,000  **Currency (year):** USD (2007) | **Items measured:**  -Television advertising: $124,823  -Radio advertising: $62,412  -Print advertising (in-kind): $63,660  **Total Intervention costs:** $250,894 | $25,089 |
| Leavy (2013),    “High income” | Find Thirty every day  Primary PA | 2.5 years (130 weeks) | **Items measured:** NR  **Total Intervention costs:** $1,800,000  **Currency (year):** AUD (2008-2010) | **Items measured:** NR  **Total Intervention costs:**  $3,201,026 | $24,623 |
| King (2013),    “High income” | Measure-up  Primary Obesity Prevention, with a view of increasing awareness of ‘waist circumference as a risk factor for chronic disease’) | 4 years (208 weeks) (current study presents results for 6 months but costs were reported for 4 years) | **Items measured:** NR  **Total Intervention costs:** $30,000,000  **Currency (year):** AUD (2008-2012) | **Items measured:** NR  **Total Intervention costs:**  $50,749,577 | $243,988 |
| Kite (2018),    “High income” | Make healthy Normal  (Phase 1)  Primary active living and Healthy eating | 1 year (52 weeks) | **Items measured:**  -Overall (media costs): $2,600,000,  -Development: $900,000  **Total Intervention costs:** $3,500,000  **Currency (year):** AUD (2015-2016) | **Items measured:**  - Media costs: $4,074,626  -Development: $1,428,459  **Total Intervention costs:** $5,485,073 | $105,482 |
| Kite (2020),    “High income” | Make healthy Normal  (Phase 2)  Primary active living and Healthy eating | 1 year (52 weeks) | **Items measured:**  -TV: $450,000 (30%)  -Radio, Website/Social media/Other (Radio and out-of-home displays, digital display and search engine optimization, social media and media partnerships): between 5-10% of the remaining been allocated to each.  **Total Intervention costs:** $1,500,000  **Currency (year):** AUD (2017-2018) | **Items measured:**  -Television advertisements: $754,189 (30%)  -Radio and out-of-home displays, digital display and search engine optimization, social media and media partnerships: between 5-10% of the remaining been allocated to each.  **Total Intervention costs:** $2,262,567 | $43,511 |
| Berry (2020),    “High income” | ParticipACTION’s 150 Play List  Primary PA | 51 weeks | **Items measured:**  -Overall (paid media): $2,000,000  -Other (events): $250,000  **Total Intervention costs** ^Ϫ^**:** $2,250,000  **Currency (year):** CAD (2017) | **Items measured:**  Paid media: $2,532,823  Events: $316,603  **Total Intervention costs:** $2,849,426 | $55,871 |
| **CWI with MMC** |  |  |  |  |  |
| Mahecha Matsudo (2003),  “Upper middle-income country” | Agita São Paulo  Primary PA | 1996 (52 weeks and 1 day) | **Items measured:**  -Materials: $77,520  -Staff: $53,200  -Other: $21,280  **Total Intervention costs:** NR  **Currency (year):** USD (1996) | **Items measured:**  -Material: $127,912  -Human resources: $87,783  -Other: 35,113  **Total Intervention costs:** NR | NA |
| Wray (2005),  “High income” | Walk Missouri  Primary PA | 5 months (21.7 weeks) | **Items measured:**  -Billboards: $2,760  -Newspapers: $5,862  -Radio: $9,876  -Materials: $800  **Total Intervention costs: unclear** $19,298  **Currency (year):** USD (2003) | **Items measured:**  Billboards - $3,883  Newspapers - $8,247,  Radio - $13,895  Posters - $1,126  **Total Intervention costs:** $27,150 | NA |
| Brown (2006),  “High income” | 10,000 Steps Rockhampton  Primary PA | 2 years (104 weeks and 2 days) | **Items measured:**  -Development and dissemination: $70,000 ($20,000 with a further $50,000 in-kind contributions)  -Overall: $560,000 or $14 per adult resident  **Total Intervention costs: unclear** $800,000, with additional in-kind support (amount NR)  **Currency (year):** AUD (2002-2003) | **Items measured:**  -Advertising and marketing: $151,526 ($43,306 with a further $108,220 in-kind contributions)  -Intervention costs: $1,212,205 or $30.31 per adult resident  **Total Intervention costs:** $1,731,721 with additional in-kind support (amount NR) | NA |
| Reger-Nash (2006),    “High income” | BC Walks  Primary PA | 8 weeks | **Items measured:**  -Media costs: $126,676 ($70,895 on television advertisements, $13,900 on cable television advertisements, $9675 on newspaper advertisements, $32,186 on radio advertisements  -Staff: $29,000  **Total Intervention costs:** $155,656, $4.31/person  **Currency (year):** USD (2003) | **Items measured:**  -Media costs: $178,221 ($99,742 on television  advertisements)  $19,556 on cable television advertisements)  $13,612 on newspaper advertisements)  $45,282 on radio advertisements  -Personnel costs: $40,800  **Total Intervention costs:** $218,993  $6.06/person | $27,374  $0.75/person |
| Stackpool (2006),    “High income” | Make a move  Primary PA | 3 years (156 weeks) | **Items measured:** NR  **Total Intervention costs:** $2,199,250 (with NSW Health contributing $581,250 (26%) and Area Health Service providing $1,618,000 (74%).  **Currency (year):** AUD (2000-2003) | **Items measured:** NR  **Total Intervention costs:** $4,760,609 (with NSW Health contributing $1,237,758 (26%) and Area Health Service providing $3,522,851 (74%) | $30,517 |
| Peterson (2008),  “High income” | Get up and do something | 6 weeks | **Items measured:** NR  **Total intervention costs:**  Total cost: $340,000 (calculated by reviewers)  Cost/person to become more active by media exposure:  -TV ad #1 only: $42.55  -TV ad #2 only: $153.19  -Billboards only: $5.11  -TV ads #1, #2 only: $25.79  -TV ads #1, #2, billboards: $12.06  -TV: $31.54  -Entire campaign: $8.87  **Currency (year)**: USD (2004) | **Items measured:** NR  **Total intervention costs:**  Total cost: $465,874 (calculate by reviewers)  Cost/person to become more active by media exposure:  -TV ad #1 only: $58.30  -TV ad #2 only: $209.90  -Billboards only: $7.00  -TV ads #1, #2 only: $35.33  -TV ads #1, #2, billboards: $16.52  -Television total: $43.21  -Entire campaign: $12.15 | $56,667  $0.51/person |
| **Mostly CWI with supportive MM or media promotions** | | | | | |
| John-Leader (2008),  “High income” | Stay active Stay independent  Primary PA | 18 months (78.3 weeks) | **Items measured:**  -Newspaper: 5,000  -TV: 3,890  Printing: 2,140  Other: 10,996  in-kind support: $82,000  **Total Intervention costs:** $191,978  **Currency (year):** AUD (2004-2005) | **Items measured:**  -Media costs:  Newspaper: $10,298Television: $8,012  Printing: $4,407  Other: $22,647  in-kind support: $ $168,891  **Total Intervention costs:** $395,407 | $5,050  $0.05/person |
| Huberty (2012),    “High income” | Activate Omaha  Primary PA | 3 years (156 weeks) | **Items measured:** NR  **Total Intervention costs:** $1,635,000 ($185,000 provided by a grant and $1,450,000 in-kind)  **Currency (year):** USD (2005-2007) | **Items measured:** NR  **Total Intervention costs:**  $2,040,857 ($230,923 provided by a grant and $1,809,934 in-kind) | $13,082 |
| Bell (2013),    “High income” | Good for kids  Primary Obesity Prevention | 8 months (34 weeks and 5 days) | **Items measured:** NR  **Total Intervention costs:** $6,000,000 for the 4-year period of the whole campaign ($1,500,000 per annum)  **Currency (year):** AUD (2006-2010) | **Items measured:** NR  **Total Intervention costs:** $10,670,085 for the 4-year period of the whole campaign ($2,667,521 per annum) | $307,495 |
| **MM promotions for single day events, trails, parks** | | | | | |
| Merom (2005),    “High income” | Walk to Work Day  Primary PA | 2003 (2 weeks and 4 days) | **Items measured:**  -TV and radio advertising: $178,356  **Total Intervention costs:** $350,000  **Currency (year):** USD (2003) | **Items measured:**  Television and radio advertising: $250,929  **Total Intervention costs:** $492,415 | $189,391 |
| Clark (2015),    “High income” | Happy trails  Primary PA | 8 weeks | **Items measured:** NR  **Total Intervention costs:** $103,600  **Currency (year):** USD (2011-2012) | **Items measured:** NR  **Total Intervention costs**  $116,784 | $14,598 |

*In cases where multiple interventions were investigated, we are only presented the relevant information for mass media campaign programmes.

Apps: applications, AUD: Australian dollar, CAD: Canadian dollar, CWI: community-wide intervention, mi: million, MM: mass media, MMC: mass media campaign, NA: not applicable, NR: not reported, NSW: New South Wales, NZD: New Zealand dollar, PA: physical activity, OECD SHPeP-NCD: Organization for Economic Co-operation and Development Strategic Public Planning for Noncommunicable diseases, SD: Standard deviation, USD: United States dollar, Wks: weeks. Ϫ Total cost reported, but it is unclear if there were other costs

1. Reger B, Cooper L, Booth-Butterfield S, et al. Wheeling Walks: a community campaign using paid media to encourage walking among sedentary older adults. *Preventive Medicine,* 2002;35:285–92. [↑](#footnote-ref-2)
2. Bauman AE, Bellew B, Owen N, Vita P (2001) Impact of an Australian mass media campaign targeting physical activity in 1998. *American Journal of Preventive Medicine*, 21: 41–47. [↑](#footnote-ref-3)
3. De Cocker KA, De Bourdeaudhuij I, Brown WJ et al. (2007) Effects of ‘10 000 steps Ghent’: a whole-community intervention. *American Journal of Preventive Medicine*, 33, 455–463. [↑](#footnote-ref-4)
4. Goryakin Y, Gatta MS, Lerouge A, Pellegrini T, Cecchini M. The case of obesity prevention in Italy. Ministry of Health, Italy, 2017. [↑](#footnote-ref-5)
5. Cecchini, M., Cortaredona, S., Devaux, M., Elfakir, A., Goryakin, Y., Lerouge, A., Mascarene, C., Moatti, J.P., Paraponaris, A., Pellegrini, T., Sassi, F., Thiebaut, S., Ventelou, B. (2017). Scientific paper on the methodology, results and recommendation for future research. Foresight for health policy development and regulation. <https://www.foresight-fresher.eu/content/uploads/2018/03/d5-3-scientific-paper-on-the-methodology-results-and-recommendation-for-future-research.pdf>. [↑](#footnote-ref-6)
6. Devaux, M., Lerouge, A., Ventelou, B., Goryakin, Y., Feigl, A., Vuik, S., Cecchini, M. (2019). Assessing the potential outcomes of achieving the World Health Organization global non-communicable diseases targets for risk factors by 2025: is there also an economic dividend? *Public Health*, 169: 173-9. [↑](#footnote-ref-7)
7. Bauman AE, Bellew B, Owen N, Vita P (2001). Impact of an Australian mass media campaign targeting physical activity in 1998. *American Journal of Preventive Medicine*, 21: 41–47. [↑](#footnote-ref-8)
8. OECD (2019). The Heavy Burden of Obesity: The Economics of Prevention, OECD Health Policy Studies, OECD Publishing, Paris. [↑](#footnote-ref-9)
9. Hillsdon M., Cavill, N., Nanchahal, K., Diamond, A. & White, I. 2001. National level promotion of physical activity: results from England's ACTIVE for LIFE campaign. *Journal of epidemiology and community health,* 55**,** 755-761. [↑](#footnote-ref-10)
10. Gal R, May AM, van Overmeeren EJ, Simons M, Monninkhof EM. The effect of physical activity interventions comprising wearables and smartphone applications on physical activity: a systematic review and meta-analysis. *Sports Medicine Open,* 2018 Sep 03;4(1):42 [↑](#footnote-ref-11)
11. Matsudo SMM, Matsudo VR, Araújo T, Andrade D, Andrade E, Oliveira L, et al. Nível de atividade física da população do estado de São Paulo: análise de acordo com o gênero, idade, nível sócio-econômico, distribuição geográfica e de conhecimento. *Revista Brasileira Ciência em Movimento*, 2002;10(4):41–50. [↑](#footnote-ref-12)
12. Goryakin Y, Gatta MS, Lerouge A, Pellegrini T, Cecchini M. The case of obesity prevention in Italy. Ministry of Health, Italy, 2017. [↑](#footnote-ref-13)
13. Cecchini, M., Cortaredona, S., Devaux, M., Elfakir, A., Goryakin, Y., Lerouge, A., Mascarene, C., Moatti, J.P., Paraponaris, A., Pellegrini, T., Sassi, F., Thiebaut, S., Ventelou, B. (2017). Scientific paper on the methodology, results and recommendation for future research. Foresight for health policy development and regulation. https://www.foresight-fresher.eu/content/uploads/2018/03/d5-3-scientific-paper-on-the-methodology-results-and-recommendation-for-future-research.pdf [↑](#footnote-ref-14)
14. Devaux, M., Lerouge, A., Ventelou, B., Goryakin, Y., Feigl, A., Vuik, S., Cecchini, M. (2019). Assessing the potential outcomes of achieving the World Health Organization global non-communicable diseases targets for risk factors by 2025: is there also an economic dividend? *Public Health*, 169: 173-9. [↑](#footnote-ref-15)
15. OECD (2019). The Heavy Burden of Obesity: The Economics of Prevention, OECD Health Policy Studies, OECD Publishing, Paris. [↑](#footnote-ref-16)
16. Telfer, K., Wilson, N., Direito, A., Mizdrak, A. Technical report for BODE3 intervention parameter selection: mobile health for physical activity. Technical Report No. 38. Wellington, New Zealand 2020:2020. [↑](#footnote-ref-17)
17. Kvizhinadze, G., Nghiem, N., Atkinson, J., Blakely, T. University of Otago Wellington. 2016. Cost off-sets used in BODE3 multistate lifetable models. [↑](#footnote-ref-18)
18. Mizdrak, A., Blakely, T., Cleghorn, C., Cobiac, L. Technical report for BODE3 active transport and physical activity model. Department of Public Health, University of Otago, Wellington Technical Report No 2018:18. [↑](#footnote-ref-19)
19. OECD (2019). The Heavy Burden of Obesity: The Economics of Prevention, OECD Health Policy Studies, OECD Publishing, Paris. [↑](#footnote-ref-20)
20. Cecchini, M., Cortaredona, S., Devaux, M., Elfakir, A., Goryakin, Y., Lerouge, A., Mascarene, C., Moatti, J.P., Paraponaris, A., Pellegrini, T., Sassi, F., Thiebaut, S., Ventelou, B. (2017). Scientific paper on the methodology, results and recommendation for future research. Foresight for health policy development and regulation. https://www.foresight-fresher.eu/content/uploads/2018/03/d5-3-scientific-paper-on-the-methodology-results-and-recommendation-for-future-research.pdf [↑](#footnote-ref-21)
21. Devaux, M., Lerouge, A., Ventelou, B., Goryakin, Y., Feigl, A., Vuik, S., Cecchini, M. (2019). Assessing the potential outcomes of achieving the World Health Organization global non-communicable diseases targets for risk factors by 2025: is there also an economic dividend? *Public Health*, 169: 173-9. [↑](#footnote-ref-22)
22. Telfer, K., Wilson, N., Direito, A., Mizdrak, A. Technical report for BODE3 intervention parameter selection: mobile health for physical activity. Technical Report No. 38. Wellington, New Zealand 2020:2020. [↑](#footnote-ref-23)
23. Kvizhinadze, G., Nghiem, N., Atkinson, J., Blakely, T. University of Otago Wellington. 2016. Cost off-sets used in BODE3 multistate lifetable models. [↑](#footnote-ref-24)
